# Genome-wide analysis of the cellulose toolbox of *Primulina eburnea*, a calcium-rich vegetable

**DOI:** 10.1186/s12870-023-04266-z

**Published:** 2023-05-16

**Authors:** Yi Zhang, Jie Zhang, Shuaiyu Zou, Ziwei Liu, Hongwen Huang, Chen Feng

**Affiliations:** 1 College of Life Science, Nanchang University, Nanchang, China; 2 Jiangxi Provincial Key Laboratory of ex situ Plant Conservation and Utilization, Lushan Botanical Garden, Chinese Academy of Sciences, No. 9, Zhiqing Rd, Jiujiang, 332900 Jiangxi China; 3 South China Botanical Garden, Chinese Academy of Sciences, Guangzhou, China; 4 University of Chinese Academy of Sciences, Beijing, China

**Keywords:** *Primulina eburnea*, Calcium-rich vegetable, Cellulose biosynthesis, Cell wall, Crop domestication

## Abstract

**Background:**

Human-guided crop domestication has lasted for more than 10,000 years. In terms of the domestication and breeding of vegetables, cellulose content in edible tissues is one of the most important traits. *Primulina eburnea* is a recently developed calcium-rich vegetable with a high soluble and bioavailable calcium content in its leaves. However, the high cellulose content in the leaves hampers the taste, and no research has been reported on the genetic basis of cellulose biosynthesis in this calcium-rich vegetable.

**Results:**

We identified 36 cellulose biosynthesis-involved genes belonging to eight gene families in the *P. eburnea* genome. The cellulose accumulated decreasingly throughout leaf development. Nineteen genes were considered core genes in cellulose biosynthesis, which were highly expressed in buds but lowly expressed in mature leaves. In the nitrogen fertilization experiment, exogenous nitrogen decreased the cellulose content in the buds. The expressing pattern of 14 genes were consistent with phenotypic variation in the nitrogen fertilization experiment, and thus they were proposed as cellulose toolbox genes.

**Conclusions:**

The present study provides a strong basis for the subsequent functional research of cellulose biosynthesis-involved genes in *P. eburnea*, and provides a reference for breeding and/or engineering this calcium-rich vegetable with decreased leaf cellulose content to improve the taste.

**Supplementary Information:**

The online version contains supplementary material available at 10.1186/s12870-023-04266-z.

## Background

Human-guided domestication has occurred independently in different regions around the world starting ~ 12,000 years ago [1, 2]. It is estimated that more than 2500 plant species (over 160 taxonomic families) have undergone domestication worldwide, and approximately 300 species have been fully domesticated [3–5]. Domestication traits arising through artificial selection are desirable to farmers and consumers. For instance, domesticated cereals, once ripened, do not shatter but must be physically separated, and thus easing harvest work. In addition, domestication traits often enhance taste and nutritional qualities. Under conscious selection, different plant organs have been exploited for a variety of uses. Domestication of a fleshy fruit involves color- and flavor-related compounds, with a trend toward an increase in sweetness and a reduction in bitterness and acidity [6]. For carrot, consumers have a desire for giant but less woody roots [7]. For vegetables with edible leaves (e.g., spinach and cabbage), breeding or cultivating objectives are often related to edible fiber, vitamin content, or pathogen resistance. The moderate dietary fiber content of vegetable leaves benefits digestion and intestinal absorption [8], while excessive fiber deposition may reduce taste.

Cellulose, the most abundant biopolymer on the planet, is a major component of plant cell walls [9]. As an important component of water-insoluble dietary fiber, the cellulose content affects the quality, texture and taste of edible tissues of crops [10, 11]. In vascular plants, cellulose microfibrils are synthesized by plasma membrane-localized enzymes called cellulose synthases (CESAs) [12]. There are 10 *CESA* genes in the *Arabidopsis* genome [13]. Genetic evidence and spatiotemporal expression studies have revealed that different *CESA* members function in different types of cell walls. For example, *CESA1*, *CESA3*, and *CESA6* are involved in synthesizing the primary cell wall [14, 15]. In contrast, *CESA4*, *CESA7*, and *CESA8* are required for secondary cell wall formation [16]. Although CESAs play the core catalytic role in cellulose synthesis, many proteins contribute to the activity of or interact with the CESAs. Cellulose synthase interactive protein 1 (CSI1) is the first interactive partner of CESAs identified in higher plants [17]. STELLO proteins (STL1 and STL2) are also CESA accessories [18]. The cellulose synthase companion proteins (CC1 and CC2) have been proposed to function in microtubule formation via direct interaction with CESA complexes [19, 20]. Additional proteins, including KORRIGAN (KOR), CHITINASE-LIKE (CTL), COBRA (COB), and KOBITO (KOB), also play important roles in the regulation of cellulose production and are central components of cellulose synthesis, even though some of them do not necessarily interact with CESAs directly [21]. Mutations in KOR, CTL, COB, and KOB reduce cellulose content and/or cellulose synthesis speed [22–25]. The cellulose content affects crop texture and fruit postharvest shelf life. During crop domestication, many genes involved in cellulose synthesis have evolved or diverged under artificial selection [26]. For example, during selection for increased fiber production, the *CESA1* gene diverged in hemp-type cannabis when compared with basal cannabis [27]. In the fruit breeding and firmness research, cellulose synthesis genes have attracted much attention [28, 29]. In strawberry, cellulose content determines fruit firmness, and members of the *Cellulose synthase* gene family have been proposed to function in fruit development and thereby may be manipulated to enhance fruit storability [29].

Crop domestication from wild resources has never been paused during human history. In the last 100 years, kiwifruit and cranberry have been the most successful domestication cases [30]. For the ongoing process of domestication, in addition to some crops categorized as domesticated, there are also a tremendous number of semi-domesticated crops, such as *Akebia trifoliate* [31, 32], *Hippophae rhamnoides* [33], and *Aristotelia chilensis* [34]. In recent years, some *Primulina* species have been used to develop calcium-rich vegetables with a high content of soluble calcium (Ca^2+^) in their leaves [35, 36].

*Primuline eburnea* (Hance) Yin Z. Wang, which belongs to the Gesneriaceae family, has a great capacity for growth and survival in different environments and has a wide distribution range in southern China [37]. In addition to soluble calcium, its leaves often contain many bioactive substances such as terpenes, flavonoids and phenylethanoid [38], and it was utilized as vegetable for many years in Southwest China. Additionally, this species is highly self-compatible and often produces a large number of flowers and seeds [39]. As reviewed by Huang et al. [30], self-pollinated plants are more easily domesticated than cross-pollinated ones. These make *P. eburnea* an ideal candidate for calcium-rich vegetable development. In recent decades, the potential horticultural importance of *Primulina* has been a driving force in delineating the genetic basis of horticultural traits in this genus. The first genetic linkage map of *Primulina eburnea* based on single nucleotide polymorphism (SNP) markers was constructed in 2016 [40]. Quantitative trait loci (QTL) for some traits was identified in a pair of *Primulina* sister species [41]. Remarkably, the *MYB* gene was first cloned in *Primulina swinglei* (*PsMYB1*), and its identity was confirmed by genetic transformation [42]. *PsMYB1* has been proposed as the transcription factor gene that regulate anthocyanin biosynthesis in *P. swinglei* [42]. However, the genetic basis for vegetable domestication in *Primulina* has not yet been reported. The recent availability of the *P. eburnea* genome [43] provided an opportunity to conduct comprehensive genome-wide identification and analysis of cellulose biosynthetic genes, as reported in the present study. By combining a genome-wide survey of the genes included in the eight gene families involved with cellulose biosynthesis with comparative phylogenetic analyses, we identified 36 *P. eburnea* genes. High-throughput expression profiling of genes in various tissues using RNA-sequencing (RNA-seq) technology and expression patterns of genes in leaves under different treatments using real-time quantitative PCR (qRT-PCR) were combined to identify a cellulose toolbox gene set comprising 14 genes likely involved in cellulose biosynthesis in *P. eburnea*.

## Results

### Changes in cellulose content in P. eburnea leaves

**Fig. 1:**
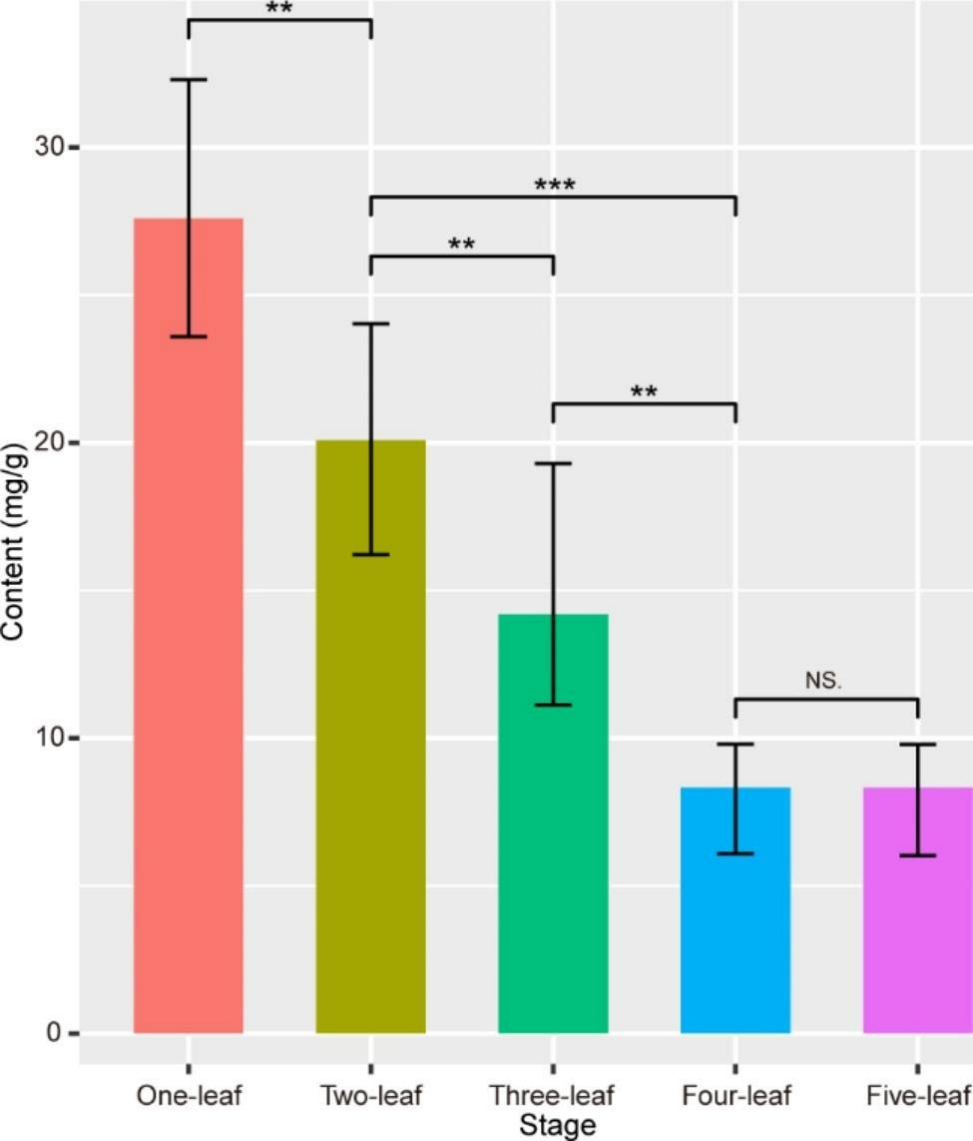
Cellulose contents during the development of *Primulina eburnea* leaves. Mean values and standard deviations (SDs) were obtained from six biological replicates. The error bars indicate standard deviation. Statistical significance was determined by *t*-test. **P < 0.01, ***P < 0.001 and NS. *P* > 0.05

We investigated the cellulose accumulation in the leaves of *P. eburnea* seedlings for that leaves are the main edible tissues of this calcium-rich vegetable (Fig. S1). The cellulose content exhibited L-shaped curves during leaf development (Fig. 1). The cellulose content decreased significantly during the first four stages (*P* < 0.01). No differences were observed between the last two stages (*P* > 0.05). These results indicate that cellulose content decreased during the early development of *P. eburnea* leaves.

To rule out the possibility that cellulose content decreased due to the increasing of water content, water accumulation in leaves were investigated. The water content increased slightly but not significantly during the leaves development (Fig. S2). This indicates that the water accumulation affects the cellulose content little, and the trends of cellulose content biosynthesis during leaf development are credible.

### In silico identification of cellulose biosynthesis-involved genes

A total of 36 genes encoding enzymes involved in cellulose biosynthesis were identified in the *P. eburnea* genome (Table S1). Phylogenetic analyses were conducted with these genes and those retrieved from the genomes of *Arabidopsis thaliana*, *Brassica rapa*, *Cucumis sativus*, *Daucus carota*, *Medicago truncatula* and *Spinacia oleracea*.

### Cellulose synthase (CESA)

**Fig. 2:**
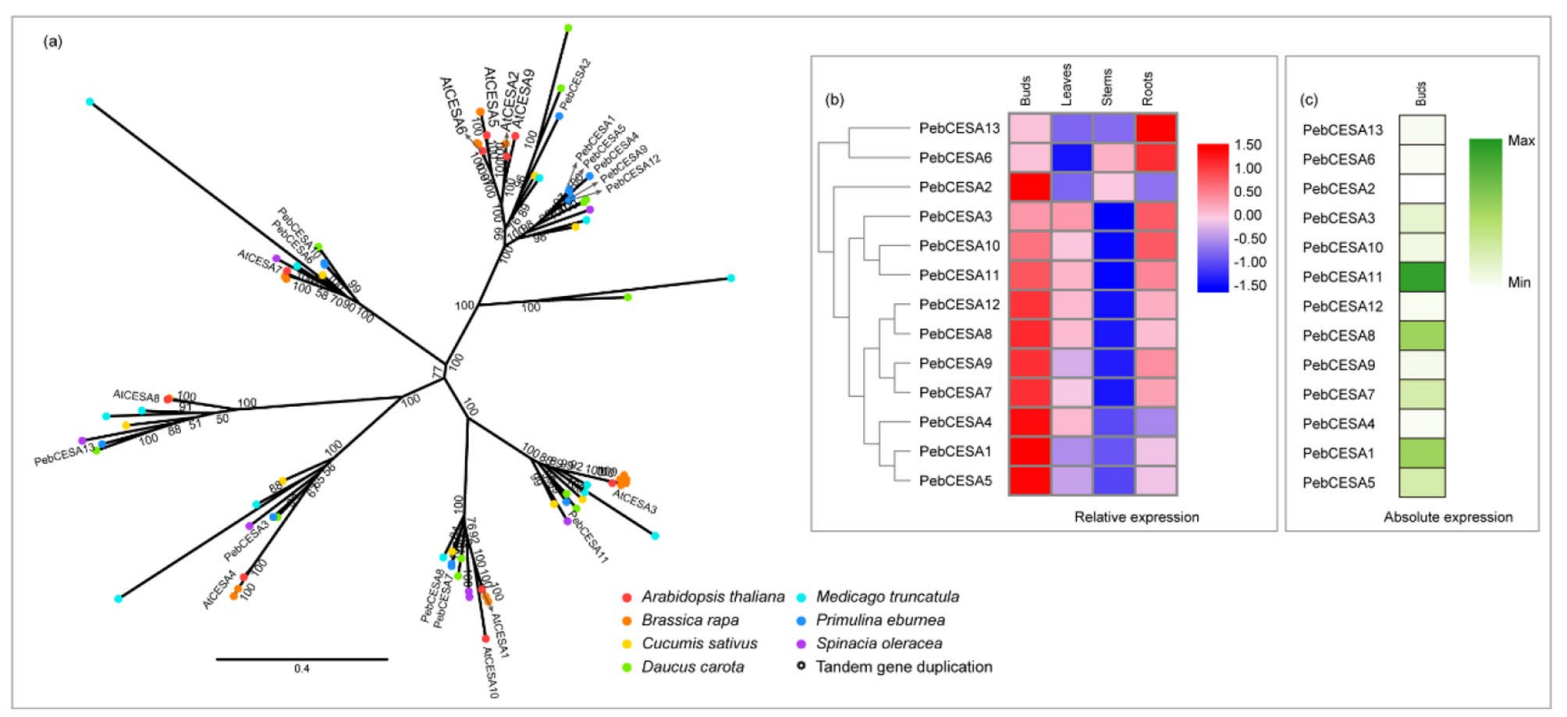
Comparative phylogeny and expression profiles of the cellulose synthase (CESA). **(a)** Unrooted protein phylogenetic tree constructed with CESA sequences from several species. **(b)** Heatmap of transcript accumulation patterns of *PebCESA* genes generated by RNA-seq. **(c)** Heatmaps of *PebCESA* genes expression in *Primulina eburnea* buds

CESA is the only component identified as part of the cellulose synthase complex in higher plants to date [44]. A phylogenetic tree was constructed (Fig. 2a) with CESA protein sequences from seven species. In *P. eburnea*, the CESA family encompasses 13 members, which was similar to that in carrot (12) and alfalfa (13) but less than that in cabbage (19) and more than that in spinach (7), cucumber (8), and *Arabidopsis* (10). Most genes (11 out of 13) were highly expressed in buds and exhibited weak expression in stems (Fig. 2b). In these genes, *PebCESA11* (the most highly expressed member), *PebCESA8*, and *PebCESA1* were more highly expressed than the other genes in buds (Fig. 2c). *PebCESA11* was found to be phylogenetically similar to *AtCESA3*, and *PebCESA8* was similar to *AtCESA1* and *AtCESA10*. *AtCESA1* and *AtCESA3* have been reported to be involved in primary cell wall cellulose synthesis [15]. Both the *cesa1* mutant and *cesa3* mutants are lethal, indicating that CESA1 and CESA3 are indispensable [45]. Little is known about the role of CESA10 [9]. *PebCESA6* and *PebCESA13* showed strong expression in roots, which were phylogenetically similar to *AtCESA7* and *AtCESA8*, respectively. *AtCESA7* and *AtCESA8* participate in secondary cell wall synthesis [16]. Cell division and expansion are more active in buds than in roots which is lignified, and thus primary cell wall formation occurred more often in buds than in roots [46]. The gene expression patterns in *P. eburnea* buds and roots agree with cell wall structure development. In combination with homologous gene function and expression abundance, we predict that *PebCESA11* is the main *CESA* gene involved in cellulose biosynthesis, but the roles of *PebCESA8* and *PebCESA1*, although less prominent, are also likely to be involved.

### Cellulose synthase interactive protein (CSI)

**Fig. 3:**
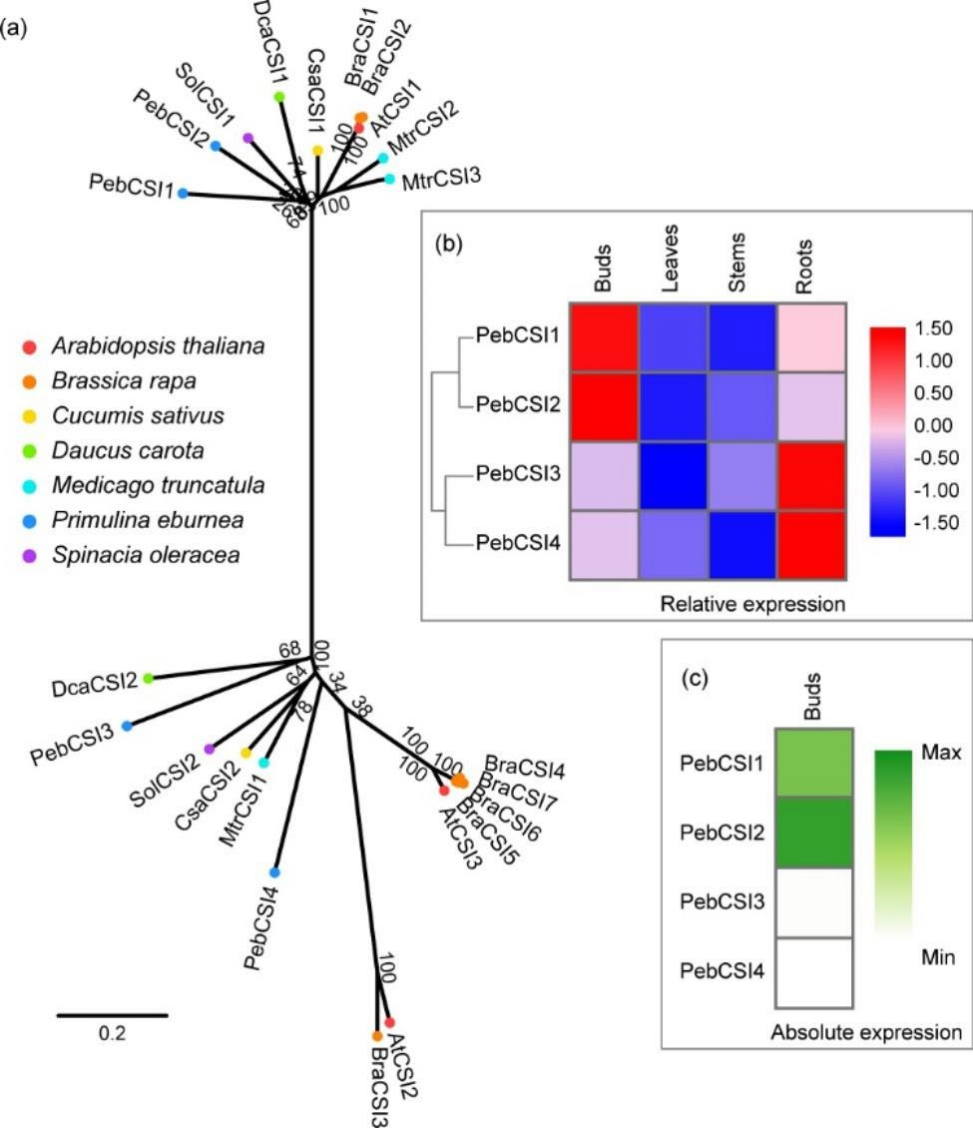
Comparative phylogeny and expression profiles of the cellulose synthase interactive protein (CSI). **(a)** Unrooted protein phylogenetic tree constructed with CSI sequences from several species. **(b)** Heatmap of transcript accumulation patterns of *PebCSI* genes generated by RNA-seq. **(c)** Heatmaps of *PebCSI* genes expression in *Primulina eburnea* buds

CSI1 is the first interactive partner of CESAs identified in higher plants [9]. The CSI family in *Arabidopsis* comprises three members. CSI1 serves as a physical linker between CESA complexes and cortical microtubules [47]. CSI3 is widely expressed in various tissues [48], but CSI2 has not been studied for its function so far. The *P. eburnea* genome had four CSI members (Fig. 3a). RNA-seq profiling highlighted marked distinctions among the four members of this family (Fig. 3b). For instance, *PebCSI1* and *PebCSI2* had similar expression patterns and were highly and preferentially expressed in buds (Fig. 3b, c). The other two members were less expressed in all tissues (Table S2), even though they were preferentially expressed in roots (Fig. 3b). *PebCSI1* and *PebCSI2* were phylogenetically similar to AtCSI1 (Fig. 3a), and they were expressed 175-fold higher than the other two members, on average. They are likely to be involved in cellulose biosynthesis in *P. eburnea*.

### Companion of Cellulose synthase (CC)

**Fig. 4:**
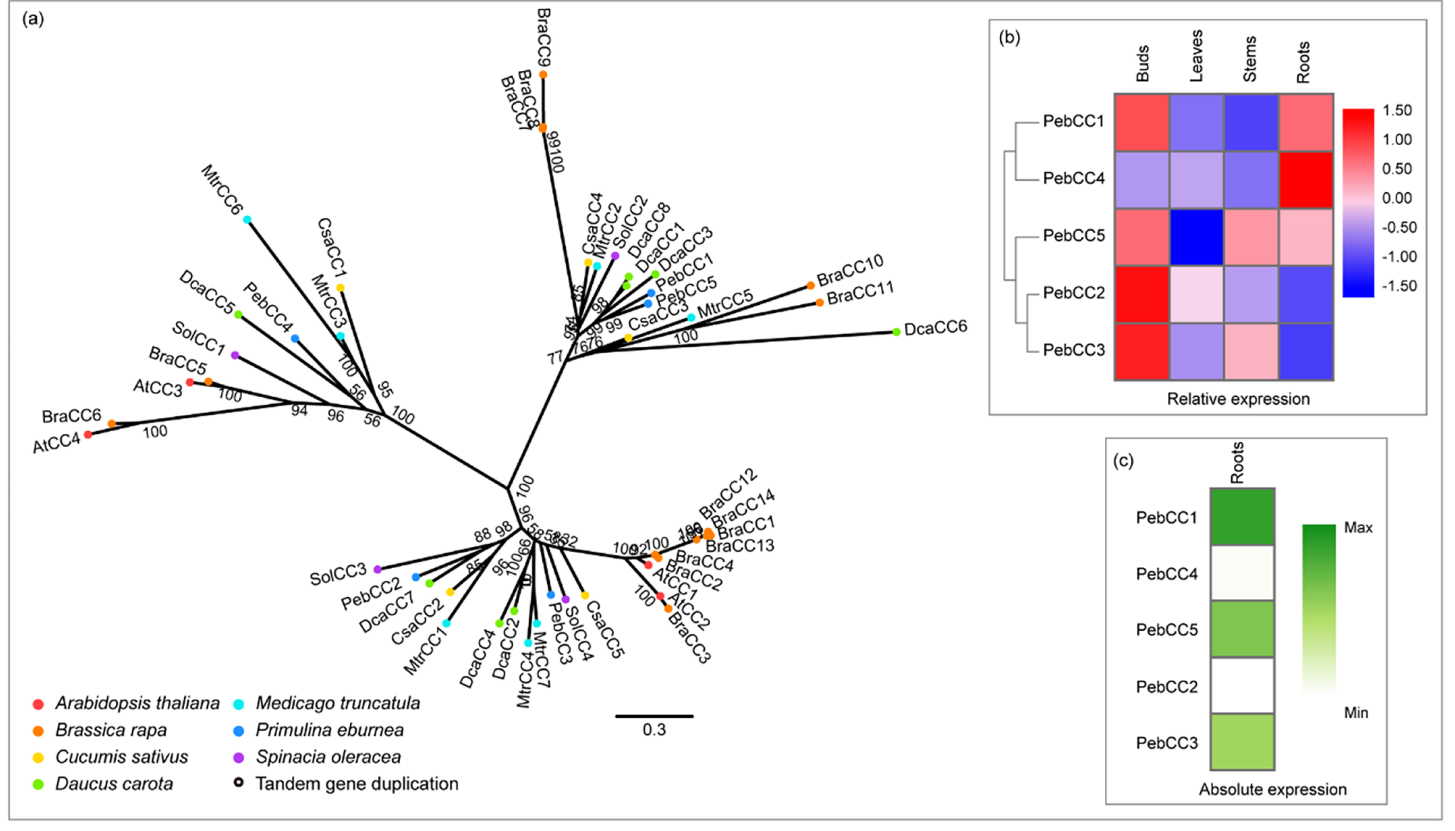
Comparative phylogeny and expression profiles of the companion of cellulose synthase (CC). **(a)** Unrooted protein phylogenetic tree constructed with CC sequences from several species. **(b)** Heatmap of transcript accumulation patterns of *PebCC* genes generated by RNA-seq. **(c)** Heatmaps of *PebCC* genes expression in *Primulina eburnea* buds

The CC protein does not directly affect the co-alignment between CESA complexes and microtubules, which is unlike CSI [20]. CC1 remained associated with CESA complexes under salt stress, and *cc1 cc2* double mutation seedlings displayed stunted growth and cell swelling under adverse conditions. It has been proposed that the CCs function to support the stability of microtubules and CESA complexes under stress conditions [19]. Most plant genomes encode four to eight CCs (Fig. 4a), except cabbage, which harbors 14 members due to the genome triplication [49]. In the five *PebCC* genes, three (*PebCC1*, *PebCC3*, and *PebCC5*) were highly expressed in buds (Fig. 4b, c). PebCC3 is phylogenetically more similar to AtCCs (AtCC1 and AtCC2) than the other two (PebCC1 and PebCC2).

### STELLO (STL)

**Fig. 5:**
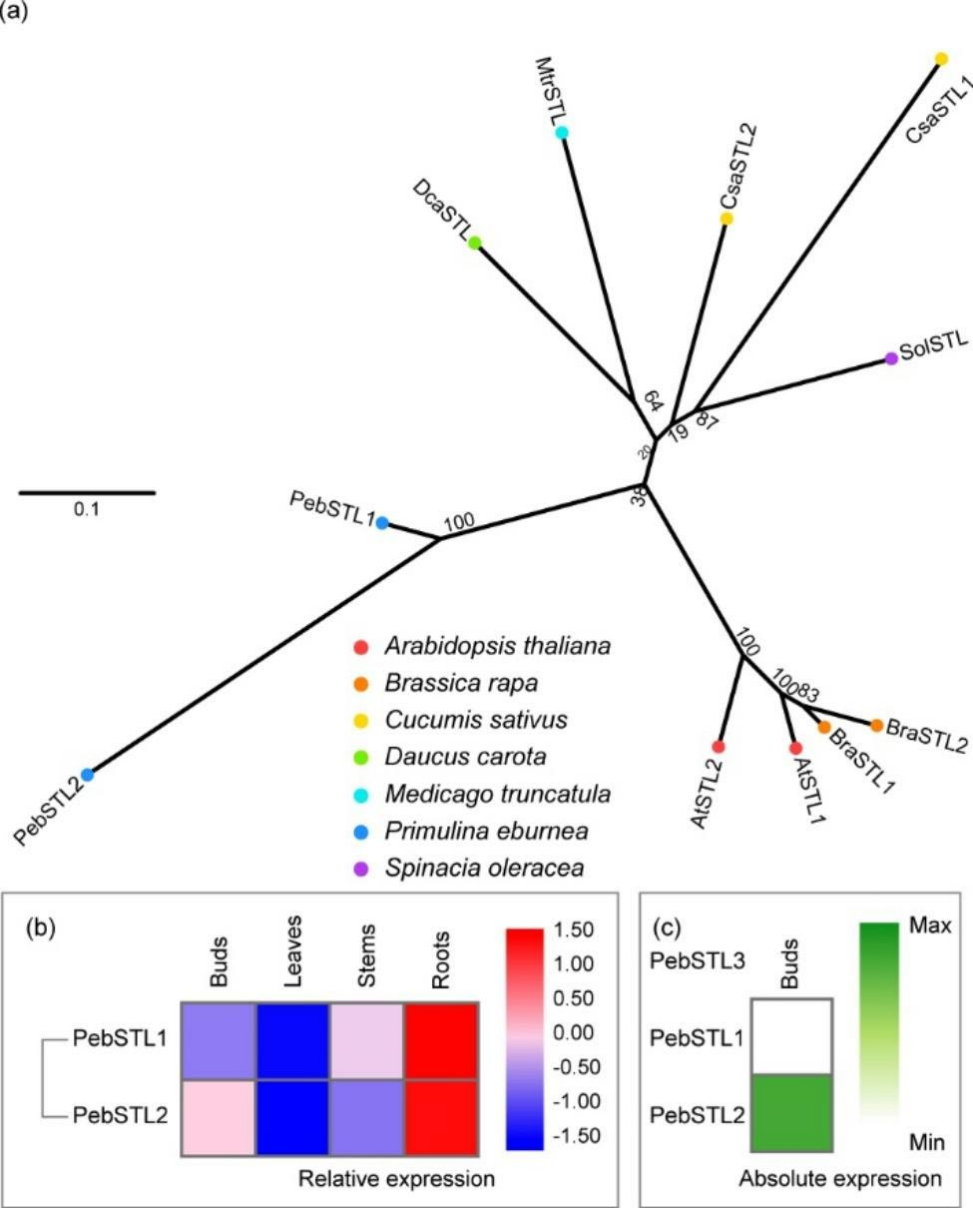
Comparative phylogeny and expression profiles of the STELLO (STL). **(a)** Unrooted protein phylogenetic tree constructed with STL sequences from several species. **(b)** Heatmap of transcript accumulation patterns of *PebSTL* genes generated by RNA-seq. **(c)** Heatmaps of *PebSTL* genes expression in *Primulina eburnea* buds

STL has two members (STL1 and STL2) in *Arabidopsis*, and they localize at the Golgi and interact with CESA [18]. STLs have only been reported in *A. thaliana*, and it has been proposed that STLs impact the secretion and activity of CESA complexes by regulating the assembly of CESA complexes [18]. The *P. eburnea* genome also harbored two STL paralogs (Fig. 5a), sharing 93% identity (Table S3). The two members had similar expression patterns, and both of which were most highly expressed in roots, even though *PebSTL2* was more highly expressed than *PebSTL1* in buds (Fig. 5b, c). They may be more important for the cellulose synthesis in the secondary cell wall than in the primary cell wall.

### COBRA (COB)

**Fig. 6:**
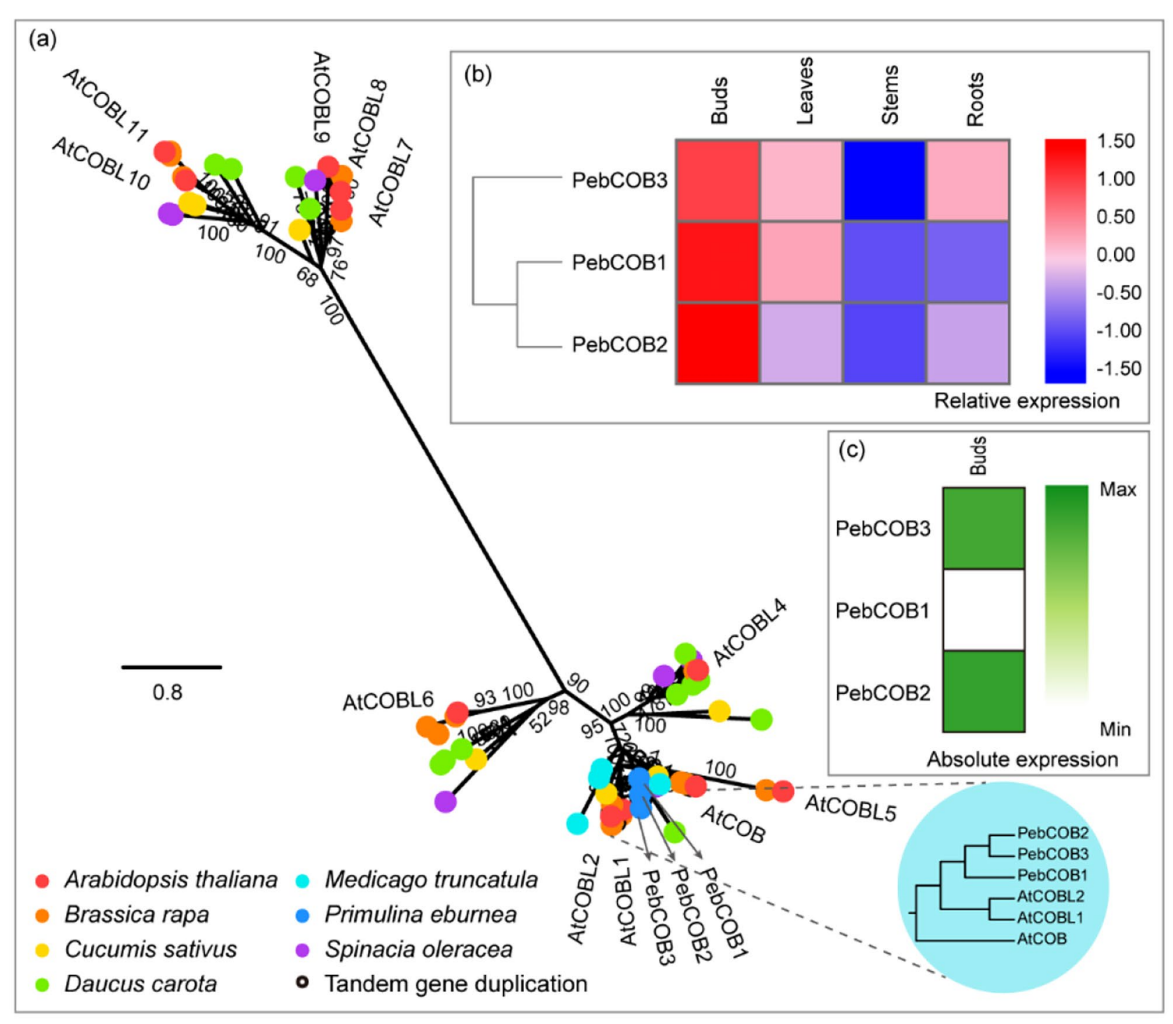
Comparative phylogeny and expression profiles of the COBRA (COB). **(a)** Unrooted protein phylogenetic tree constructed with COB sequences from several species. **(b)** Heatmap of transcript accumulation patterns of *PebCOB* genes generated by RNA-seq. **(c)** Heatmaps of *PebCOB* genes expression in *Primulina eburnea* buds (Note: Bootstrap values can clearly be seen in Additional File 3)

COB, a glycosyl-phosphatidyl inositol-anchored protein, is required for the regulation of cellulose microfibril orientation [50, 51]. It has been detected in multiple cellular compartments [45]. Apart from COB, the COBRA family includes 11 COBRA-Like (COBL) members in *Arabidopsis* [21]. It is a large family found in both dicots and monocots, and its expression may be regulated by environmental stimuli [52, 53]. *COBL4* is indispensable for cellulose synthesis and secondary cell wall formation in *Arabidopsis* and maize [54, 55]. In the analyzed species, *P. eburnea* was identified the least members (three) of the COB family which shared 84.1–95.7% similarity among members (Table S3). Three *COB* genes were expressed in similar patterns in *P. eburnea* tissues and were more abundant in buds and leaves than in rhizomes and roots (Fig. 6a, b). For the absolute expression level, *PebCOB2* and *PebCOB3* were more highly expressed than *PebCOB1* (Fig. 6c; Table S2), supporting their inferred role in cellulose biosynthesis.

### Genes with an unknown function

**Fig. 7:**
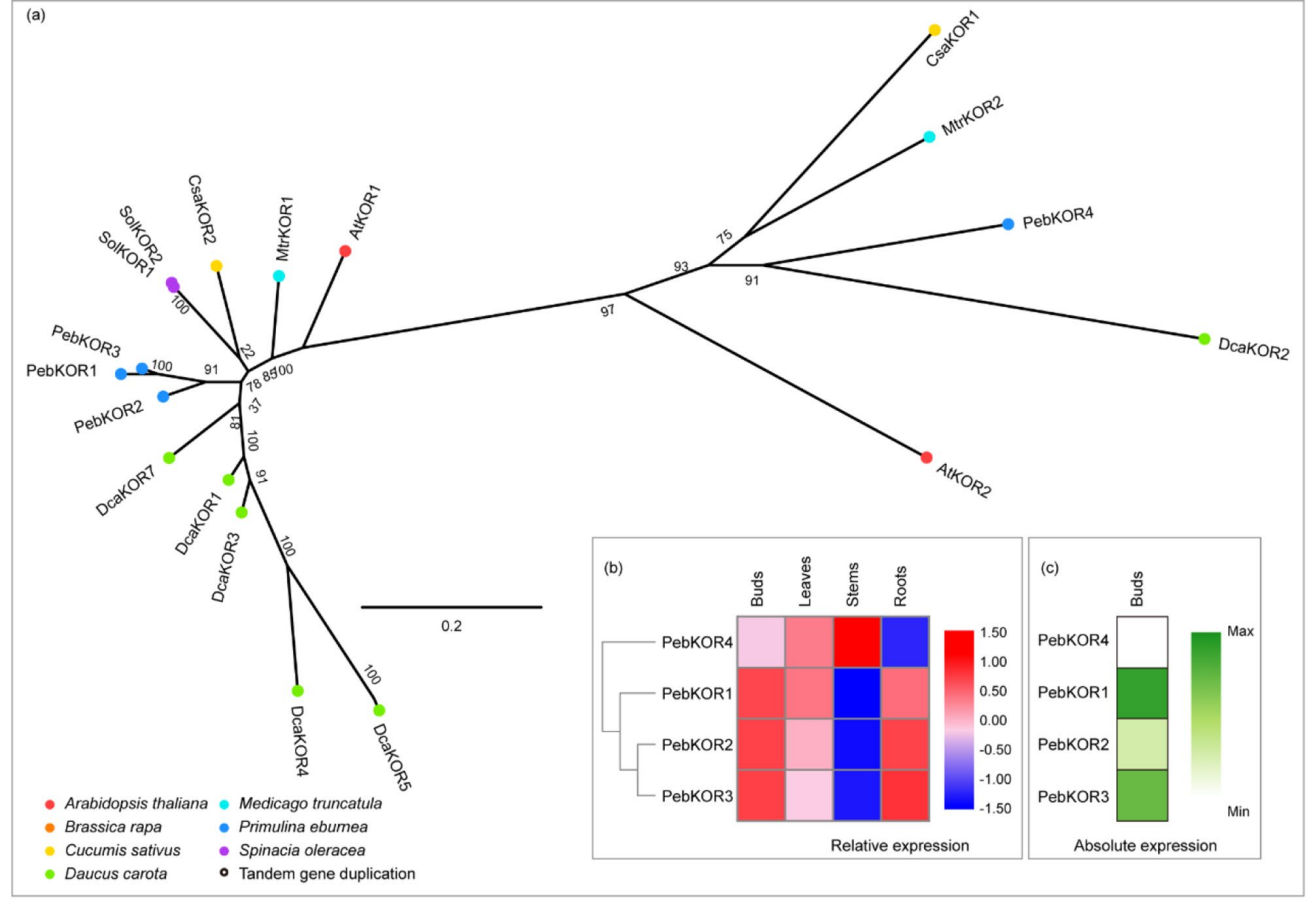
Comparative phylogeny and expression profiles of the KORRIGN (KOR). **(a)** Unrooted protein phylogenetic tree constructed with KOR sequences from several species. **(b)** Heatmap of transcript accumulation patterns of *PebKOR* genes generated by RNA-seq. **(c)** Heatmaps of *PebKOR* genes expression in *Primulina eburnea* buds

KORRIGAN1 (KOR1) encodes a putative membrane-bound β-1,4-endoglucanase [56]. Unlike CSI1, KOR1 co-localizes with CESA complexes in both the plasma membrane and many other endosomal compartments [25, 57]. The mutation phenotype revealed that KOR is required for proper cellulose synthesis in both the primary and secondary cell walls [57, 58]. Despite the close association with CESA complexes, the precise role of KOR1 remains unclear in plants. *A. thaliana* has two single KOR genes [21]. In *P. eburnea*, the KOR family encompassed four members (Fig. 7a). Three (*PebKOR1-3*) had a closer phylogenetic relationship with *AtKOR1* and shared between 88% and 95% protein sequence identity among each other (Table S3). Syntenic analysis showed that this family may have been expanded by genome duplication (Fig. S3). *PebKOR1* and *PebKOR3* were highly expressed in each tissue (Fig. 7b), and their expression was 22-fold (*PebKOR3*) to 24-fold (*PebKOR1*) higher than *PebKOR4* in buds (Fig. 7c; Table S2). These findings support the inferred roles of these two genes in cellulose biosynthesis.

**Fig. 8:**
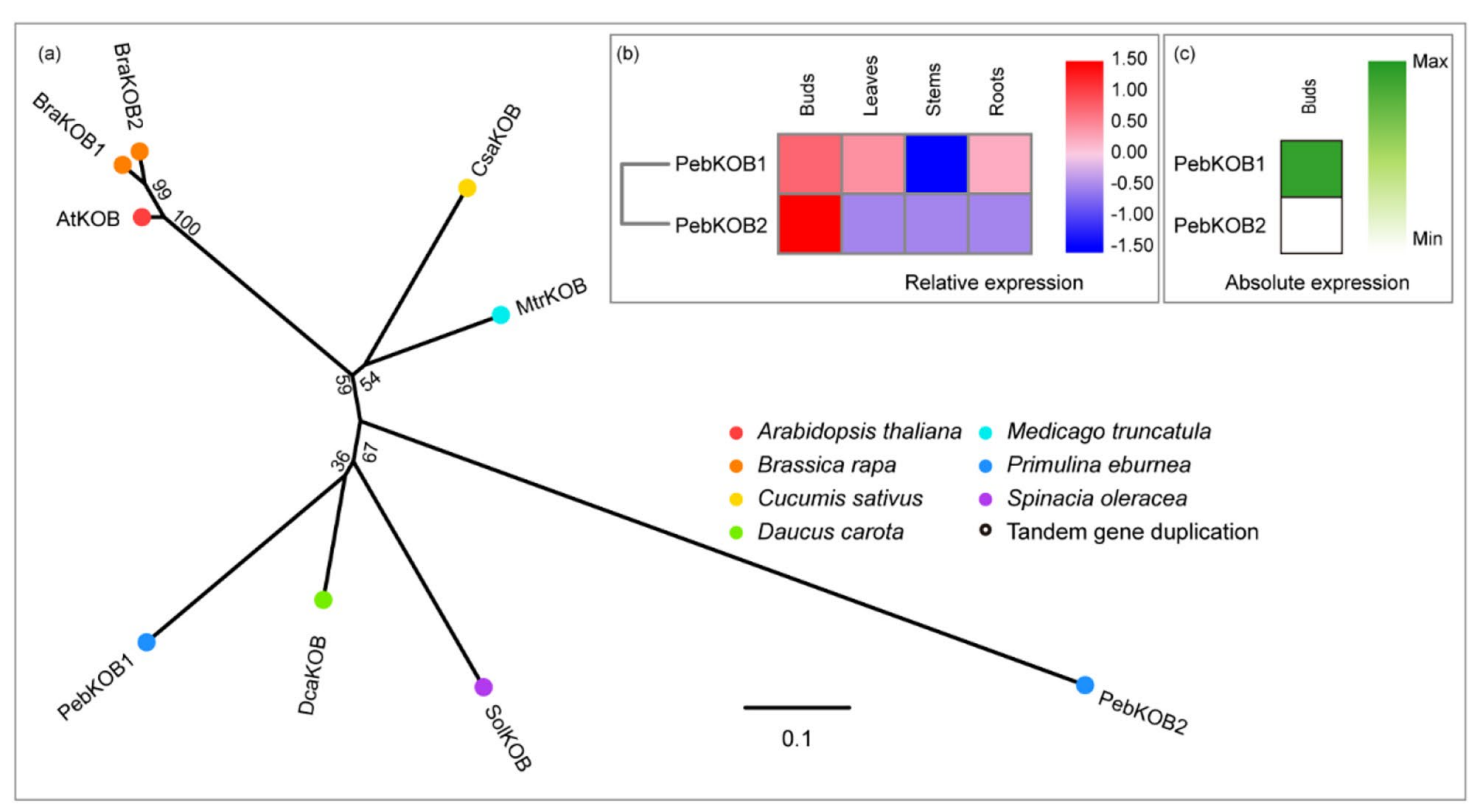
Comparative phylogeny and expression profiles of the KOBITO (KOB). **(a)** Unrooted protein phylogenetic tree constructed with KOB sequences from several species. **(b)** Heatmap of transcript accumulation patterns of *PebKOB* genes generated by RNA-seq. **(c)** Heatmaps of *PebKOB* genes expression in *Primulina eburnea* buds

KOBITO (KOB) has been predicted to be a type II membrane protein with an N-terminus exposed to the cytosol [22]. The mutants of the KOB gene have shown random cellulose microfibril orientation, resulting in incomplete cell walls [22]. FP-KOB1 localizes to the plasma membrane in elongated epidermal/cortical cells. However, the precise function of KOB1 requires further investigation. *A. thaliana* has a single KOB gene. The *P. eburnea* genome had two KOB members (Fig. 8A), which shared 64% similarity (Table S3). *PebKOB1* was more highly expressed than *PebKOB2* in each tissue, even though both showed high expression in buds in their own expression profiles (Fig. 8b, c; Table S3).

**Fig. 9:**
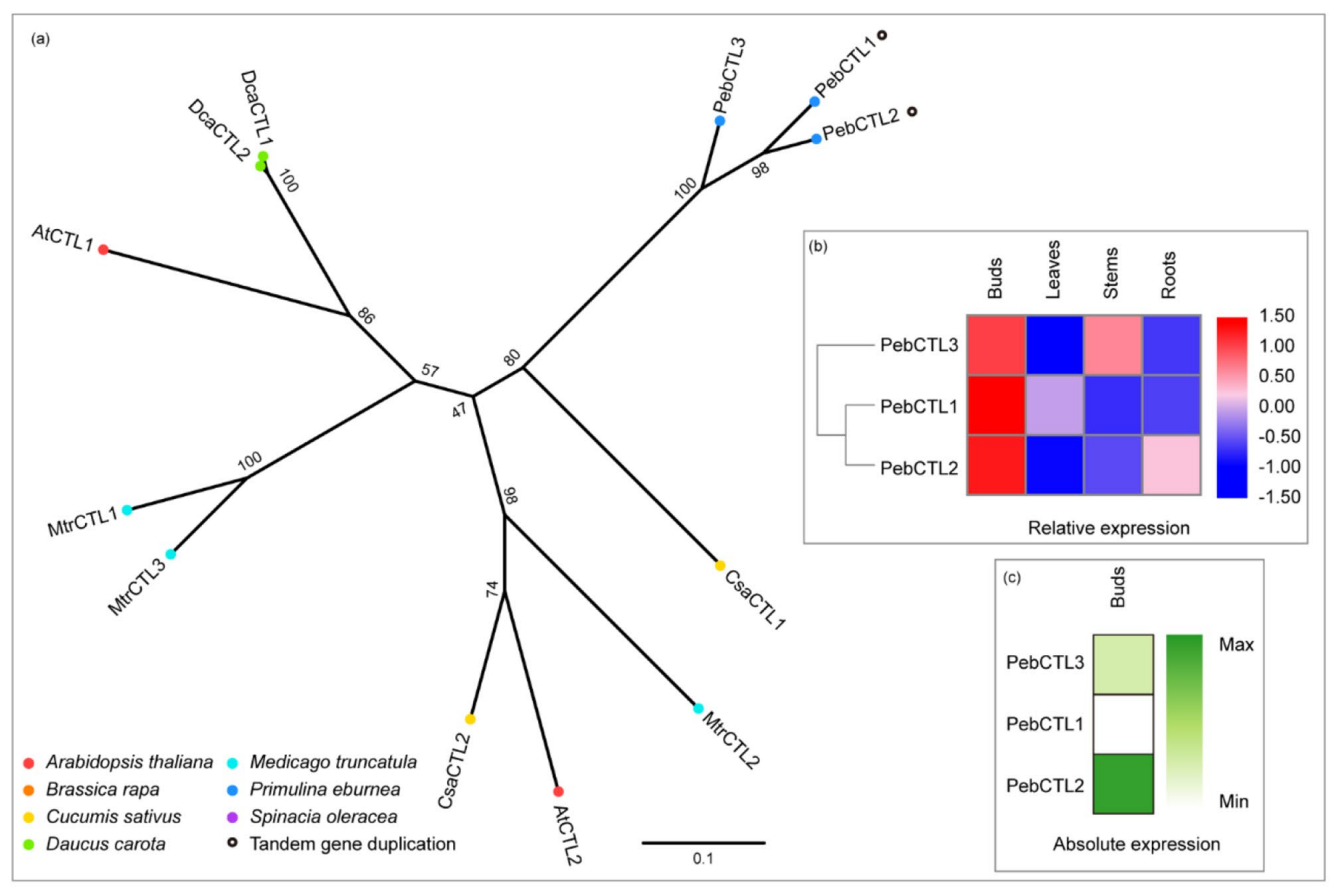
Comparative phylogeny and expression profiles of the CHITINASE-LIKE (CTL). **(a)** Unrooted protein phylogenetic tree constructed with CTL sequences from several species. **(b)** Heatmap of transcript accumulation patterns of *PebCTL* genes generated by RNA-seq. **(c)** Heatmaps of *PebCTL* genes expression in *Primulina eburnea* buds

The CHITINASE-LIKE (CTL) family has two members in *Arabidopsis* and both are located in the apoplast. CTL proteins may bind to the glucan-based polymer cellulose, and mutations in *CTL1* result in reduced cellulose content and CESA complex speed [24]. CTL is, in general, encoded by small gene family not exceeding three members. The *P. eburnea* genome also has three CTL members (Fig. 9a) sharing 85.5–90.8% identity (Table S3). *PebCTL1* and *PebCTL2* were in a 20-kb genomic region of chromosome 6 (Fig. S4) and had high amino acid sequence identities (90.8%) (Table S3), which may have resulted from recent tandem gene duplication events. The three members were all preferentially expressed in buds (Fig. 9b), and *PebCTL2* was more highly expressed than the other family members (Fig. 9c). Indeed, all of these genes were abundantly expressed in various tissues (Table S2), indicating their important roles in *P. eburnea* development.

### Gene interaction and core toolbox cellulose genes

To build a co-expression network, the *in silico* identified genes involved in cellulose biosynthesis and transcription factors (TFs) in *P. eburnea* were selected, and their interactions were predicted (Fig. S5). In total, 399 TFs from 48 families, such as MYB, AP2/ERF, C2H2, WRKY, bHLH, and bZIP, were predicted to be the potential interacting partners of cellulose biosynthesis involved genes (Fig. S5). Several cellulose biosynthesis-involved genes and TF groups were clustered, such as the *PebCESA1* and *5*-TFs group and the *PebCESA2*, *7*, *PebKOB1*, and *PebCC1*-TFs group. These results indicate that the cellulose biosynthesis might be regulated by different TF regulatory modules in *P. eburnea*.

In view of the fact that CESA complexes were made up of CESA and the other interacting or associating genes, we constructed interactions among the cellulose biosynthesis-involved genes (Fig. S6). Many associated genes, including *PebCSI1*, *2*, *PebCC1*, *PebCOB2*, *3* and *PebCTL3*, were located in pivotal positions. They interacted with highly expressed *CESA* genes.

Combining the analysis of gene and motif structures, phylogeny, expression profiles and gene interactions, we proposed 19 members of the eight families as the most likely major genes involved in cellulose biosynthesis in *P. eburnea*. The core gene set comprises *PebCESA1*, *5*, *7*, *8* and *11*, *PebCSI1* and *2*, *PebCC1*, *3* and *5*, *PebSTL2*, *PebCOB2* and *3*, *PebKOR1*, *2* and *3*, *PebKOB1* and *PebCTL2* and 3.

### qRT-PCR to validate the RNA-seq analysis and to determine toolbox genes

**Fig. 10:**
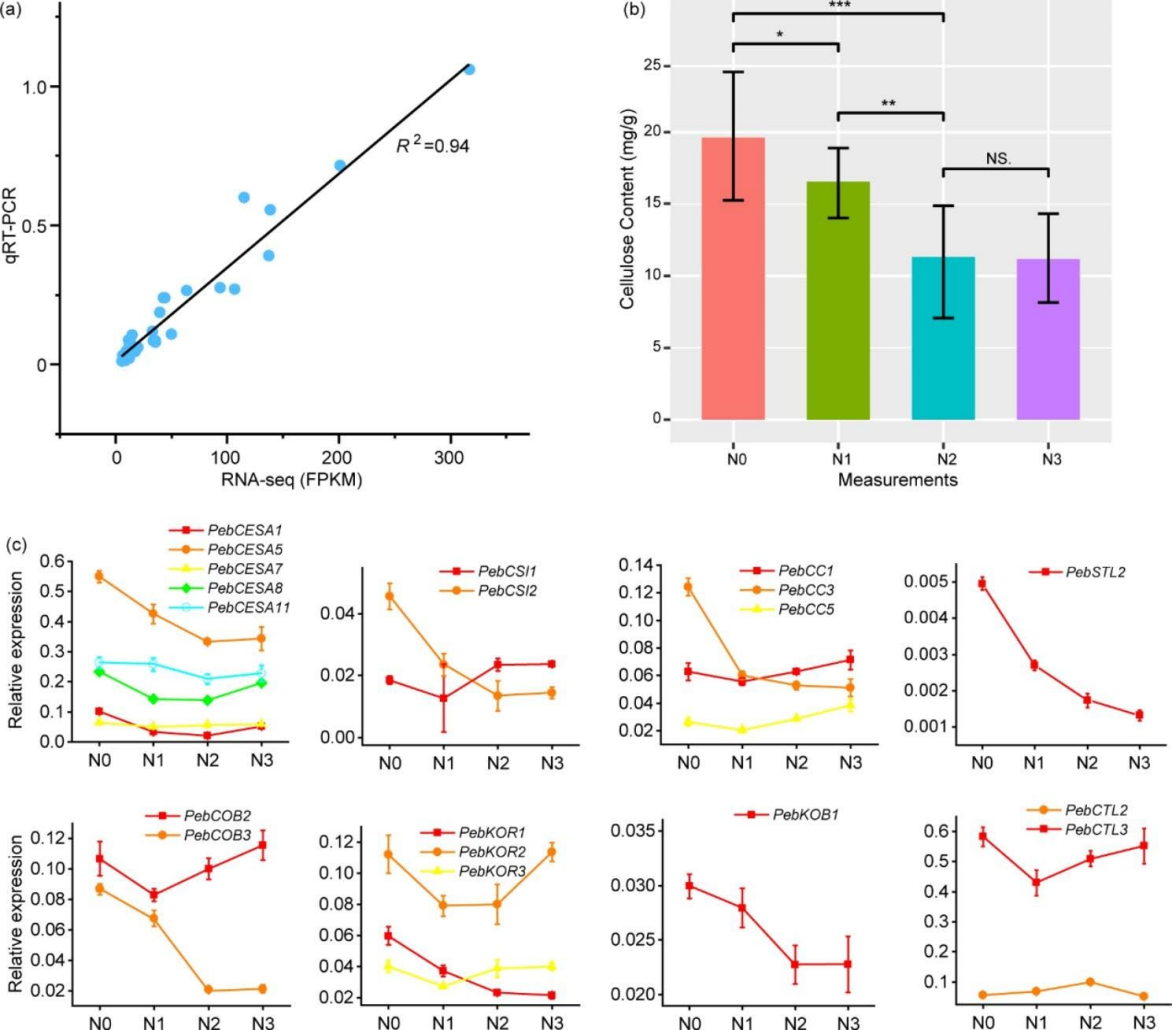
**(a)** Correlation analysis of qRT-PCR and RNA-seq results. **(b)** Changes in the cellulose content of *Primulina eburnea* buds under different concentration of nitrogen. Statistical significance was determined by *t*-test. *P < 0.05, **P < 0.01, ***P < 0.001 and NS. *P* > 0.05. **(c)** The expression pattern of 19 *P. eburnea* cellulose biosynthesis involved genes under different concentration of nitrogen using qRT-PCR. N0, N1, N2, and N3 indicating 0, 5, 10, and 20 g/L urea water solutions, respectively. Error bars represents SD of six independent replicates

To verify the accuracy of the RNA-seq, the mRNA abundances of the eight cellulose synthesis-involved genes were assayed using qRT-PCR. *R*^*2*^ was 0.94 (Fig. 10a; Fig. S7), suggesting consistency between qRT-PCR and RNA-seq.

To examine the response of cellulose content and cellulose biosynthesis toolbox genes to nitrogen fertilization, we treated *P. eburnea* seedlings with 0, 5, 10, and 20 g/L (N0–N3) urea water solutions. Nitrogen fertilization decreased the cellulose content in *P. eburnea* leaves and buds, even though the difference between 10 and 20 g/L nitrogen fertilization was not significant (Fig. 10b). The environmental response of the 19 core genes were investigated to identify toolbox genes. The gene expression profiles, which were more similar to phenotypic variation, were considered as cellulose toolbox genes, including *PebCESA1*, *5* and *11*, *PebCSI2*, *PebCC3*, *PebSTL2*, *PebCOB3*, *PebKOR1*, and *PebKOB1*. These genes belong to seven gene families. The core genes that were not identified as toolbox genes were often highly expressed in the N3 treatment, such as *PebCESA8*, *PebCSI1*, *PebCC1* and *5*, *PebCOB2*, *PebKOR2* and *PebCTL3*. Compared to cellulose biosynthesis, these genes may play more important roles in responding to environmental stimuli. *PebCC1* and *5* and *PebKOR2* showed increasing expression as the rate of nitrogen fertilization increased (Fig. 10c; Table S4). The distinct expression responses to nitrogen fertilization among gene family members may indicate functional diversification within a certain gene family.

## Discussion

Building on the recent availability of the *P. eburnea* genome [43], the present study reports a comprehensive genome-wide identification of cellulose biosynthesis gene families in *P. eburnea*. Combining the phylogenetic relationships and expression profiles, we highlighted the evolutionary histories of these families and predicted the toolbox genes through nitrogen fertilization experiments. To the best of our knowledge, this is the first report to identify all key genes involved in cellulose biosynthesis in one species.

The *CESA* gene family is essential for plant growth and development and plays an important role in response to environmental stress [12]. To date, detailed genome-wide identification of *CESA* genes has been reported in various plant species, such as Arabidopsis [15], rice [59], pineapple [60], tomato [28], pear [61], dropwort [11] and diploid strawberry [29]. We totally identified 13 *CESA* genes in the *P. eburnea* genome, the number was similar to that found in rice (11) but more than that in strawberry (8) and pineapple (8) and less than that in pear (19). The number of *CESA* members identified in diploid *P. eburnea* (2*n* = 36) [62] was the same to that found in triploid banana (13) [63]. Similar to that in dropwort and rice, the expanded copy number of *CESA* in *P. eburnea* may be contributed by the genome duplications [11, 59]. For the other seven gene families, there are no reports on their identification except in Arabidopsis. Most of these gene families are small in *P. eburnea*, and their members are similar to those found in Arabidopsis.

It has been reported that genes duplicated by syntenic duplications or whole genome duplications are much more common than the genes duplicated by tandem duplication in a closely related species, such as *P. huaijiensis* [64]. The *CESA* gene family presented no traces of tandem duplication but may have undergone expansion through whole genome duplication (Figs. S1-S2). For example, the *PebCESA1, 4, 5, 9* and *12* genes each have two syntenic members which were all clustered into a monophyletic clade. In the other seven families, at least four were expanded by whole genome duplication, but only one tandem duplication was found in *PebCTLs* (Figs. S1-S2). These were consistent with the phenomenon in *P. huaijiensis*, which has only 6.2% of genes resulting from tandem duplication [64]. In general, gene duplication is a primary mechanism for functional diversification, and the divergent expression among duplicated genes results in morphological diversification [65]. Different cellulose synthase complexes synthesize microtubules to make up cell walls in different cell types, such as primary cells and secondary cells [9, 21]. In a particular tissue or in a particular environment, there is only a small proportion of gene members (often one or two) highly expressed in each family. In the cellulose biosynthesis-involved gene families, only a few members show similar expression patterns to the phenotypic variation trends. The other gene members may participate in cellulose biosynthesis in response to environmental stimuli.

A different situation was found in the CTL family, where no core genes were expressed with phenotypic variation in nitrogen fertilization measurements (Fig. 10b, c). In Arabidopsis, CTL has been reported to have a role in the CESA complex and to affect the cellulose content [24]. The function of *AtCTL1* and *AtCTL2* may have been diverged because they are expressed with different cell types [20]. However, the precise function of CTLs requires further investigation [21, 24]. In *P. eburnea*, neither *PebCTL2* nor *3* was expressed with phenotypic variation in the nitrogen fertilization treatments (Fig. 10c), even though the expression of both decreased throughout leaf development. However, we cannot rule out the possibility that the limited development stages we sampled affected the characterization of expression profiles.

Exogenous nitrogen often affects the cellulose content in crops, such as cotton [66], wheat [67], and rapeseed [68]. In this study, we investigated the effect of nitrogen fertilization on the cellulose content in *P. eburnea* leaves. Cellulose accumulation decreased with the rate of nitrogen fertilization (Fig. 10b). This indicates that nitrogen fertilization may be an alternative method to decrease the cellulose content in this calcium-rich vegetable. This phenomenon is similar to that observed in wheat stems [67] and rapeseed roots [68]. However, a contrasting result has been reported in cotton; that is, an increased rate of nitrogen fertilization increases the fiber yield by accumulating cellulose [66].

For the eight cellulose biosynthesis-involved gene families, we combined comparative phylogeny with individual gene development expression profiling characterized by both RNA-seq and qPCR. By using this approach, we identified nine genes likely to be involved in cellulose biosynthesis in *P. eburnea* leaves, constituting the so-called “cellulose toolbox”. The qPCR results showed that each gene family had members with decreased expression after the nitrogen treatment, except for the *PebCTL* family. The downregulation of these genes may contribute to the decrease cellulose accumulation in *P. eburnea* buds. Similar to our results, it has also been reported that nitrogen fertilization results in decreased expression of CESA genes in wheat [67]. In rapeseed, CESA genes are upregulated by nitrate (NO_3_^–^) but downregulated by ammonium (NH_4_^+^) [69]. Together with the results on cellulose accumulation, these results indicate that the effect of nitrogen fertilization on the cellulose biosynthesis is species or lineage specific. Except for the nine toolbox genes, the 27 remaining genes probably contribute to the response to environmental cues or are involved in cellulose biosynthesis in other tissues. To the best of our knowledge, most of these genes were first identified in non-model plants. As these genes have not been reported before, this enriches our knowledge of cellulose biosynthesis in *P. eburnea*.

In summary, our study of cellulose biosynthesis-involved genes provides a strong basis for understanding the biosynthetic mechanisms of cellulose in *P. eburnea*. Furthermore, the cellulose toolbox genes pave the way for future functional studies and can be candidate genes for breeding and/or engineering this calcium-rich vegetable with decreased cellulose content which improves its texture and taste.

## Conclusions

Thirty-six cellulose biosynthesis-involved genes were identified through a genome-wide survey, analyses of gene and motif structures, and phylogenetic analysis in *P. eburnea*. Nineteen core genes were identified in the gene expression profiles via RNA-seq in various tissues. Cellulose accumulated decreasingly throughout leaf development, and its content decreased when treated with nitrogen fertilizer. The expression profile of core genes under nitrogen treatment revealed that the cellulose toolbox comprised 14 genes belonging to seven families, and genes not included in the toolbox could contribute to the response to environmental cues. Most of these genes were first identified in non-model plants. This study provides a data source for the subsequent functional studies of cellulose biosynthesis-involved genes in *P. eburnea* and provides a reference for decreasing leaf cellulose in this calcium-rich vegetable.

## Methods

### Plant materials

*Primulina eburnea* seedlings were grown from the seeds of cultivar ‘Gaogai-01’. Seeds were sown in peat soil on January 6, 2022. Seedlings were transplanted in plastic pots (12 cm × 12 cm) filled with a 4:1 mixture of peat soil and vermiculite on March 2, 2022. The plants were placed in an incubator with temperatures of 25/18 ℃ (day / night), 65–70% humidity, and an 10 h photoperiod of 6000 lx at Lushan Botanical Garden, Chinese Academy of Sciences, Nanchang, Jiangxi China (115.8382°E; 28.9112°N). Three months later, 20 healthy seedlings with 5–6 pairs of leaves were randomly selected to measure the cellulose content of their first to fifth pairs of leaves. The buds (second pair of leaves), leaves (fourth pair of leaves), rhizomes, and roots were used for RNA-seq analysis. At the same time, some seedlings were treated with 100 ml of urea solution (5, 10, and 20 g/L) per week for nitrogen fertilization; water treatment was used as a control. The buds and leaves were collected after 1 month of treatment. These samples were used to investigate the effect of exogenous nitrogen fertilization on the cellulose content. The cellulose content data were analyzed for significant differences by *t*-test.

### In silico identification and basic characterization of cellulose toolbox genes

Combining the keyword searches from *P. eburnea* genome annotations and BLASTp searches (using proteins from *Arabidopsis thaliana* as queries), we retrieved the potential protein sequences from *P. eburnea* genome (Text S1). After the analyses of gene and motif structures and conserved domain, we excluded the protein sequences that differed significantly from those of Arabidopsis and other *P. eburnea*. The remaining sequences were used to generate large comparative phylogenetic trees with protein sequences from *Arabidopsis thaliana*, *Brassica rapa*, *Cucumis sativus*, *Daucus carota, Medicago truncatula* and *Spinacia oleracea*. The genome databases for these species were listed in Table S5. For *P. eburnea* short-name gene nomenclature, we adopted the prefix Peb, followed by the multigene family abbreviation (Table S1). The chromosomal location diagrams of *P. eburnea* genes were generated using MapChart 2.2 [70]. Syntenic analysis and collinearity analysis were performed using MCScanX [71] and TBtools [72].

### Phylogenetic analysis

Multiple sequence alignment of the cellulose toolbox gene protein sequences of seven species was performed with MUSCLE v3.8.425 [73]. Maximum likelihood phylogenetic trees were constructed with topological support assessed with 1000 bootstrap replicates using IQTREE v1.6.11 [74]. The best fit model for phylogenetic tree construction was selected automatically by IQTREE.

### RNA-seq expression analysis and network construction

To gain insights into the spatial and temporal expression patterns of cellulose biosynthesis-involved genes, transcriptome sequencing were performed for young and mature leaves, rhizomes and roots. A total of 14 plant libraries (four buds and leaves, three rhizomes and roots) were collected from *P. eburnea* for RNA-seq. Total RNA was extracted using an RNAprep pure Plant Kit (Tiangen, Beijing, China) according to the manufacturer’s instructions, and RNA quality was evaluated by gel electrophoresis. Complementary DNA (cDNA) libraries were constructed and then sequenced using the Illumina NovaSeq 6000 paired-end sequencing system. The libreries construction and sequencing was performed by BioMarker Co., Ltd. (Beijing, China). Reads were quality-filtered by removing adapter sequences and reads containing > 10% low-quality bases with a Q20 value of ≤ 20%. The quality filtration pipeline was provided by Feng et al. [75]. All clean reads were mapped to the reference genome of *P. eburnea* [43] using Hisat2 tools [76]. Fragments per kilobase of exon per million fragments mapped (FPKM) were used to estimate the gene expression levels. The relative expression for each gene member in the four tissues were estimated by log_2_ scaling.

For co-expression network construction, genes with an FPKM > 1 in any of the samples were used for the calculation of the Pearson correlation coefficient (PCC). Only the absolute PCC value > 0.9 was considered as a potential interaction. Then, the potential network was visualized using the Cytoscape software.

All raw Illumina data were deposited in the NCBI Sequence Read Archive under accession number PRJNA934730.

### Quantitative real-time PCR

Eight cellulose biosynthesis-involved genes were randomly selected for reverse transcription-quantitative PCR (qRT-PCR) assays to validate the accuracy of RNA-seq analysis. To detect the effect of nitrogen fertilization on the expression of cellulose biosynthesis-involved genes, seedlings were fertilized with different concentrations of urea water solution for 30 days and qRT-PCR were conducted on the leaf samples with a series of genes involved in cellulose synthesis. cDNAs were synthesized from RNA (1 µg in total) by the TransScript II All-in-One FirstStrand cDNA Synthesis SuperMix for qPCR (TransGen Biotech, Beijing, China) following the manufacturer’s instructions. qRT-PCR was performed using the MonAmpTM ChemoHS qPCR mix kit (Monad Biotech, Wuhan, China) and detected using the BIO-RAD CFX96 real-time PCR detection system (BIO-RAD, Pleasanton CA, USA). The PCR cycling parameters used were: denaturation at 95 °C for 5 min and 42 cycles of 95 °C for 10 s, 56 °C for 20 s, and 72 °C for 30 s. According to the output data, relative expressions of the mRNA of each sample were normalized according to the expression level of the internal reference gene *PebActin*. Three technical and biological replicates were used. The qRT-PCR primers, which were designed using Primer Premier v6.0 (Premier Biosoft, Palo Alto, CA, USA), are listed in Table S6.

## Electronic supplementary material

Below is the link to the electronic supplementary material.

Supplementary Material 1

Supplementary Material 2

Supplementary Material 3

## Data Availability

The transcriptome data has been deposited in the NCBI Sequence Read Archive under accession numbers PRJNA934730. The gene structure and motif analysis results were available on FIGSHARE: 10.6084/m9.figshare.22549852.v1.

## References

[1] Diamond J (2002). Evolution, consequences and future of plant and animal domestication. Nature.

[2] Diamond J, Bellwood P (2003). Farmers and their languages: the first expansions. Science.

[3] Meyer RS, Purugganan MD (2013). Evolution of crop species: genetics of domestication and diversification. Nat Rev Genet.

[4] Fernie AR, Yan J (2019). De novo domestication: an alternative route toward new crops for the future. Mol Plant.

[5] Salman-Minkov A, Sabath N, Mayrose I (2016). Whole-genome duplication as a key factor in crop domestication. Nat Plants.

[6] Shang Y, Ma Y, Zhou Y, Zhang H, Duan L, Chen H (2014). Plant science. Biosynthesis, regulation, and domestication of bitterness in cucumber. Science.

[7] Wang G, Huang Y, Zhang X, Xu Z, Wang F, Xiong A (2016). Transcriptome-based identification of genes revealed differential expression profiles and lignin accumulation during root development in cultivated and wild carrots. Plant Cell Rep.

[8] Fuller S, Beck E, Salman H, Tapsell L (2016). New Horizons for the study of Dietary Fiber and Health: a review. Plant Foods Hum Nutr.

[9] Zhu X, Xin X, Gu Y, Cohen E, Merzendorfer H (2019). Cellulose and hemicellulose synthesis and their regulation. Plant cells in Extracellular Sugar-Based biopolymers matrices.

[10] Li Y, Yu Y, Wu J, Xu Y, Xiao G, Li L (2022). Comparison the Structural, Physicochemical, and Prebiotic Properties of Litchi Pomace Dietary fibers before and after modification. Foods.

[11] Liu J, Liu H, Tao J, Tan G, Dai Y, Yang L (2023). High-quality genome sequence reveals a young polyploidization and provides insights into cellulose and lignin biosynthesis in water dropwort (*Oenanthe sinensis*). Ind Crop Prod.

[12] McFarlane HE, Döring A, Persson S (2014). The cell biology of cellulose synthesis. Annu Rev Plant Biol.

[13] Richmond T. Higher plant cellulose synthases. Genome Biol 1. 2000; reviews3001.1. 10.1186/gb-2000-1-4-reviews3001 PMC138876 11178255

[14] Desprez T, Juraniec M, Crowell EF, Jouy H, Pochylova Z, Parcy F (2007). Organization of cellulose synthase complexes involved in primary cell wall synthesis in *Arabidopsis thaliana*. Proc Natl Acad Sci U S A.

[15] Persson S, Paredez A, Carroll A, Palsdottir H, Doblin M, Poindexter P (2007). Genetic evidence for three unique components in primary cell-wall cellulose synthase complexes in *Arabidopsis*. Proc Natl Acad Sci U S A.

[16] Taylor NG, Howells RM, Huttly AK, Vickers K, Turner SR (2003). Interactions among three distinct CESA proteins essential for cellulose synthesis. Proc Natl Acad Sci U S A.

[17] Gu Y, Somerville C (2010). Cellulose synthase interacting protein: a new factor in cellulose synthesis. Plant Signal Behav.

[18] Zhang Y, Nikolovski N, Sorieul M, Vellosillo T, McFarlane HE, Dupree R (2016). Golgi-localized STELLO proteins regulate the assembly and trafficking of cellulose synthase complexes in *Arabidopsis*. Nat Commun.

[19] Endler A, Kesten C, Schneider R, Zhang Y, Ivakov A, Froehlich A (2015). A mechanism for sustained cellulose synthesis during salt stress. Cell.

[20] Endler A, Schneider R, Kesten C, Lampugnani ER, Persson S (2016). The cellulose synthase companion proteins act non-redundantly with CELLULOSE SYNTHASE INTERACTING1/ POM2 and CELLULOSE SYNTHASE 6. Plant Signal Behav.

[21] Lampugnani ER, Flores-Sandoval E, Tan QW, Mutwil M, Bowman JL, Persson S (2019). Cellulose synthesis–central components and their evolutionary relationships. Trends Plant Sci.

[22] Pagant S, Bichet A, Sugimoto K, Lerouxel O, Desprez T, McCann M (2002). *KOBITO1* encodes a novel plasma membrane protein necessary for normal synthesis of cellulose during cell expansion in Arabidopsis. Plant Cell.

[23] Gu Y, Kaplinsky N, Bringmann M, Cobb A, Carroll A, Sampathkumar A (2010). Identification of a cellulose synthase-associated protein required for cellulose biosynthesis. Proc Natl Acad Sci U S A.

[24] Sánchez-Rodríguez C, Bauer S, Hématy K, Saxe F, Ibáñez AB, Vodermaier V (2012). CHITINASE-LIKE1/POMPOM1 and its homolog CTL2 are glucan-interacting proteins important for cellulose biosynthesis in *Arabidopsis*. Plant Cell.

[25] Vain T, Crowell EF, Timpano H, Biot E, Desprez T, Mansoori N (2014). The cellulase KORRIGAN is part of the cellulose synthase complex. Plant Physiol.

[26] Rubin EM (2008). Genomics of cellulosic biofuels. Nature.

[27] Ren G, Zhang X, Li Y, Ridout K, Serrano-Serrano ML, Yang Y (2021). Large-scale whole-genome resequencing unravels the domestication history of *Cannabis sativa*. Sci Adv.

[28] Song X, Xu L, Yu J, Tian P, Hu X, Wang Q (2019). Genome-wide characterization of the cellulose synthase gene superfamily in *Solanum lycopersicwn*. Gene.

[29] Huang H, Zhao S, Chen J, Li T, Guo G, Xu M (2022). Genome-wide identification and functional analysis of cellulose synthase gene superfamily in *Fragaria vesca*. Front Plant Sci.

[30] Huang H, Zou S, Cheng C (2021). Domestication and breeding strategy of wild fruit trees on track of plant introduction and domestication history. J Plant Genet Resour.

[31] Zou S, Yao X, Zhong C, Gao P, Wang Z, Huang H (2020). Phenotypic characterization of *Stauntonia obovatifoliola* Hayata subsp. *urophylla* germplasm: a potential new fruit crop. Genet Resour Crop Evol.

[32] Zou S, Gao P, Jia T, Huang H (2022). Physicochemical characteristics and nutritional composition during Fruit Ripening of *Akebia trifoliata* (Lardizabalaceae). Horticulturae.

[33] Wang Z, Wang W, Zhu C, Gao X, Chu W (2022). Evaluation of antioxidative and neuroprotective activities of total flavonoids from Sea Buckthorn (*Hippophae rhamnoides* L). Front Nutr.

[34] Sobaszek P, Rozylo R, Dziki L, Gawlik-Dziki U, Biernacka B, Panasiewicz M (2020). Evaluation of Color, texture, sensory and antioxidant Properties of Gels composed of freeze-dried Maqui Berries and Agave Sugar. Processes.

[35] Qi Q, Hao Z, Tao J, Kang M (2013). Diversity of calcium speciation in leaves of *Primulina* species (Gesneriaceae). Biodivers Sci.

[36] Zhang Y, Fu C, Zhou R, Liu Z, Feng C (2023). Effect of prechilling and exogenous gibberellin on seed germination of *Primulina eburnea*: a calcium-rich vegetable. Seed Sci Technol.

[37] Wang J, Ai B, Kong H, Kang M (2017). Speciation history of a species complex of *Primulina eburnea* (Gesneriaceae) from limestone karsts of southern China, a biodiversity hot spot. Evol Appl.

[38] Liu H, Li B (2018). A review of research developments in *Primulina eburnea*. J Guilin Normal College.

[39] Zhang X, Yang L, Kang M. Post-pollination reproductive isolation of sympatric populations of *Primulina eburnea* and *P. mabaensis* (Gesneriaceae). Biodivers Sci. 2017;25:615–620.

[40] Feng C, Feng C, Kang M (2016). The first genetic linkage map of *Primulina eburnea* (Gesneriaceae) based on EST-derived SNP markers. J Genet.

[41] Feng C, Feng C, Yang L, Kang M, Rausher MD (2019). Genetic architecture of quantitative flower and leaf traits in a pair of sympatric sister species of *Primulina*. Heredity.

[42] Feng C, Ding D, Feng C, Kang M (2020). The identification of an R2R3-MYB transcription factor involved in regulating anthocyanin biosynthesis in *Primulina swinglei* flower. Gene.

[43] Yi H, Wang J, Wang J, Rausher M, Kang M (2022). Genomic insights into inter- and intraspecific mating system shifts in *Primulina*. Mol Ecol.

[44] Taylor NG (2008). Cellulose biosynthesis and deposition in higher plants. New Phytol.

[45] Polko JK, Kieber JJ (2019). The regulation of cellulose biosynthesis in plants. Plant Cell.

[46] Li Z, Fernie AR, Persson S (2016). Transition of primary to secondary cell wall synthesis. Sci Bull.

[47] Landrein B, Lathe R, Bringmann M, Vouillot C, Ivakov A, Boudaoud A (2021). Impaired cellulose synthase guidance leads to stem torsion and twists phyllotactic patterns in *Arabidopsis*. Curr Biol.

[48] Lei L, Li S, Du J, Bashline L, Gu Y (2013). Cellulose synthase INTERACTIVE3 regulates cellulose biosynthesis in both a microtubule-dependent and microtubule-independent manner in *Arabidopsis*. Plant Cell.

[49] Wang X, Wang H, Wang J, Sun R, Wu J, The Brassica rapa Genome Sequencing Project Consortium (2011). The genome of the mesopolyploid crop species *Brassica rapa*. Nat Genet.

[50] Schindelman G, Morikami A, Jung J, Baskin TI, Carpita NC, Derbyshire P (2001). COBRA encodes a putative GPI-anchored protein, which is polarly localized and necessary for oriented cell expansion in *Arabidopsis*. Genes Dev.

[51] Roudier F, Fernandez AG, Fujita M, Himmelspach R, Borner GH, Schindelman G (2005). COBRA, an Arabidopsis extracellular glycosyl-phosphatidyl inositol-anchored protein, specifically controls highly anisotropic expansion through its involvement in cellulose microfibril orientation. Plant Cell.

[52] Roudier F, Schindelman G, DeSalle R, Benfey PN (2002). The COBRA family of putative GPI-anchored proteins in Arabidopsis. A new fellowship in expansion. Plant Physiol.

[53] Brady S, Song S, Dhugga KS, Rafalski JA, Benfey PN (2007). Combining expression and comparative evolutionary analysis. The COBRA gene family. Plant Physiol.

[54] Brown DM, Zeef LAH, Ellis J, Goodacre R, Turner SR (2005). Identification of novel genes in Arabidopsis involved in secondary cell wall formation using expression profiling and reverse genetics. Plant Cell.

[55] Sindhu A, Langewisch T, Olek A, Multani DS, McCann MC, Vermerris W (2007). Maize brittle stalk2 encodes a COBRA-like protein expressed in early organ development but required for tissue flexibility at maturity. Plant Physiol.

[56] Liebminger E, Grass J, Altmann F, Mach L, Strasser R (2013). Characterizing the link between glycosylation state and enzymatic activity of the endo-beta 1,4-glucanase KORRIGAN1 from *Arabidopsis thaliana*. J Biol Chem.

[57] Lei L, Singh A, Bashline L, Li SD, Yingling YG, Gu Y (2015). CELLULOSE SYNTHASE INTERACTIVE1 is required for fast recycling of cellulose synthase complexes to the plasma membrane in *Arabidopsis*. Plant Cell.

[58] Szyjanowicz PMJ, McKinnon I, Taylor NG, Gardiner J, Jarvis MC, Turner SR (2004). The irregular xylem 2 mutant is an allele of korrigan that affects the secondary cell wall of *Arabidopsis thaliana*. Plant J.

[59] Wang L, Guo K, Li Y, Tu Y, Hu H, Wang B (2010). Expression profiling and integrative analysis of the *CesA/Csl* superfamily in rice. BMC Plant Biol.

[60] Cao S, Cheng H, Zhang J, Aslam M, Yan M, Hu A (2019). Genome-wide identification, expression pattern analysis and evolution of the *Ces/Csl* gene superfamily in pineapple (*Ananas comosus*). Plants.

[61] Li G, Liu X, Liang Y, Zhang Y, Cheng X, Cai Y (2020). Genome-wide characterization of the cellulose synthase gene superfamily in *Pyrus bretschneideri* and reveal its potential role in stone cell formation. Funct Integr Genomic.

[62] Kang M, Tao J, Wang J, Ren C, Qi Q (2014). Adaptive and nonadaptive genome size evolution in Karst endemic flora of China. New Phytol.

[63] Yuan W, Liu J, Takác T, Chen H, Li X, Meng J et al. Genome-Wide Identification of Banana *Csl* Gene Family and Their Different Responses to Low Temperature between Chilling-Sensitive and Tolerant Cultivars. 2021;10:122. 10.3390/plants10010122 PMC7827608 33435621

[64] Feng C, Wang J, Wu L, Kong H, Yang L, Feng C (2020). The genome of a cave plant, *Primulina huaijiensis*, provides insights into adaptation to limestone karst habitats. New Phytol.

[65] Wang Y, Wang X, Paterson AH (2012). Genome and gene duplications and gene expression divergence: a view from plants. Ann N Y Acad Sci.

[66] Blaise D, Singh JV, Bonde AN, Tekale KU, Mayee CD (2005). Effects of farmyard manure and fertilizers on yield, fibre quality and nutrient balance of rainfed cotton (*Gossypium hirsutum*). Bioresource Technol.

[67] Li W. The mechanism of nitrogen rate affecting lodging resistance in different wheat varieties and the regulation effect of irrigation and nitrogen interaction. PhD Thesis ed. Shandong Agriculture University; 2020.

[68] Tian H, Song H, Wu X, Zhang Z (2022). Responses of cell wall components to low nitrogen in rapeseed roots. Agronomy.

[69] Tang W, He X, Qian L, Wang F, Zhang Z, Sun C (2019). Comparative transcriptome analysis in Oilseed rape (*Brassica napus*) reveals distinct gene expression details between nitrate and ammonium Nutrition. Genes.

[70] Voorrips RE (2002). MapChart: software for the graphical presentation of linkage maps and QTLs. J Hered.

[71] Wang Y, Tang H, DeBarry JD, Tan X, Li J, Wang X (2012). MCScanX: a toolkit for detection and evolutionary analysis of gene synteny and collinearity. Nucl Acids Res.

[72] Chen C, Chen H, Zhang Y, Thomas HR, Frank MH, He Y (2020). TBtools: an integrative toolkit developed for interactive analyses of big biological data. Mol Plant.

[73] Edgar RC (2004). MUSCLE: multiple sequence alignment with high accuracy and high throughput. Nucl Acids Res.

[74] Nguyen LT, Schmidt HA, von Haeseler A, Minh BQ (2015). IQ-TREE: a fast and effective stochastic algorithm for estimating maximum-likelihood phylogenies. Mol Bio Evol.

[75] Feng C, Xu M, Feng C, Wettberg EJB, Kang M (2017). The complete chloroplast genome of *Primulina* and two novel strategies for development of high polymorphic loci for population genetic and phylogenetic studies. BMC Evol Biol.

[76] Kim D, Langmead B, Salzberg SL (2015). HISAT: a fast spliced aligner with low memory requirements. Nat Methods.

