# Supplementary material for: Genome-wide analysis of the cellulose toolbox of Primulina eburnea, a calcium-rich vegetable
Source: BMC Plant Biol. 2023 May 16;23:259. doi: 10.1186/s12870-023-04266-z (PMC10186795; doi:10.1186/s12870-023-04266-z)
Supplement: Supplementary file 1 — Supplementary Material 1 [file 12870_2023_4266_MOESM1_ESM.docx]

**Supplementary Texts**

**Text S1: Protein sequences for 39 potential cellulose biosynthesis-involved genes in *Primulina eburnea*.**

>Peb01198

MDTKGRLVAGSHNRNEFVVINADDVGRVTSVKELSGQNCQICGDEIEFTVDGEPFVACNECAFPVCRSCYEYERREGNQACPQCKIRYKRIKGSPRVDGDEDEDEFDDLENEFDYNIDERRVPMQFSEAGHFARHNIGRSASGITVASEADASNLNSDIPLLTYGQEDDTISADKHALIIPPFMSRGKRIHPAPFSDSSMSLPRPMDPKKDLAVYGYGTIAWKERMEEWKRKQNDKLQVVKHQGDKGGVNGDELDDPDLPKMDEGRQPLSRKLPISSSKISPYRIIIVIRMAILGLFFHYRILHPVNDAYGLWLTSIICEIWFAVSWIFDQLPKWCPIERETYLDRLSLRYEKEGKPSELAPVDIYVSTVDPMKEPPLITANTVLSILAVDYPIDKVACYVSDDGAAMLTFEALSETSEFARKWVPFCKKYSLEPRAPEWYFAQKVDYLKDKVEPTFVRERRAMKREYEEFKVRINGLVATAQKVPEDGWTMQDGTPWPGNNVRDHPGMIQVFLGQNGVRDIEGNELPRLIYVSREKRPGFEHHKKAGAMNALIRVSAVISNAPYLLNVDCDHYINNSKALREAMCFMMDPQSGKKICYVQFPQRFDGIDKHDRYSNRNVVFFDINMKGLDGIQGPIYVGTGCVFRRQALYGYDAPKKAKPPGKTCNCWPKWCCFCFGSRKKNKKKTSKDKKKNTKVREASTQIHALENIEEGIDGVDNGKSSLMPQIKFEKKFGQSPVFIASTLLEDGGVPAVASSASLLKEAIHVISCGYEDKTEWGREIGWIYGSVTEDILTGFKMHCHGWRSVYCIPKRPAFKGSAPINLSDRLHQVLRWALGSVEIFLSRHCPIWYGYGCGLKPLERFSYINSIIYPLTSIPLIAYCTLPAVCLLTGKFIVPEISNYASILFMALFMSIAATSVLEIQWGRVGIDDMWRNEQFWVIGGVSSHFFALIQGLLKVLAGVNTNFTVTSKAADDGDYSDLYLFKWTSLLVPPLTLMIINIIGVIVGISDAISNGYESWGPLFGRLFFAIWVILHLYPFLKGFMGRQNRIPTIIVVWSILLASIFSLLWVRINPFLSRDGIILEVCGLNCD*

>Peb04157

MYGRDPWGGTLEIAADSATDDDRSRNLHDYDRAALSRTLDETQQSWLLGPGEQKKNKYVDLGCIIVSRKLFVWTVGTIVAAGLLAGFVTLIVKTVPRHHHRPPPPDNYTLALNKALMFFNAQRSGKLPKHNNVSWRGNSCVNDGKSGSTTLFKDLAGGYYDAGDAIKFNFPQSFAMTMLSWSVIEYSAKYEAAGELNHVKDIIKWGTDYFLKTFNNTADTIYRIVMQVGEGDTSGESTKPNDHYCWTRPEDIDYDRPVLECSSCSDLAAEMAAALASASIVFKDNKAYSQKLVHGARTLFKFARDQRGRYSVGTEASTFYNSTSYWDEFVWGASWLYYATGNSSYLQLATAPGLAKHAGAFWGGPFYGVLSWENKLAGAQVLLSRLRLFLSPGYPYEEILSTFHNQTSIFMCSFLPEFSTFNRTKGGMIQLNHGAPQPLQYVVNAAFLATLFSDYMKAADSPGWYCGPRFYSTDTLREFAQTQIDYVLGKNPRQMSYVVGFGNHYPKHVHHRGASIPKNQAKYSCTGGWKWRDSSKANPNILVGAMVAGPDRQDGFHDVRTNYNYTEPTLAGNAGLVAALVALSGGSTMEIDKNTIFSAVPPMFPIPPPPPAPWRP*

>Peb07246

MEVRCFRSILEFKISAVLLVLLLSCFSFTPAEAYDALDPNGNITIKWDITTWNPDGYIAVVTIFNFQQYRHIQAPGWTLGWTWAKKEVIWAMMGGQTTEQGDCSRFKGSIPHCCKKDPTVVDLLPGTPYNQQIANCCKGGVITSWVQDPVNSAGSFQLSVGAAGTTNKTVRVPKNFTLKAPGPGYTCGPAKIVKPTKYITSDGRRVTQAMMTWNVTCTYSQFLSQKTPTCCVSLSSFYNNTIVPCPTCTCGCPSNLTQPGTCVDPDAPHLASVVSDRAKSNSLAPLVQCTNHMCPIRIHWHVKLNYKEYWRVKVTITNFNYRMNYTQWNLVVQHPNFDNLTQIFSFNYKPLTPYQSINDTAMLWGIKFYNDLLMQAGHAGNVQSELLFRKDKSTFSFEKGWAFPRRIYFNGDNCVMPPPDKYPYLPNAGFRKKISLFMLIITVIGSLMKTVDFSFL*

>Peb07888

MVMDTGGRLVAGSHNRNEFVLINADEIGRIKSVQELSGQKCQICGDEVEITIDGELFVACNECAFPVCRTCYEYERKEGNQTCPQCRTRYKRIKGSPRVDDDEEEEDFDDLEYEFDYGDIDVPGCTRKAHTRLASFTSRDSSSQVLEIPLLTYSEEDAQVSYDQHAIILPPFVNDGDGMHLSSPGASAHLPSRPMVREKDIALYGYGSVSWKDQMEDWKKRQSENLQMIKHQGNNDSGDFDGSELEPDLPMMDEGRQPLSRKLPISSSKINPYRLIIILRVAVLGFFFHYRILHPVPGAYGLWMTSVICEIWFSASWILDQFPKWSPVERETYLDRLSLRYEKEGNHSELADIDIFVSTVDPMKEPPLITANTVLSILAVDYPVDKVSCYVSDDGAAMLTFEALSETSEFSRKWVPFCKKFNIEPRAPEWYFSQKMDYLKNKVHPAFVRERRAMKREYEEFKVRINHLVAMAEKVPEDGWTMQDGTPWPGNSVRDHPGMIQVFLGHDGLHDVEENELPRLVYVSREKRPSFEHHKKAGAMNALIRVSAVLSNAPYILNVDCDHYINNSKALREAMCFMMDPTSGKGVCYVQFPQRFDGIDLHDRYSNHNSVFFDINMKGLDGLQGPIYVGTGCVFRRQALYGYDAPAKKKLPSKTCNCWPKWCCFCCGSRKSKRGKTKKDKMKKSKLRETSKQIHALETIEEGIEGTDITSHISHEKLEKKFGQSPVFVASTLIENGGVLKEVSSASLLEEAIHVITCGYEDKTEWGKEVGWIYGSVTEDILTGFKMHCHGWRSVYCMPKRPAFKGSAPINLSDRLHQVLRWALGSVEIFLSKHCPIWYGYGGGLKWLERISYINSVVYPWTSIPLVIYCTLPAICLLAGKFIVPEISNYASIIFISLFISIAATGSLEMQWGGVRIDDWWRNEQFWVIGGVSSHLFALFQGLLKVLAGVSTNFTVTSKGGDDGEFSELYLFKWTSLLIPPLTLLIVNIIGVVAGIANAINNGYESWGLLFGKLLFAFWVIMHLYPFLKGMMGKQEKIPTVVVIWSILLASIITLLWVRINPFLSRDGPVLEICGLNCDD*

>Peb08779

MQHAKSESDVTSLAPSSPSRSPKRHVYYVQSPSRDSHDGDKSSMQPSPMESPSHQSFGRHSRNSSASRFSGILPSSGRKGRKRNDKGWPECDVIVEEGKYDEFDDDKKFTRRCQAAMAVAGFLLLFTVFCLIIWGSARPFKAEVAVKSLSVSNFYIGSGSDSSGVPTNMLNFNGSLKLSIYNPATFYGIHVSSMPVNLFYSDFVVASGQLKKYFQPRKSRRTVLVHIEGTKIPLYGAGSTLQVGSNNAVQVPLMLNFTVQSRGDVVGKLVRTKHRKTISCPLKIDSTSNKIIEFKKNSCDYS*

>Peb09059

MAGLHTSLRPQLPSSATNHQSFAAKLLLLLTLLPLSLAVFAFLLQWRGGGVDDPISRWSPDESYKFPGMDSSPLATVGHSSSSHSSDCSTLLGHSNSASFPYFRDWKYKFDPDLKPKICITTSTSASLEQILPWMFYHKVIGVSTFFLFVEGRAASPPVSKVLESIHGVKVIYRTKDLEEQQARSRIWNESWLSSFFYKPCNYELFVKQSLNMEMAIVMARDAGMDWILHLDTDELIHPAGGREYSLRQLLLDVPSHVDMVVFPNYESSVERDDVKEPFTEVSMFKRNYDHLTKDTYFGMYKESTRGNPNYFLTYGNGKSIARVQDHLRPNGAHRWHNYMKTPSEIKFEEAAVLHYTYAKFSDLTSRRDRCGCKPTKEDVKRCFMLEFDRDVSVDYLHHHHYYFVIYLAPSIVVTMNFL*

>Peb09927

MAEARQPLWRKVPISSSLINPYRIVIVIRFIVLCFFFHFRISSPAYDAYPLWIISVICEIWFGLSWILDQFPKWLPINRETYLDRLTLRFEREGDPNQLSPVDFFVSTVDPLKEPPIITANTVLSILSVDYPVEKVSCYVSDDGASMLLFDSLSETAEFARRWVPFCKKYSVEPRAPEFYFSEKIDYLKDKVQPTFVKDRRAMKREYEEFKVRINALVAKAQKKPEEGWVMHDGTPWPGNNTRDHPGMIQVYLGSEGALDVEGKELPRLVYVSREKRPGYQHHKKAGAMNALVRVSAVLTNAPFMLNLDCDHYLNNSKAVREAMCFLMDPQIGKKLCYVQFPQRFDGIDRHDRYANRNIVFFDINMKGLDGIQGPVYVGTGCVFNRQALYGYDPAASEKRPAMTCDCWPSWCCCCCGGSRKSNTKKKGLKALLGLGGLYSKKKKMMGKQYTRKSSGQAFDLEEIEEGLEGYDELEKSSLMSQKNFEKRFGMSPVFITSTLMENGGVPEGTNPTSLIKEAIHVISCGYEEKTEWGKEIGWIYGSVTEDILTGFKMHCRGWRSVYCSPTRPAFKGSAPINLSDRLHQVLRWALGSIEIFFSRHCPLWYGYGGKLKWLERLAYINTTVYPFTSIALLAYCTLPAVCLLTGKFIVPTLNNLASIWFLALFLSIIATGVLELRWSRVSIEDWWRNEQFWVIGGVSAHLFAVFQGLLKVLAGVDTNFTVTAKAADDAEFGELYLFKWTTLLIPPTTLIILNMVGVVAGIADAINNGYGSWGPLFGKLFFAFWVIVHLYPFLKGLMGRQNRTPTIVVLWSILLASIFSLVWVRIDPFLPKQTGPILKQCGVEC*

>Peb09967

MKSSNSTSGLFAGSHSRNELHCMELIKIRESATKTCRVCGDEIGLKENGERFVACGECGFPVCRPCYEYERSEGNKSCPQCHTRYKRHKGCPRVEGDDEENFDDDFEDEFRLKNHHANHVSHHHDSVNSTN*

>Peb10564

MATGRLVAGSHNRNEFVLINADEIGKVTSVKELTGQKCQICGDEIEFTVDGEPFVACNECAFPVCRPCYEYERREGNQACPQCRTRYKRIKGSPRVDGDEDEDEFDDLDNEFERKDPSQFSGSGIYGRNNICRSASGITDSSDTHPSHVISEIPLLTYGQEDDTISADKHALIIPSFMGHERRVHPTPFTDSSMSLQPRPMDPKKDLAVYGYGTIAWKERMEEWKKKQNDRLQMVEHQGDDPDLPKMDEGRQPLSRKLPISSSKISPYRIVIVLRMAILGLFFHYRIRHPVNDAYGLWLTSVICEIWFAVSWIFDQFPKWFPIERETYLDRLSLRYEKEGKPSELSSVDIFVSTVDPSKEPPLITANTVLSILSVDYPIDKVTCYVSDDGAAMLTFEALSETSEFARKWVPFCKKFSIEPRAPEWYFTQKIDYLRDKVEPTFVRERRAMKREYEEFKVRINGLVAMAQKVPEDGWTMQDGTPWPGNYVRDHPGMIQVFLGQNGIRDIEGNELPRLVYVSREKRPGFDHHKKAGAMNALIRVSAVISNAPYLLNVDCDHYINNSKALREAMCFMMDPQAGKKICYVQFPQRFDGIDRHDRYSNRNVVFFDINMKGLDGIQGPIYVGTGCVFRRQALYGYDAPKKAKPPGKTCNCWPKWCCCCCGSRKKNKKGKSKDNKKNMKSREGSSQIHALENIEEGVKGIDGEKSTLMPQVKLEKKFGQSPVFIASTLLEEGGIPSEATSASLLKESIHVISCGYEDKTEWGREVGWIYGSVTEDILTGFKMHCHGWRSVYCIPKRPAFKGSAPINLSDRLHQVLRWALGSVEILLSRHCPIWYGYGCGLKPLERFSYINSVVYPLTSIPLIAYCTLPAVCLLTGKFIVPEISNYASIIFMALFLSIAVTSVLEMQWGRVAIDDLWRNEQFWVIGGVSSHFFALIQGLLKVLAGVNTNFTVTSKAADDGDFSELYLFKWTSLLIPPLTLMIINIIGVIVGISDAISNGYESWGPLFGRLFFAIWVILHLYPFLKGFMGKQDRVPTIIVNRKDGMSRCSSTYFRKVINKLEPKLNERQRARIIGTPFGNWLKMPKLSIYSTRVDVILRSFNIDSLSFIFGKGIVIPFTSFEFSIVIGLPHGGQPVSSKMKDAETTYKIKRLEKLVEDQFNEIQMLKQMCCQSHGIVTKNVVGGRVDCEVNVGKKDDNNVSFEDFDIDLGGYNGFHDVDVVQNVIKGNGVGLTEVDVAANKVHTEKSSREIIDESNDVNCVSSSQKDTVCNVISSIVKNVLTRNNRVKKRKSDMFVTPPSSTPRRKTKAL*

>Peb12177

MDTKGRLVAGSHNRNEFVVINADDVGRVTSVKELSGQNCQICGDEIEFTVDGEPFVACNECAFPVCRSCYEYERREGNQACPQCKIRYKRIKGSPRVDGDEDEDEFDDLENEFDYISDERRAPLHFSEAGHFARHNIGRSASGITIPSEVDHSNLNSDIPLLTYGQEDDTISADKHALIIPPFMSRGKRIHPDPFTDSSMTLPPRPMDPKKDLAVYGYGTIAWKDRMEEWKRKQNGKLQVVKHQGDEGGVDGDELDDPDLPKMDEGRQPLSRKLPISSSKISPYRIIIVIRLAILGLFFHYRILHPVNDAYGLWLTSIICEIWFAVSWIFDQLPKWYPIERETYLDRLSLRYEKEGKPSELAPVDIYVSTVDPMKEPPLITANTVLSILAVDYPIDKVACYVSDDGAAMLTFEALSETSEFARKWVPFCKKYSIEPRAPEWYFVQKVDYLKDKVEPTFVRERRAMKREYEEFKVRINGLVATAQKVPEDGWTMQDGTPWPGNNVRDHPGMIQVFLGQNGVRDIEGNELPRLIYVSREKRPGFEHHKKAGAMNSLIRVSAVISNAPYLLNVDCDHYINNSKALREAMCFMMDPQSGKKICYVQFPQRFDGIDKHDRYSNRNVVFFDINMKGLDGIQGPIYVGTGCVFRRQALYGYDAPKKAKPPGKTCNCWPKWFCCCFGSRKMNKKKTSKDKKKNTKVREASTQIHALENIEEGIEGVDSEKSALMPQIKFEKKFGQSPVFIASTLLEDGGVPAGASSASLLKEAIHVISCGYEDKTEWGRDIGWIYGSVTEDILTGFKMHCHGWRSVYCIPKRPAFKGSAPINLSDRLHQVLRWALGSVEIFLSRHCPIWYGYGCGLKPLERFSYINSIIYPLTSIPLIAYCTLPAVCLLTGKFIVPEISNYASILFMALFISIAATSILEIQWGRVGIDDMWRNEQFWVIGGVSAHFFALIQGLLKVLAGVNTNFTVTSKAADDGEFSDLYLFKWTSLLIPPLTLMIINIIGVIVGISDAISNGYESWGPLFGRLFFAIWVILHLYPFLKGFMGRQNRIPTIIVVWSILLASIFSLLWVRINPFLSRDGIILEVCGLNCD*

>Peb12918

MMREGSAANDAVETLIKILSSTKEETQAKSALALSRIFDLRKDLRESSIAVKTLCSIIELLNVESENILVESCRCLAAIFLSIKVNQDVAAIARNALPLLLVLATSSVLQVQEQAVCALANLLLDGEASEKVMLEEIIVPATKVLHKGTNVGKIHAAAAIARFLHSREINSALIDCINCAGTVLALVSFLETSDSGSIVASEALDALSFLSRCVGGIGHVKPPWVVLDEYPSSITPIVSCIADATPMLQDQAIEVLSRLCRSQPLVLGNTVAGATGCISSIARRIISNSNAVVKIGGAALLVCTAKVNHQRVVEDLNSSSSWTSLVHSLVGMLSSSEFSQFNEDTISVCRIAEEEATSQGSKRSTLVINGTDIAIWLLSVFAAYDGKSKVEILEAGAIEVLTEKISQSFLLYTQAEFKEDGSIWICALLLAILFQDRDIIRANATMKAIPALARLLRSEDSAGRYFAAQAVASLVCNGSRGTLLSVANSGAAAGLISLLGCADDFINNLLELADEFGLVHYPDQVALERLFRVNDIRVGATSRKSIPALVDLLKPIPDRPGAPFLALGLLIQLARDCHPNQIVMVESGALEGLTKYLSLGPKDAYEEAATDLLGILFSTADIRKHESALAAVSQLIAVLRLGGRAARYSAAKALENLFSADHVRSAESSRQAVQPLVEILSTGSEKEQHAAIAALVTLLSENPSRALVVADVEMDAVDVLCRILSSDTSLVLKRDAAELCFVLFGNTRIRSTVAAERCIEPLVSLLVYEYSPAHQSVVLALDRLLEDEQLAELIAARGAVIPLAGLFHGQNYLLHEATCRALVKLGKDRPACKIDMVKAEVIETVLSILHEAPDFLCTAFAELLRILTNNAAIAEGPSAAKLVEPFFLLLTRSEFGTDGQRSAIRVLLNILEHQQCRANYTLASEQVIAPLLPLLDSPASAIQELAAELLSRLFLEEHLQGDPLTQQAIGPLIHILGPGTPILQQRAVRALVRVAGTWPNEIAKEGGVSELSKVILQPDSSLPQVLWESAASVLSSILQFSSDYYLEVPIAVLVRLLHSVSEGTVIGALNALLVLDSDDSTSAEAMAESGAIEALLVLLRRHQCEETAARLLEVLLNNVKIRESKATKSAILPLSQYLLDPQTQGQQARLLATLALGDLFQNESLARLADAVSACRALVNLLEDQPTEEMKVVAICALQNLVMYSRLNKRAVAEAGGVQVVLDLIGSSIPETSIQAAMFIKLLFCNNTIQEYASSETVRGLTAVIEKDLWATGTVNEEYLKALNALFGNFPRLRATEPATLSIPHLVTSLKTGSEATQEAALDALFLLRQAWSACPAEVSRAQSIASADAIPLLQYLIQSGPPRFQEKAEFLLQCLPGTLVVIIKRGNNMRQSVGVPSVYCKVTLGNNPPQLTKVVSTGPNPEWEESFAWSFESPPKGLKLHISCKNKSKMGKSKFGKVTIQVDRVVMLGAVAGEYTLLPASKSGPPRNLEIEFQWSSK*

>Peb12947

MKLNSRDRSSMEDPDGTLASVAKCIEQLRQNSSSHEKENSLRQLLELISIREDAFSAVGSHSQAVPVLVSLLRSGSLGIKIHAATVLGSLCEENELRVKVLLGGCIPPLLGLLKSNSAEGRIAAAKTIYAVSQGGVKDHVGSKIFSTEGVVPTLWEQLGKGLNNGNLVDDLLAGALRNLSSCIEGFWPASIQAGGVDILIKLLKTGESSTQANVCFLLACMMMEDASVCSEVLATETTKVLLKLLGAGNEDCARAEAARALKSLSEQCKESRREIASSNGIPILINATIAPSKEFMQGEFAQALQENSMCALANISGGLSYVISSLGKSLESCISPAQVADTLGALASALMIYNTDAEYSRASDPMEVEKTLVQQFKTRMPFLVQERTIEALATLYGNAVLASKLAASDAKRLLVGLITMATNEIQEELIKSLLILCYKEGSLWHALQGREGIQLLISLLGLSSGAAARVCCCFTLPSIQRK*

>Peb13255

MSMHGKTDSEVTSLAASSPNLAVYYVQSPSRDSHDGEKTTNSFHSTPILSPMGSPGRHSRDSSSTRYSGSLKPGSQKSSNGSRWNHRRKPERDFDAIEEEGLLDEEGGRRGGVSRRCYMVAFVVGFFVLFSFFALILWGASRNQKPVVTMKSILFDDFMIHAGSDSAVVGTEMVTMNSTLKLIFRNTGTFFGVHVSPTPLDLFFSELSLASGAINGFYQSRKSQRTIAVILMGNHIPLYGGGSNLSSKEGRPAAPVTLALNFTVRARAYVLGRLVKPKFHKIVHCSVVLDQNKMNVAIPLKNSCTFD*

>Peb13264

MGLYPRSLRHPKILLFLVLSCFCFTSTEAYDSLDPNGNITIKWDVISWTPDGYVAVVTMFNFQQYRHIQAPGWTLGWTWAKKEVIWSMMGGQATEQGDCSKYKGNVPHCCKKDPTIVDLLPGTPYNQQIANCCKGGVINSWVQDPTNAASSFQVSVGAAGTTNKTVRVPKNFTLKAPGPGYTCGPAKIVKPTKYVTSDGRRVTQAMMTWNVTCTYSQFLAQKTPTCCVSLSSFYNDTIVPCPTCTCGCQNNITQPGSCVDPESPYLASVVSDRAKTNAYAPLVQCTSHMCPIRIHWHVKLNYKDYWRVKVTITNFNYRMNYTLWNLVVQHPNFDNLTQIFSFNYKSITPYQSINDTAMLWGIKFYNDFLMQAGPLGNAQSELLFRKDMSTFSFDKGWAFPRRVYFNGDNCVMPPPDEYPYLPSTSLRQNTPFIMLIITLIASVTFLFERN*

>Peb14384

MEASAGLVAGSHNRNELVVIHGHEEHKTQKNLNGQVCEICGDAVGLTMDGELFVACNECGFPVCRPCYEYERREGNQLCPQCKTRYKRLKGSPRVEGDDDEEDIDDIEHEFNIDEQKTNTDIAEAMLHGKMSHGRGPEDEENAQYPAVVAGGRSRHVSGEFPISSHAYSGDQMMGSSLHRRVHPYPVSDETGARWDDKKLEMGWKERMEDWKMQQGNLGPEYDDSADPEMAILDEARQPLSRKVPIASSKINPYRMVIVTRLVVLAFFLRYRILNPVHDALGLWLTSIICEIWFAFSWILDQFPKWFPIDRETYLDRLSLRYEREGEPNLLSPVDIFVSTVDPMKEPPLVTANTVLSILAMDYPVDKISCYISDDGASICTFEALSETAEFARKWAPFCKKFSIEPRAPEMYFSEKVDYLKDKVQPTFVQERRAMKREYEEFKVRINALVAKATKVPPGGWIMQDGTPWPGNNTKDHPGMIQVFLGQNGGLDVEGRELPRLVYVSREKRPSFQHHKKAGAMNALIRVSGVLTNAPFMLNLDCDHYLNNSKAVREAMCFLMDPQVGKKVCYVQFPQRFDGIDKHDRYANRNTVFFDINMKGLDGIQGPVYVGTGCVFRRQALYGYEPPKGPKRPKMVSCDCCPCFGRHKKLPKYSNNDLNGGANIQGGVPPSSSPAALLKEAIHVISCGYEDKTEWGSELGWIYGSITEDILTGFKMHCRGWRSIYCMPKRAAFKGSAPINLSDRLNQVLRWALGSVEIFFSRHSPLWYGYKEGNLKWLERFAYVNTTVYPFTSLPLLAYCTLPAICLLTGKFIMPEISTFASLFFIALFLSIFVTGILELRWSGVSIEEWWRNEQFWVIGGVSAHLFAVIQGLLKVLAGIDTNFTVTSKATDDEDFGELYAFKWTTLLIPPTTILIINLVGVVAGISDAINNGYQSWGPLFGKLFFAFWVIVHLYPFLKGLMGRQNRTPTIVIIWSVLLASIFSLLWVRIDPFILKTMGPNTKQCGINC*

>Peb14536

MSMYGRDPWGGSLEIAADSATDDDRSRNLHDFDRAALSRPLDETQQSWLLGPGEQKKKRYVDLGCIIVSRKIFLWTVGVILGVGLLAGFISLILKTVPRHHHRPPPPDNYTLALQKALMFFNAQRSGKLPKHNNVSWRGNSCVNDGKSDSSTIFKDLAGGYYDAGDAIKFNFPQSFAMTMLSWSVIEYSAKYEAAGELNHVKEIIKWGTDYFLKTFNSTADTIDRIVAQVGQGDTSGGPTPNDHYCWMRPEDIDYDRPITECHSCSDLAAEMAAALASASIVFKDNKAYSQKLVHGAKALYKFSRDQRGRYSAGNEAATFYNSTSYWDEFVWGAAWMYYATGNASYLQLATTPGLAKHAGAFWGGPYYGVLNWDNKLPGAQVLLSRLRLFLSPGYPYEEILKTFHNQTSIFMCSFLPYFTTFNRTRGGMIQLNHGAPQPLQYVVNAAFLATLFSDYMRAADTPGWYCGPHFYSTDTLREFAQTQMDYILGRNPQKMSYVVGFGNHYPKHVHHRGASIPKSKIKYNCKGGWKWRDSKKPNPNTVIGAMVAGP*

>Peb14643

MEANAGMVAGSHKRNELVRIRHDSDSGPKPLKNLNGQICQICGDTVGLTANGDVFVACNECAFPVCRACYEYERKDGNQSCPQCKTRYKRHKGSPRVDGDDDDEDDDDLENEFLYSQGKNKDRSQWQGDDADLSASSRREAHQPIRLLTNGQPVSGEIPPSMLDTQSVRSTSGPLGPGDRVHSLPYVDPQQPVPVRIVDPSKDLNSYGLGNVDWKERVEGWKVKQDKNMGQMANRYIEGKGDIEGTGSNGEELQMADDARQPMSRIVPISSSHLTPYRVVIILRLIILGFFLQYRCTHPVKDAYPLWLTSVICEVWFAFSWLLDQFPKWYPINRETYLERLAVRYDREGEPSQLAPIDIFVSTVDPMKEPPLITANTVLSILSVDYPVDKVSCYVSDDGAAMLTFEALSETAEFARKWVPFCKKHNIEPRAPEFYFAQKIDYLKDKIQPSFVKERRAMKREYEEFKVRINALVAKAQKMPEEGWTMQDGTSWPGNNTRDHPGMIQVFLGHSGGLDTDGNELPRLVYVSREKRPGFQHHKKAGAMNALIRVSAVLTNGAYLLNVDCDHYFNNSKALKEAMCFMMDPAYGKKTCYVQFPQRFDGIDLHDRYANRNIVFFDINLKGLDGLQGPVYVGTGCCFNRQALYGYDPVLTEEDLRPNIIVRSCCGSRKKGRRANKKYTDKNRAVKRTESTIPIFNVEDIEDGVEVAKHSLSWTGYDDEKSLLMSQKSLEKRFGQSPVFIAATFMEQGGIPPSTNPATLLKEAIHVISCGYEDKTEWGKEIGWIYGSVTEDILTGFKMHARGWISIYCMPPRPAFKGSAPINLSDRLNQVLRWALGSIEILLSRHCPIWYGYTGKLQLLERLAYINTIVYPLTSIPLIAYCILPAICLLTNKFIIPEISNFASMWFILLFVSIFATGILEMRWGGVTVEDWWRNEQFWVIGGTSAHLFAVFQGLLKVLAGIDTNFTVTSKASDEDGDFSELYVFKWTSLLIPPTTVLIVNLVGIVAGVSYAINSGYQSWGPLFGKLFFAMWVVVHLYPFLKGLLGRQNRTPTIVIVWSILLASIFSLLWVRIDPFTSEATKRAAQDREVREKGHVGNLLKLCCAISM*

>Peb15047

MEASAGMVAGSHKRNELVRIRHDSDSGAKPLKNLNGQICQICGDTVGLTANGDVFVACNECGFPVCRACYEYERKDGNQSCPQCKTRYKRLKGSPRVDGDDDEDEDEDDLENEFLYSQGKNKARSQWQVDDADLSASSRREAHQPIRLLTNGQPVSGEIPPSMLDTQSVRSTSGPLGPGDRVHSLPYVDPQQPVPVRIVDPSKDLNSYGLGNVDWKERVEGWKLKQDKNMGQMANRYSEGKGDTEGTGSNGEELQMADDARQPMSRIVPISSSHLTPYRVIIILRLIILGFFLQYRCTHPVKDAYPLWLTSVICEVWFAFSWLLDQFPKWYPINRETYLERLAVRYDREGEPSQLAPIDIFVSTVDPMKEPPLITANTVLSILCVDYPVDKVSCYVSDDGASMLTFEALSETAEFARKWVPFCKKHNIEPRAPEFYFAQKIDYLKDKIQPSFVKERRSMKREYEEFKVRINALVAKAQKMPEEGWTMQDGTSWPGNNTRDHPGMIQVFLGHSGGLDTDGNELPRLVYVSREKRPGFQHHKKAGAMNALIRVSAVLTNGAYLLNVDCDHYFNNSKALKEAMCFMMDPAYGKKTCYVQFPQRFDGIDLHDRYANRNIVFFDINLKGLDGLQGPVYVGTGCCFNRQALYGYDPVLTEEDLQPNIIVRSCCGSRKKGKRANKKYIDKNRAVKRTESTIPIFNVEDMEDGVEGYDDEKSLLMSQKSLEKRFGQSPVFIAATFMEQGGIPPSTNPATLLKEAIHVISCGYEDKTEWGKEIGWIYGSVTEDILTGFKMHARGWISIYCMPPRPAFKGSAPINLSDRLNQVLRWALGSIEILLSRHCPIWYGYTGKLQLLERLAYINTIVYPLTSIPLIAYCILPAICLLTNKFIIPEISNFASMWFILLFVSIFATGILEMRWGGVTVEDWWRNEQFWVIGGTSAHLFAVFQGLLKVLAGIDTNFTVTSKASDEDGDFSELYVFKWTSLIIPPTTVLIVNLVGIVAGVSYAINSGYQSWGPLFGKLFFAIWVIVHLYPFLKGLLGRQNRTPTIVIVWSILLASIFSLLWVRIDPFTSEATKRAAQGQCGINC*

>Peb15092

MDTKGRLVAGSHNRNEFVLINADEIGRVTSVKELTEQICQICGDEIEFGVDGEPFVACNECAFPVCRPCYEYERREGNQTCPQCKTRYKRIKGSPRVDGDEEEEEFDDLENEFDYNSNERRDPHQIAEAELAARLNVGRGASVIYTASSELDPSAANADIPLLTYGQEDDTISADKHALIIPPFMGRGKRVHPLPFSDSSMTVPPRPMDPKKDLAVYGYGTVAWKERMDEWKKKQNDRLQVVKHQGDKLGDELDDPDLPKMDEGRQPLSRKLPIPSSKINPYRMIILLRMAILGLFFHYRILHPVNDAYGLWLTSIICEIWFAVSWLFDQFPKWSPILRETYLDRLSLRYEKEGKPSELMPVDIFVSTVDPMKEPPLITANTVLSILAVDYPVDKVACYVSDDGAAMLTFEALSETCEFARKWVPFCKRFSIEPRAPEWYFAQKVDYLKDKVEPTFVRERRAMKREYEEFKVRINGLVATAQKVPEDGWTMQDGTLWPGNNVRDHPGMIQVFLGQNGVQDIEGNELPRLIYVSREKRPGFEHHKKAGAMNALIRVSAVISNAPYLLNVDCDHYINNSKALREAMCFMMDPQAGKKICYVQFPQRFDGIDRHDRYSNRNVVFFDINMKGLDGIQGPIYVGTGCVFRRQALYGYDAPKKAKPSGKTCNCWPKWCCFCCGSRKKSKKGKSKNNKKKTKSREASTQVHALENIEEGIEVIESEKPTFMPQIKFEKKFGQSPVFIFSTLLEEGGVPPGTSSASLLKEAIHVISCGYEDKTDWGKEVGWIYGSVTEDILTGFKMHCHGWRSVYCIPHRPAFKGSAPINLSDRLHQVLRWALGSVEIFFSRHCPLWYGYGCGLKPLERFSYINSVVYPLTSLPLIVYCTLPAVCLLTGKFIVPEISNYASIIFMGLFISIAATSVLEMQWGGVGIDDLWRNEQFWVIGGVSSHFFALIQGLLKVLAGVSTNFTVTSKAADDGAFSELYLFKWTSLLIPPMTLMIINIIGVIVGISDAINNGYESWGPLFGRLFFALWVIVHLYPFLKGFMGKQDRIPTIIVVWSILLASILTLLWVRINPFLSRGGIVLEVCGLNCD*

>Peb15480

MTHCTPAPNQKVSLNYTVTITLSLSAALQVKSPAELPYIGQNKMHAKTDSEVTSSAPSSPDHHRRPVYYVQSPSRDSHDGEKTMTSFHSTPVMGSPVGSPPHSHSSVGRHSRESSTSRFSGSLKPGSRKISPNDVGSGARGQRKGQKNWKEFDMIEEEGLLEDEERRKGLPRRCYVLAFVVGFFVLFSFFALILLAASKPQKPRIAMKSITFERFVVQAGSDDSGVATDMISLNSTVAFTFRNKATFFGVHVTSTPLTLSYSQLTIGSADMKQFYQRRKTQRNVVVSVIGDKIPLYGNGASLSTPTGTTTLPVPLKLDFTVRSRAYILGKLVKPKFYKKIECKIVLDPKKLNAPISLKNSCTYD*

>Peb17144

MEASAGLVAGSHNRNELVVIHGHEEPKTLKNLNGQVCEICGDAAGLTVDGELFVACNECGFPVCRPCYEYERREGNQLCPQCKTRYKRLKGSPRVEGDDDEEDIDDIEHEFNIDEQKKNTDIAESMLHGKMSYGRGQEDEENAQYPAVVAGGRSHHVSGEFPTPSHAYGGDQMMGSSLHRRVHPHPVSDETGGARWDDKKLEMGWKERMEDWKMQQGNLGPEYDDSADPEMTILDEARQPLSRKVPIASSKINPYRMVIVTRLVVLAFFLRYRILNPVHDAFGLWLTSIICEIWFAFSWILDQFPKWFPIDRETYLDRLSLRYEREGEPNMLAPVDIFVSTVDPMKEPPLVTANTVLSILAMDYPVDKISCYISDDGASMCTFEALSETAEFARKWVPFCKKFSIEPRAPEMYFSEKVDYLKGKVQPTFVQERRAMKREYEEFKVRLNALVAKAIKVPPGGWIMQDGTPWPGNNTKDHPGMIQVFLGQNGGLDVEGRELPRLVYVSREKRPSFQHHKKAGAMNSLIRVSGVLTNAAFMLNLDCDHYLNNSKAVREAMCFLMDPQVGKKVCYVQFPQRFDGIDKHDRYANRNTVFFDINMKGLDGIQGPVYVGTGCVFRRQALYGYEPPKGPKRPKMVSCDCCPCFERRKKLPKYSKNDPDGEANIQGFDEDNKILMSQMNFEKKFGQSPIFVTSTLMIEGGVPPSSSPAALLKEAIHVISCGYEDKTEWGSELGWIYGSITEDILTGFKMHCRGWRSIYCMPKRAAFKGSAPINLSDRLNQVLRWALGSVEIFFSRHSPLWYGYKEGNLKWLERFAYVNTTVYPFTSLPLLAYCTLPAICLLTGKFIMPEISTFASLFFIALFLSIFVTGILELRWSGVSIEEWWRNEQFWVIGGVSAHLFAVIQGLLKVLAGIDTNFTVTSKATDDEDFGELYAFKWTTLLIPPTTILIINLVGVVAGISDAINNGYQSWGPLFGKLFFAFWVIVHLYPFLKGLMGRQNRTPTIVIIWSVLLASIFSLLWVRIDPFILKTKGPNTKQCGINC*

>Peb17365

MYQNLRSNLTLKCRPAGLSTTHRNTTAVSSPAFLAFRRPIGFFTLLLLISLALLAFLLQFTDPTVRLQHGNDGPAAFAGLAFSEPVSSSPSSVDCAGVLGMSDKVSFPYYREWKFGLGSDLGPKVIGVAKFFLFVEGEAASPAVYKVLDSIPGVRVVRRTRELDEQQAKSRIWNETWLSSFFYKPCNYELFVKQTLNMEMGIVMATEAGMDWIIHVDTDELIHPVGSSQYSVQKLLLDVPQDVDLVIFPNYESIVERDDIKEPFSEVSMFKKNHDHVPRDVYFGNYMEASHGNPNYFLTYGNGKSAARLQHHLRPNGAHRWHNYNKIPKEISFNEAAVLHYTYAKFSDLTSRRDRCGCKLTEEDIERCFMLDFDRAAFVIASTATEEEMLRWYRAHVVWDDKELNLKLLKEGVLTRIHTPMVLIQSLRESQLFSSVITASMSSGSTYPSTINTENNTYLRAINDQTRSRNVLQFPAIPPQAPPTYGIHLDT*

>Peb17602

MEPEVDTKGKSMKNTGGQVCQICGDNVGLNAAGEPFVACNVCAFPVCRPCYEYERKDGNQSCPQCKTRYKRHKGSPAISGDGEEDTAVDGNDNDVHYSDGKNEKQKISDRMLSWHTNYGRGEDIGIPKYDKEVPHNRIHLLTNGTDVSGELSAASPERVSMASPPPSGVGKPRIVDPVREFGSQGLGNVAWKERVDGWKMKQEKPVVPMTTSHPPSERGGDIDASTDILVDDSLLNDEARQPLSRKVSVPSSRINPYRMVIVLRLIILCIFLHYRITNPVPNAFALWLISVICEIWFAISWILDQFPKWLPVNRETYLDRLSLRYDREGEPSQLAAVDIFVSTVDPLKEPPLVTANTVLSILAVDYPVDKVSCYVSDDGSAMLTFEALSETSEFARKWVPFCKKFNIEPRAPEWYFAQKIDYLKDKVQPSFVKERRAMKREYEEFKIRVNGLVAKAQKVPDEGWIMQDGTPWPGNNTRDHPGMIQVFLGQSGGLDSEGNELPRLVYVSREKRPGFQHHKKAGAMNALVRVSAVLTNGPFLLNLDCDHYINNSKALRESMCFLMDPNLGKYVCYVQFPQRFDGIDKSDRYANRNTVFFDINLRGLDGVQGPVYVGTGCVFNRTALYGYEPPHKPKQKKSGVFSSCFGRSGKNTSKSSKMGSDKKKSGKHVDPTVPIFSLEDIEEGVEGAGFDDEKSLLMSQMSLEKRFGQSSVFVASTLMENGGVPQSATPETLLKEAIHVISCGYEDKTEWGSEIGWIYGSVTEDILTGFKMHARGWRSIYCMPPRPAFKGSAPINLSDRLNQVLRWALGSVEILFSRHCPIWYGYKGRLKWLERFAYVNTTIYPITSIPLVFYCTLPAVCLLTGKFIIPQISNIASIWFLSLFLSIFATGILEMRWSGVGIDEWWRNEQFWVIGGVSAHLFAVFQGLLKVLAGIDTNFTVTSKASDEDGDFTELYMFKWTTLLIPPTTLLIINLVGVVAGISYAINSGYQSWGPLFGKLFFAFWVIVHLYPFLKGLMGRQNRTPTIVVVWSILLASIFSLLWVRIDPFTTRVTGPDVEECGINC*

>Peb17720

MELHCRSLKHPTVLLFFLLSCFCFTSTEAYDSLDPNGNITIKWDVISWTPDGYVAVVTMFNFQQYRHIQAPGWTLGWTWAKKEVIWSMMGGQATEQGDCSKYKGNVPHCCKKDPTIVDLLPGTPYNQQIANCCKGGVINSWVQDPTNAASSFQVSVGSAGTTNKTVRVPKNFTLKAPGPGYTCGPAKIVKPTKYVTPDGRRVTQAMMTWNVTCTYSQFLAQKTPTCCVSLSSFYNDTIVPCPTCTCGCQNNITQPGSCVDPESPYLASVVSDRAKVNAYAPLVQCTSHMCPIRIHWHVKLNYKDYWRVKVTITNFNYRMNYTLWNLVVQHPNFDNLTQIFSFNYKPLTPYQTINDTAMLWGIKFYNDLLMQAGPLGNAQSELLFRKDKSTFSFEKGWAFPRRVYFNGDNCVMPPPDEYPYLPSSSLRQNTPFLMLIITLIASVTFLCN*

>Peb18699

MMGNGGFAAMLVTLMVVCCVCTVRAEDEIIVKKVGGKRVCKQGWECDTFSEYCCNQTISKVFQTYQFENLFSKRNSPVAHAVGFWDYRSFILASSVYQPLGFGTTGGKLMQMKELAAFLGHVGAKTTCGYGVATGGPLAWGLCYNKEMSPSQDYCDDFFKYEYPCAPGAQYYGRGAIPVYWNYNYGKLGKHMKIDLLNHPEYLEQNATIAFMAAMSMWMTSKRKGQPSAHDVFVGNWKPTKNDTMEKRVPGFGATMNILYGDLVCGQGEIDPMNVCISHYQEYLDLMGVGREKAGPHEVLSCGEQIVFNPSYKSSS*

>Peb20914

MDDSDGTLATVAQCIEQLRQKSSSPQEKEDSLGELLELITTRENAFSAVGSHSQAVPVLVSLLRSGSLEIKIQAATVLGSLCQENELRVKVLLGGCIPPLLGLLKSNSAEGQIAAAKTVYAVSQGDAKDHVGSKIFSTEGVVPVLWGQLEKGLKVGNVVDDLLTGALRNLSSSTEGFWPATIQSGGVDVLVKLLTTGQSSTQANVCFLLACMMMEDASVCSKVIAAEATKLLLKLLGPGNEASVRAEAAAALKSLSAQCKEAKREVANANGIPVLINATIAPSKEFMQGEFAQALQENAMCALANISGGLSYVISSLGQSLESCSSAAQVADTLGALASALMIYDSKAECTRASDPLEVETILVQQFKPQLPFLIQERIIEALASLYGNAILSSKLLNSDAKRLLVGLITMAANDVQEELIKSLLILCNNEGSLWHALQGRDGIQLLISLLGLSSEQQQECAVALLCLLSNENDESKWAITAAGGIPPLVQILETGSAKAKEDSATILGNLCNHSEDIRACVESADAVSALLWLLKNGSSNGKEIAAKTLNHLIHKSDTATISQLSALLTSDLPESKVYVLDALKSLLSMAPLNEMLREGSAANDAIETMLKILSSPREETQANSARALSGIYDFRKDLRQSSIAVKTLWSVMKLLNAESENVLVESSRCLAAIFLSIKDNRDVATAAREALPLLVVHANSSVLRVAEQAVCALSNLLKDSEASEKALLEEVILPATRVLREGTNIGKTHAAAAIARFLHSRQIDDNLIECVNRTGTVLGLVSFLEAADIESVTTSEALDALAFLSRSAGVVGHMKPAWAVLAEHPSSITPIVACIADVPPLLQDKAIEILSRLCRAQPLFLGSTIACSTGCISSIVRRVIDSSISGVKIGGAALLVCTAKVNHQRVVEDFNGSNLCDSLISSLVEMVESTESSVGDQGTKDIISISRITAEVESKDESERSTSVISGSNISVWLLSILACYDDKSKLKIMESGALEVLTEKISQFFSQYSQADFKEDDSIWICALLLAVLFQDRDVIRSNATMKTIPVLANMLRSEEPANRYFAAQAVASLVCNGSRGTLLSVANSGVAAGLISFLGCADVDIYDLLELSEEFALIRYPDQVALERLFRIDDIRVGATSRKAIPALVDLLKPIPDRPGAPFLALGILIQLAKDCTPNQIVMVESGALEGLTKYLSLGPQDTYEAAAADLLGILFSTAEIRRHESAFGAVSQLVAVLRLGGRAARYSAAKALENLFSADHVRNADSARHAVRPLVEILNTGLEKEQHAAIAALVRLLRENPSKALAVADVEMNAVDVLCRILSSNYSMQLKGDAAELCCVLFCNTRIRSTVAAARCVEPLVSLLVTEYSPAHLSVVHALDKLLDDEQLAELVAAHGAVIPLVSLLYGQNFSLHEAVSRALVKLGKDRPASKMEMMKAGVIESLLDILHEAPDFLCAAFVELLRILTNNATIAKGPSASKVVEPLFQLLTRTEFGPDGQHSALQVLVNILEHPQCRADYTLTGQQALEPLLPLLDSPASAVQQLAAELLSHLLLEEHLQRDPLTQQVIGPLVRILGSGVPVLQQRAVRALVSVAVTWPNEIAKEGGVAELSKVILQADPFLPHALWESAASVLSIILQFSSEFYLEVPVAVLVRLLRSGLESTIIGALNALLVLESDDSTSAQAMAESGAVEALLDLLSSHQCEETAARLLEVLLNNVKIRESKVTKSAILPLSQYLLDPQTQGQQARLLATLALGDLFQNEALARTADAVSACRALVNILEDQPTEEMKVVAMCALQNLVMYSRSNKRAVAEAGGVQVVLDLIGSSDPETSVQAALFIKLLFSNNTIQEYASSETVRAITAAIEKDLWATGAVNDEYLKALNALFGNFPRLRATEPATLSIPHLVASLKTGSEATQEASLDALFLLRQAWSACPAEVSRAQSIAAADGIPLLQYLIQSGPPRFQEKAEFLLQCLPGTLTVIIKRGNNMRQSVGSPSVYCKLTLGNTAPRQTKVVSTGPNPEWDETFAWSFESPPKGQKLHISCKNKSKMGKSSFGKVTIQIDRVVMLGAVSGEYTLLPESKSGPSRNLEIEFQWSNK*

>Peb20975

MVTVHVVSHLPPNSEKLFVHCASSDDDLGNIALYPNKEFHFSFCVIPFSTLFSCDLQWGLYYKSFDVFNSEQSGGPCSQGKCIWSAVNDGPKEKKKKKYVDLGCIVCSRTVLKWTLLSFLIAFVVIGLPIIIAKSLPKHKAKPQPPDNYTVALHKALLFFNAQKSGKLPKNNGIPWRRDSGLQDGSDATDVKGGLVGGYYDAGDNTKFHFPMSFAMTMLSWSVIEYEHKYRAIGEYDHVTDLIKWGTDYLLLTFNSSATKIDKIYGQVGGSQNGSQTPDDHYCWERPEDMDYPRKSISISSGPEVAGEMAAALASASIVFRNNPTYSKKLVSGAKTVFAFARDGGRRTAYSRGNPYIAPYYNSTNYYDEHMWGAAWLFYATGNSSYFSLATNPGIPRNAKAFYMIPDLSVLSWENKLPAAMLLLTRIRLFLSPGYPYEDMLRSYHNITGLTMCSYLKRYNVFNWTRGGMIELNHGRAQNLQYVANAAFLASVFADYLNASGVPGWYCGGDFLPISILKDFATSQLNYILGANPMKMSYVVGYGDKYPKHVHHRGASIPNDKTRYSCTGGMRWRDAKTANPNNIVGAMVGGPDRFDNFKDVRTNYSYTEPTVAGNAGLVAALVSLTSSGGDGVDKNTMFSAVPPMYPANPPPPPPWKP*

>Peb22677

MVFKSWTSVSIAGNSDWFAKQADLVKKVVVTMPAHLQVNHKETVKDHQSLVICNSEVFYVPRRFVSDFIDLVSLVGDLDIHHKVAVPMFFLAMDMPHNYDSVFDFMIYKQKPQSNSTFYSAEVPAIHPWNVSSEQDFIKLIKIMAAGDPLLMELF*

>Peb22686

MLVQDRDAPKPLNHRSRNKFRLFPPKNLDFSTWVSENIFKLVTILFVITTVSAVFYLRNYYTAGGDAAALLCLQSTQSRSIRPKFPQINWNSIPRIVDNVTPFSAFRSEKWIVVSVSGYPSESLKKMAKIKGWQVLAIGNSKTPEDWKLKGVIYLSLEMQAQLGFRVVDYLPFDSYVRKTVGYLFAIQHGAQKIFDVDDRGDVIDNDIGKHFDVELVGEGSRQEIILQYSHHNPNRTVVNPYIHFGQRSVWPRGLPLENVGEIEHETFYTEVFGGKQFIQQGISNGLPDVDSVFYFTRKSALEGFDIRFDEHASKVALPQGTMVPVNSFNTIFHSSAFWGLMLPVSVSTMASDVLRGYWAQRLLWEVGGYVVVYPPTVHRYDKIEAYPFSEEKDLHVNVGRLIKFLVAWRSSTHRLFEKILELSYVMAEEGFWTEKDLKFTAAWLQDLLSVGYQQPRLMSLELDRPRANIGDGDRKEFVPQKLPSVHLGVEETGVVNYEIGNLITWRKSFGNVVLIMFCYGPVERTALEWRLLYGRIFKTVIILSVEKNVDLAVEQGDADHVYNKDYDLPADQSKARKVAKILQRISPKPIEEFKISPVQSLSERGIQVCSNSDDKDTEEVLESIYIGDSKNTEVLDSEQPINSGEFHQNDVDDFPLEDSDEDFLDDGFDSTDADSHISSHTETLLNNEAIEVDLQALFKENEVIVTPKKSYYPRKY*

>Peb23063

MYGRDPWGGTLEIAADSATDDDRSRNLHEYDRAALSRPLDETQQSWLLGPGEQKKKKYVDLGCVIVSRKIFLWTVGTIVAAGLLAGFIALIVKTVPRHHHRPPPPDNYTLALNKALMFFNAQRSGKLPKHNNVSWRGNSCVNDGESDSTTLFKDLAGGYYDAGDAIKFNFPQSFAMTMLSWSVIEYSAKYEAAGELSHVKDIIKWGTDYFLKTFNNTADTIDRIAMQVGEGDTSGGSTKPNDHYCWTRPEDIDYDRHVLECHSCSDLAAEMAAALASASIVFKDNKAYSQKLVHGARTLFKFARDQRGRYSAGTEASTFYNSTGYWDEFVWGASWLYYATGNSSYLQLATAPGLAKHAGAFWGGPFYGVLSWDNKLAGAQVLLSRLRLFLSPGYPYEEILGTFHNQTSIFMCSFLPDFSTFNRTKGGMIQLNHGAPQPLQYVVNAAFLATLFSDYMKAADTPGWYCGPHFYSTDALRKFAQTQIDYILGKNPRQMSYVVGFGNHYPKHVHHRGASIPKNQVKYSCTGGWKWRDSTKPNPNTIVGAMVAGPDRQDGFHDVRTNYNYTEPTLAGNAGLVAALVALSGDSTMEIDKNTIFSAVPPMFPTPPPPPAPWKP*

>Peb25320

MLVQDRVFSSSDGPKSLNHQSRNKFRLFPPKKLDFSTWLSENIFRIGMIILVITTVAAVFCLRNYYTTGGDAAALLCLQSTQSHSIHPKFPQINWNSIPRVVDKVTPFSSFRSEKWIVVSVSEYPSESLKKMAKIKGWQVLAIGNSRTPEDWKLKGVIYLSLDMQAQLGFRVVDYLPFDSYVRKTVGYLFAIQHGAQKIYDIDDRGDVIDNDIGKHFDVELVGEGSRQEVILQYSHDNPNRTVVNPYIHFGQRSVWPRGLPLENVGDIEHEPFYTEVFGGKQFIQQGISNGLPDVDSVFYFTRKSTLEGFDIRFDQHASKVALPQGTMVPVNSFNTIFHSSAFWGLMLPVSVSSMASDVLRGYWAQRLLWEVGGYVVVYPPTVHRYDKIEAYPFSEEKDLHVNVGRLIKFLVAWRSSNHRLFEKILELSYVMAEEGFWTEKDLKFTAAWLQDLLSVGYQQPRLMSLELDRPRANIGDGDRKEFVPQNLPSVHLGVEETGMVNYEIGNLITWRKNFGNVVLIIFCYGPVERTALEWRLLYGRIFKTVIILSVEKNVDLAVEQGDADHVYKNLPKLFDRYSSADGFLFLQDNTILNYWNLLQADKTKLWITNKVSKSWTSVPIAGNSDWFAKQADLVRKVVATMPAHLQVSHKETVKDHQSLVICNSEVFYVPRRFVSDFIDLVSLVGNLDIHHKVAVPMFFLAMDSPQNYDSVFDSMRYKQKPQSNSTFYSPEAPAVHPWNVSSEQDFIKLIRLMAAGDPLLMELF*

>Peb27085

MGVAMHTKSDSEVTSIEASTPPRSPRRPLYYVQSPSHSQHDLEKMSYGSSPFGSPAHHFQYHCSPIHHSRESSTSRYSASLKNPRNIGGGWKRMQRKYDELGDEDGEDVEGDEGDMKDGGQVRFYAVCFLFSFVVLFSTFSLILWGASLAYKPRIIVKNILFENFNVQAGMDSTGVPTDMLSLNSTVRIFYRNPSTFFGVHVSATPLELHYFDLKVASGQVRNFYESRKSQRTVMPVVEGHQVPLYGGIPLLSGTKGHLESTPVPLNLTFVMRSRAYILGRLVKTKFYRSVLCQVIVRGTHLGKPLNLTKSQSCVYRS*

>Peb27521

MLHTKSESDITSLAPSSPSRSPKRAYYVQSPSRDSQDGDKSSSMHATPNFRSPMESPSHPSVGRHSRNSSSSRFSGIFRSSSGRKSGRKRMNDKGWPECNVILEEGNYDELNDKAHMRCCQALMAFLGFIVLFSVFCLIIWGAGRSYKAEVAVRSLSVNNLYIGSGADFTGVPTNMLNLNGSLRISIYNPATFYGIHVSATPVNLIYTDVVVATGQLKKYFQPRKSHRTMLVHIKGTKVPLYGAGSTLVISNNAFKVPLTLEFEIRSRGDVGGETGEDQTS*

>Peb27718

MMEAGALEILSDKFARHANRDQAVFEDTEGIWISALLAAILFQDTDAVSSSTAMSFIQSLSVLFNSAEMIDRFFAAQAYASLVGHENKEINLAIANSGTVSGLMSLIGHIESDMPNVMALSEEFSLVKNPDQIVLESLFQIDEIRVGPVSQNTIPLLVDLLRPIPNRPGAPSFSVRLLTKIAHGNDTNKLFIAEAGALDALTKYLSLSPQDLTEATVSELLRIMFSNPDLVQYESALSCMNQLVAVLHLGSKSARLSAARAINELFDSKEIRDSEASIQAIQPLADMLDTPFEYEQQTALSALIKLTSDNYAKAAMLAEVEGNLLVCLHKIISSVANLKLKSDAAKLCCILFGNSRIRESPITSECIGPLILLMQSDEETAVESSVRALGRLLEDENQVDITSSHDLVGKLVYLVSGTNHLLIEASISTLIKLGKDRTPRKLEMVNLGIIDNCLKLLPTAPNSLCTTIAELFRILTNSSAISKSLAAARSVEPFFMLLFKTDFDLQGQHSALQVLVNILEKPQSLSRCKLTPNQVIEPLISVLESPSQAIQQLGAELLSHFLDQEHFPQDMLTKNAILPLVHLVGIGILNLQQTAIKALENISLSWPKEVSDAGGIFELSKVIVQEDPLPSDDLLESAALVLSNLLRFDADYYFRVPVGVLVKMLYSGLESTVVVAINSLIVQEKADSSSAELMAEAGAIDALLDLVRSHHCEEVSGTLLEELFNNTRVREMKACKYAIAPLAQYLLDPQTRSENGRLLAALALGNLSQHEGLARASDSASACRALVFLLEDQPTKDMQIVTVCALQNFVMHSRTNRRTVAEAGGVLRIQELLLSSDSEVVDQTALLVRYLFSNHTLQEYASNELIKSLTAVLEKEESSRSSVKEEEILKTIHVILSNFHKLHVSEATTLCIPHLLTALKSGTEAAQDSALTTLCLLKHTWSTIPIDMSKSQAMVAAEAIPFLQIHMKTCPPNFLDRIESLLHSLPGCLTVAIKRADNLKQVTGGTNAFCQLSIGHCPARYTKVVSHNTSPEWEEKFTWAFDVPPKGQKLNIVCRSRNTFGKTTLGRVTIPIDKVVNEGSYSDLLNLGQDTNKDSSSRTLELEMAWSNSTLNENV*

>Peb27892

MIWAWIDIPFINTILIIMQVDLSTIVVYEIAWVPNGTTEADDPDISRVAQLVDQLHANVTSAHEKELTTARLLGIAKTRKEARAFIISHDQAMPLFVSILRNGTLPAKINVAATLSILCRTKDFRVKVLLGGCIPPLLSLLKSNATEARKAAAEAIYAVSSSGISDDQVGMKIFVTEGVVQTLWDQLNAKNNDKTVEGFVTGALRNLCGDKEGYWQAILDVGGVDIIVGLLSSENSTSQSNAASLLAHMILDFPDSVMKIVDSGAVKSLLRLLSDKKDVTVRASAAEALEALSIKSKKGKESIVDAQGMPLLIGVVVAPSKEGIQGEVAEALQRHSMQTLANICGGMFKLIIHLGELSQSSRLAAPVADIIGALAHCLMVFKQNGDVEPFESTKIESILVMLLKPRDNKQVQERLLEAMAGLYGNAYLSVSISQSEAKKVLVGLITMVSGDAQECFIFSLIRLCTEGVSVWEALGKREGIQLLISSFGLSNEKHQEYAVKMLSILTKQVDDSKWALTSAGGVPPLVLLLEVGSEKARNDAAHILWNLCHYSEEIRVILESAGAVPAFIWLLKNGGPEEQEVSANTLIKLIKNGNPSSINQLLALLLGDSPSAKTHVIKVLGHVLSMASLSDLVNPGATAYKGLKALVQLFNSSNERTREHAALVLADLFINRQDLCDGLKTDEAVNPCMKILTSNTQGIATKFPQALNSVSHPIKFKKMSKTSYISEGDIKPLIRKAKTLQIDSAERAMAALSYELSDPQVAAEALAEDIVSAITRVLGEGSLEGKQSASRALHQLLKHFPVGDVLTGSSQCRFIVLEVIDSLNATDVDSTEFLDTLEVVSLLSRTKLGHSSYSPWSGLAEVSSSLDPLLHCLCEGPTSVQDKAIEILSRLSTDQPAALGDVLVSNSKSIGNLAIRIMNSRSQEVRVGGTTLLICAAKEHKIQSIHALEASGYLKPLIYTLVDMMKQNSSCSSVEIEVRTPRAYRDRSDFQG*

>Peb27916

MDTKGRLVAGSHNRNEFVLINADEIGRVTSVKELTGQICQICGDEIEFGVDGEPFVACNECAFPVCRPCYEYERREGNQACPQCKTRYKRIKGSPRVDGDDEEEEFDDLENEFDYNSNERRDLHQIAEAALAARLNVSRGSSGICTSSSELDPSAVNSDIPLLTYGQEDDTISADKHALIIPPFMGRGKRVHPMAFSDSSMTVPPRPMDPKKDLAVYGYGTVAWKERMDEWKKKQNDRLQVVKHQGDKGGDELDDPDLPKMDEGRQPLSRKLPIPSSKINPYRMIILLRMAILGLFFHYRVLHPVNDAYGLWLTSIICEIWFAVSWLFDQFPKWSPILRETYLDRLSLRYEKEGKPSELMPVDIFVSTVDPMKEPPLITANTVLSILAVDYPVDKVACYVSDDGAAMLTFEALSETCEFARKWVPFCKKFSIEPRAPEWYFAQKVDYLKDKVEPTFVRERRAMKREYEEFKIRINGLVATAQKVPEDGWTMQDGTPWPGNNVRDHPGMIQVFLGQNGVQDIEGNELPCLIYVSREKRPGFEHHKKAGAMNALIRVSAVISNAPYLLNVDCDHYINNSKALREAMCFMMDPQAGKKICYVQFPQRFDGIDRHDRYSNRNVVFFDINMKGLDGIQGPIYVGTGCVFRRQALYGYDAPKKAKPPGKTCNCWPKWCCFCCGSRKKSKKAKSKNNKKKTKSREASTQVHALENIEQGIEGIESEKSTLMPQIKFEKKFGQSPVFIASTLLEEGGVPPGASSASLLKEAIHVISCGYEDKTDWGKEVGWIYGSVTEDILTGFKMHCHGWRSVYCIPHRPAFKGSAPINLSDRLHQVLRWALGSVEIFFSRHCPLWYGYGCGLKPLERFSYINSVVYPLTSLPLIVYCTLPAVCLLTGKFIVPEISNYASIIFMGLFISIAATSILEMQWGGVGIDDWWRNEQFWVIGGVSSHFFALIQGLLKVLAGVSTNFTVTSKAADDGEYSELYLFKWTSLLIPPMTLMIINIIGVIVGISDAINNGYETWGPLFGRLFFALWVIVHLYPFLKGFMGKQDRVPTIIVVWSILLASILTLLWVRINPFLSKDGIILEVCGLNCD*

>Peb29348

MGKRGFAAILVALMVVHCACMVSAEDEIIVKKIGGKRVCRQGWECEGFSEHCCNETISKFFQTYQFENLFSKRNSPVAHAVGFWDYKSFILASSVYQPLGFGTTGGKLMQMKEVAAFLGHVGAKTTCGYGVATGGPLAWGLCYNKEMSPSQDYCDEFFKYEYPCAPGAQYHGRGAIPVYWNYNYGKLGKDIKVDLLNHPEYLEQNATTAFMAAMSMWMTSKKKGQPSAHDVFVGNWKPTKNDTMEKRVPGFGATMNILYGDLVCGQGEIDPMNVCISHYQYYLDLMGVGREEAGPHEVLSCGEQTAFNPTYKSSS*

>Peb29722

MMMGNRRIMVALLIPAMVVYCACMVSGDDEIKEKKVGGKRMCDQGWECKGFSAYCCNQTISKFFQTYQFENIFSKRNSPVAHAVGFWDYRSFILSSAVYQPLGFGTTGGKLMQMKELAAFLGHVGAKTSCGYGVATGGPLSWGLCYNKEMSPSQDYCDDFFKYEYPCSPGAQYYGRGAIPVYWNHNYGKIGNDIKVDLLNHPEYLEQNATIAFMSAMSMWMTPKKKGQPSAHDAFVGNWKPTKNDTMEKRVPGFGTTMNILYGDLICGQGEIDPMNVLISHYLYYLDLMGVGREEAGPHEVLSCGEQIAFNPSYKSSS*

>Peb31490

MMEPGVLSCSTCGEKLGLSSNGRLFVACHECNDPICRYCVDYEIKQGRNTCMRCGTPYNNDGKEETDSGEKESGNHMKMASRLDTVEDTGIHARNISTVSTVDSEYIDDSGNPIWKNRVESWKDKKNKKKHATKEKTEAQIPSEQQMEEKPQSIDASQPLSRVVPLPKSQLTPYRIVIIMRLIILAFFFNYRVTNPVDSAFGLWLTSVVCEIWFAFSWVLDQFPKWSPVNRDTYIDRLSARYEREGEPSELAAVDFFVSTVDPLKEPPLITSNTVLSILAIDYPVDKVSCYVSDDGAAMLTFESLAETADFARKWVPFCKKYSIEPRAPEFYFSQRIDYLKDKIQPSFVKERRAMKRDYEEYKVRVNALVAKAQKTPDDGWTMADGTSWPGNNTRDHPGMIQVFLGNTGAHDIEGNELPRLVYVSREKRPGYQHHKKAGAENALVRVSAVLTNAPYILNLDCDHYVNNSKAIREAMCFLMDPQVGPDVCYVQFPQRFDGIDKSDRYANRNTVFFDVNMKGLDGIQGPVYVGTGCVFNRQALYGYGPLSLPTIPKASSSSCSWCGSCCCCCRGKKSVKEKNLSEVHRDERREDLNAAIFNLREIESYDEHERSLLISQMSFEKTFGLSPVFIESTLMENGGVAESANPSTLIKEAIHVISCGYEEKTAWGKEIGWIYGSVTEDILTGFKMHCRGWRSIYCMPLRPAFKGSAPINLSDRLHQVLRWALGSVEIFLSRHCPLWYGFGGGRLKWLQRLAYINTIVYPFTSLPLVAYCSLPAICLLTGKFIIPTLSNLASILFLGLFLSIIVTSVLELRWSGVSIEALWRNEQFWVIGGVSAHLFAVFQGFLKMLAGIDTNFTVTAKAADDADFGELYVFKWTTVLIPPTTILIVNLVGVVAGFSDALNSGYESWGPLFGKVFFAFWVILHLYPFLKGLMGRQNRTPTIVVLWSVLLASVFSLVWVKINPFVSTNDASTVAQNCIDIDC*
