# Supplementary material for: Genome-wide analysis of the cellulose toolbox of Primulina eburnea, a calcium-rich vegetable
Source: BMC Plant Biol. 2023 May 16;23:259. doi: 10.1186/s12870-023-04266-z (PMC10186795; doi:10.1186/s12870-023-04266-z)
Supplement: Supplementary file 2 — Supplementary Material 2 [file 12870_2023_4266_MOESM2_ESM.docx]

**Supplementary Figures**

**Fig. S1. Representative seedling and leaves.** (a) shows the representative seedling for sample collection. (b) shows the leaves for cellulose analysis.

**
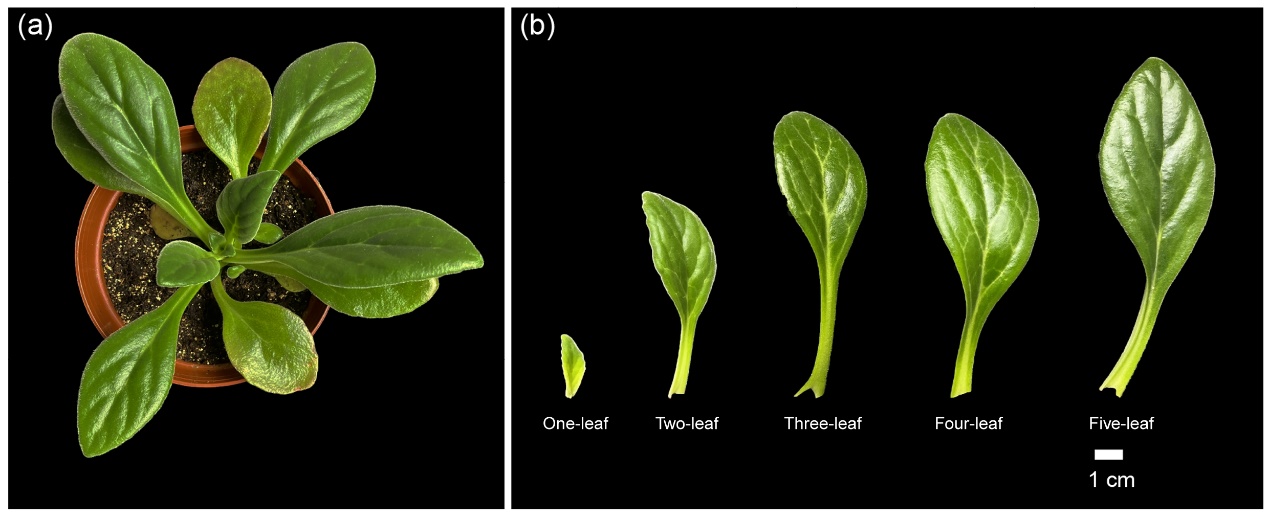
**

**Fig. S2. Water contents during the development of *Primulina eburnea* leaves.** Mean values and standard deviations (SDs) were obtained from eight biological replicates. The error bars indicate standard deviation. Statistical significance was determined by ANOVA.

**
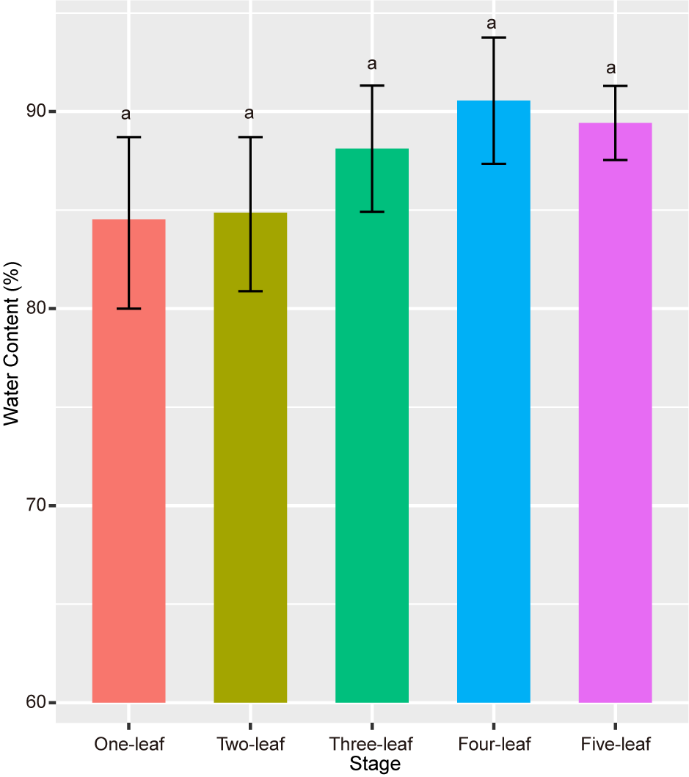
**

**Fig. S3. Synteny relationship of cellulose biosynthesis-involved genes.** Different gene families are shown in different colors. Black dots indicate tandem duplication genes.


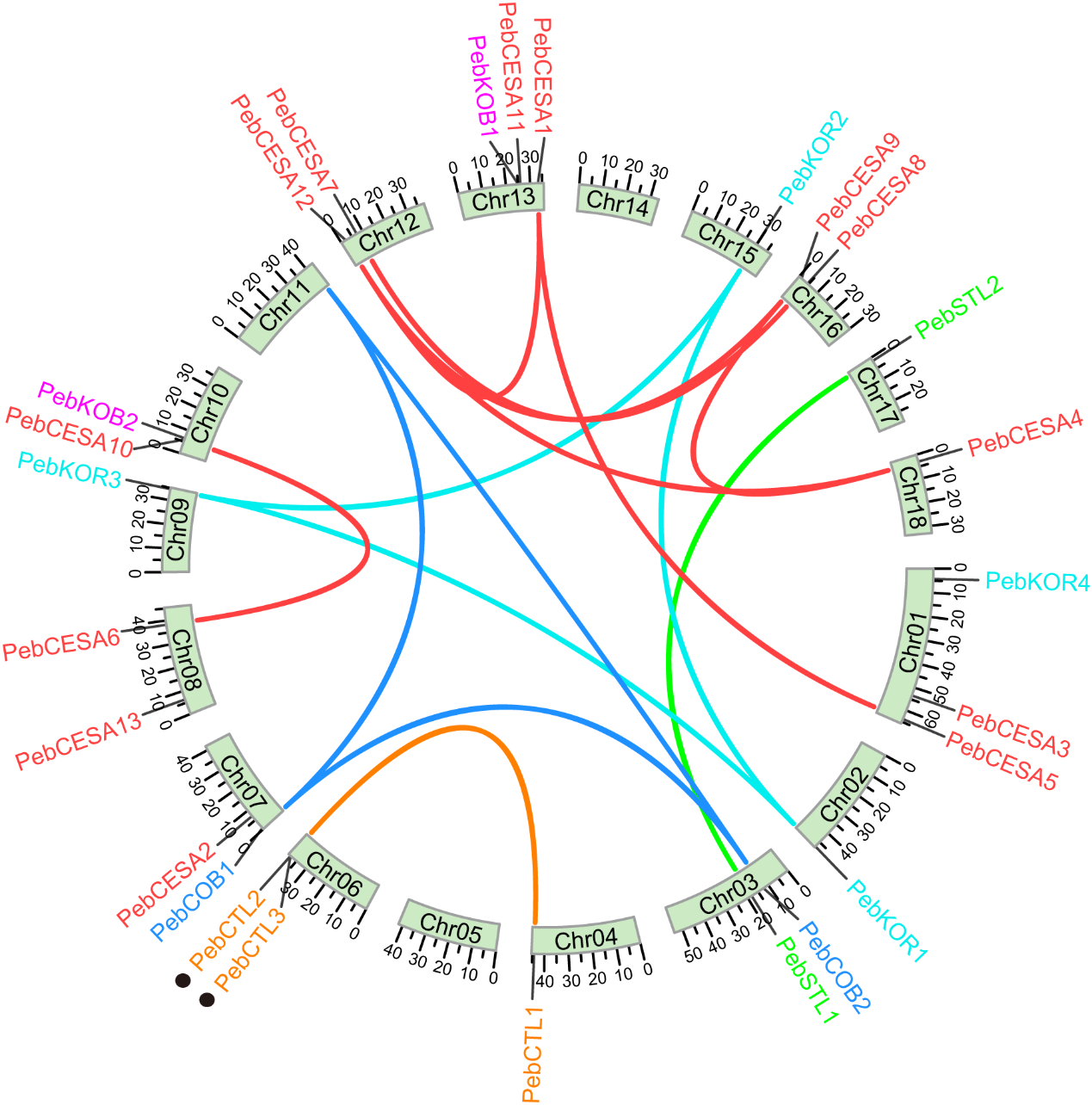


**Fig. S4. Chromosome distribution of cellulose biosynthesis-involved genes.** Different gene families are shown in different colors.


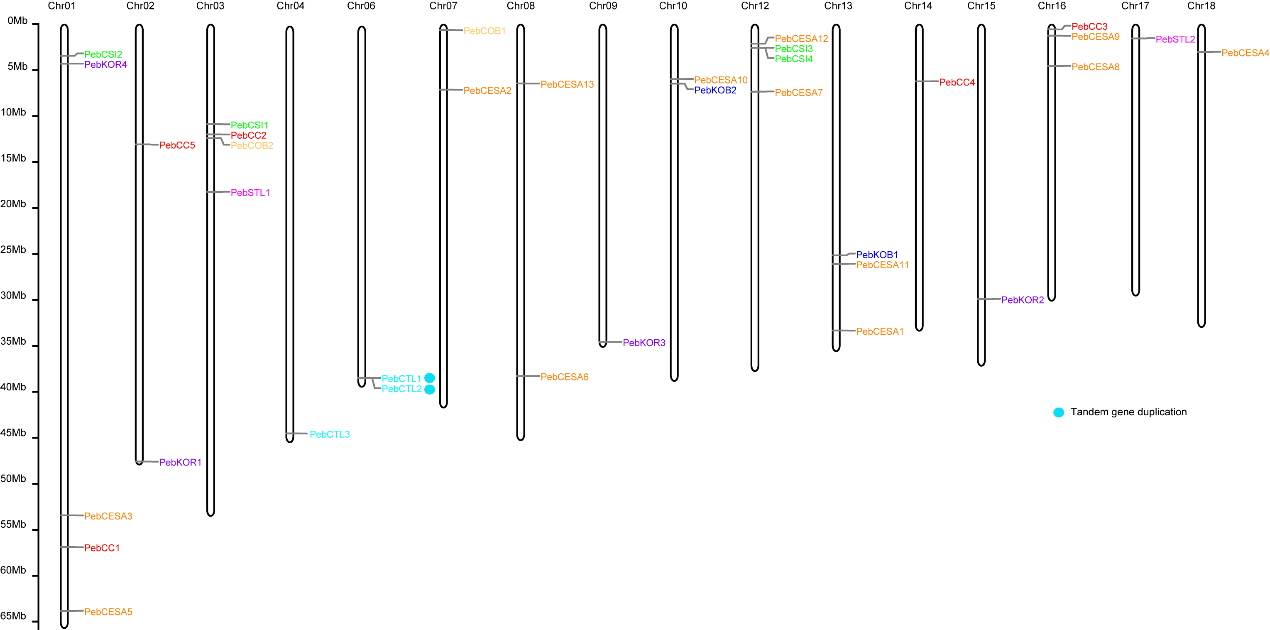


**Fig. S5. Interaction between cellulose biosynthesis-involved genes and transcription factors (TFs).** (a) The top 15 TF families with the highest number of members co-expressed with cellulose biosynthesis involved genes in *P. eburnea*. (b) Potential interacting networks between the cellulose biosynthesis-involved genes and transcription factors. Pearson correlation coefficient (PCC) values were calculated, and a PCC value over 0.9 was used to determine potential interactions.


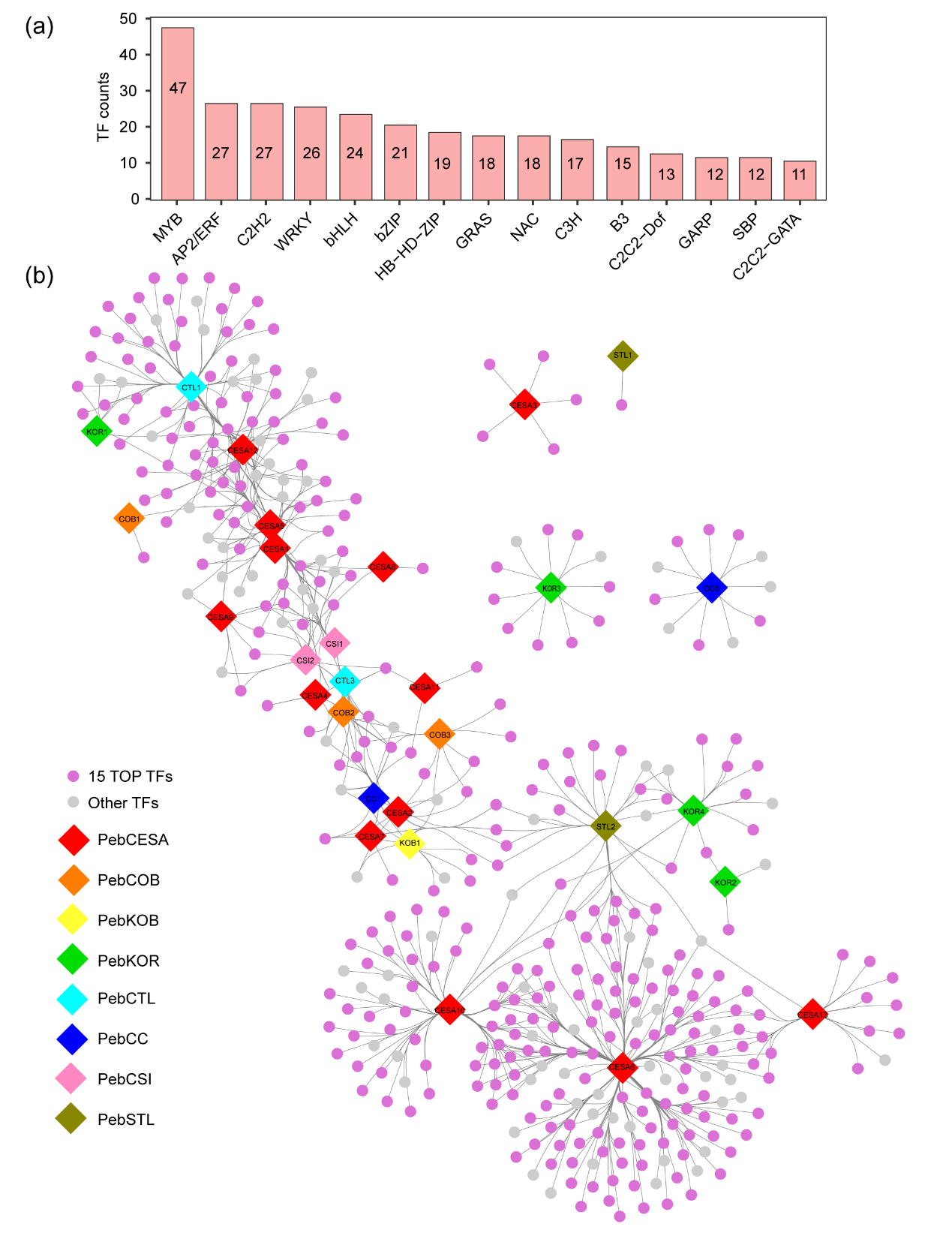


**Fig. S6. Interaction among the cellulose biosynthesis-involved genes in *Primulina eburnea*.** A PCC value of over 0.9 was used to determine potential interactions.


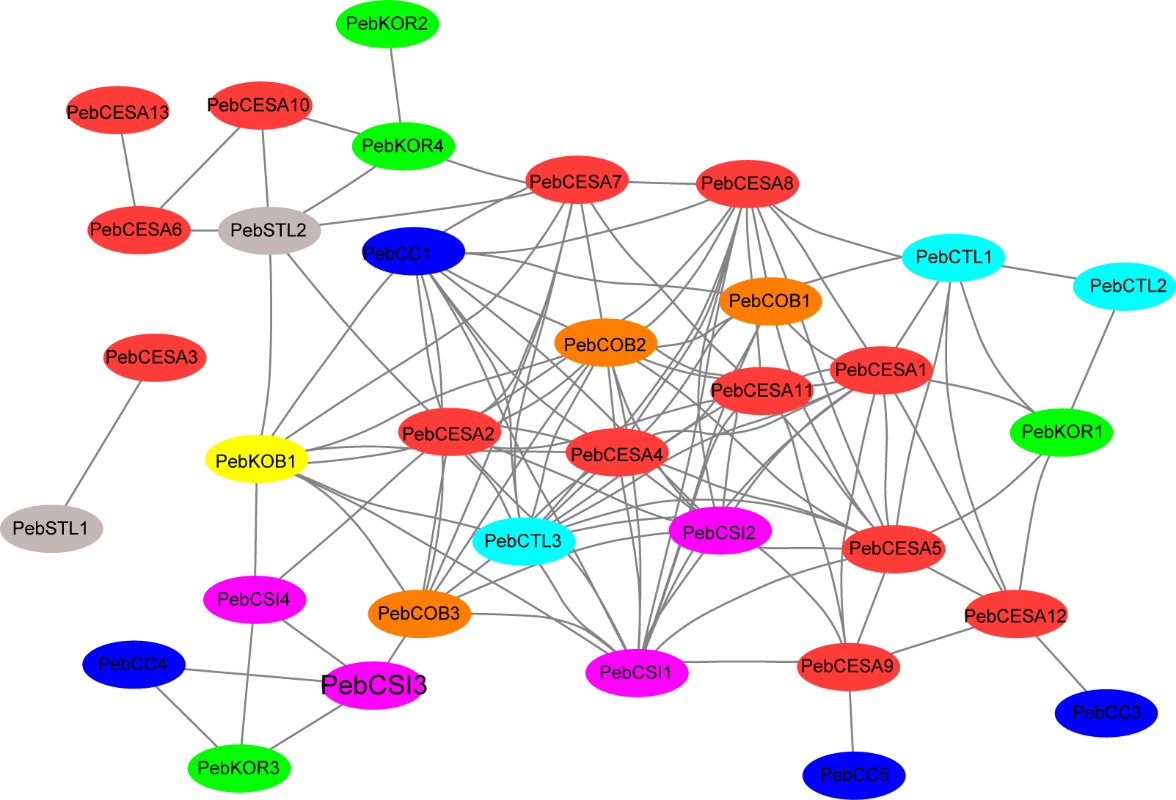


**Fig. S7.** Relative expression of eight cellulose biosynthesis involved genes in different tissues via qRT-PCR and RNA-seq.


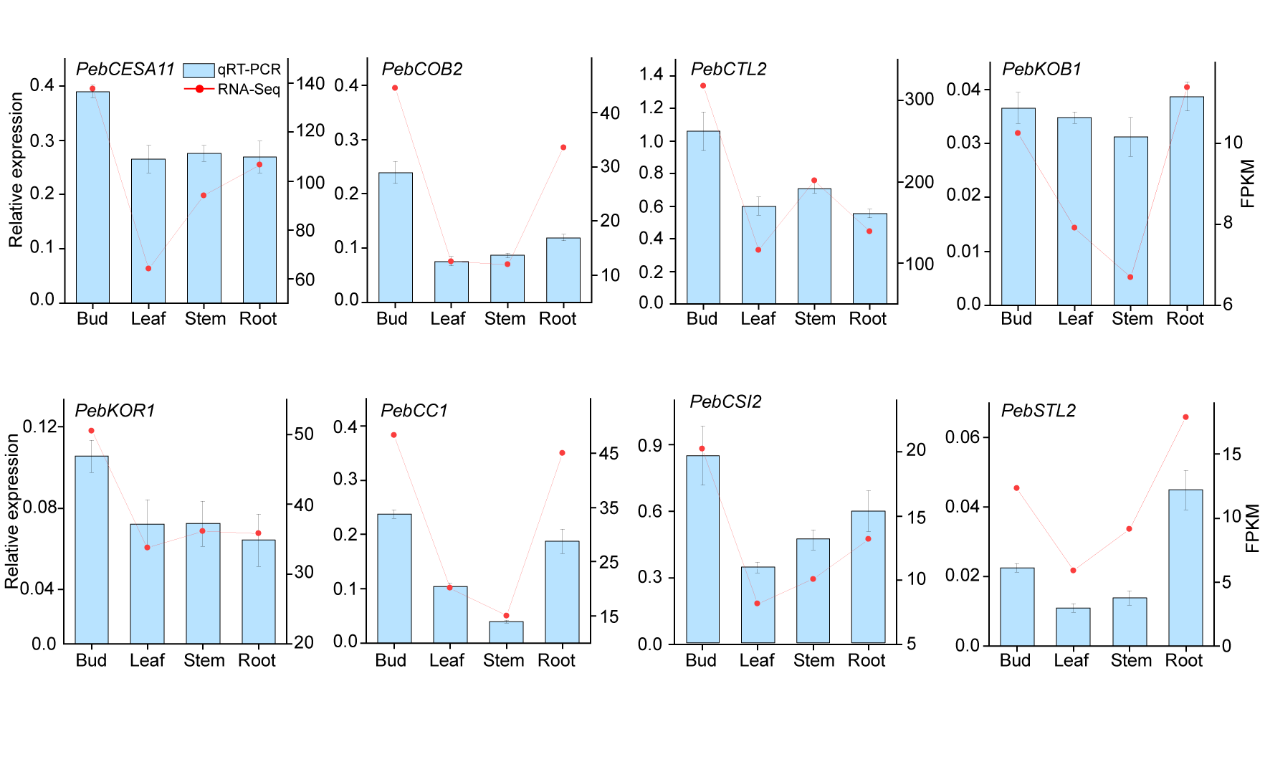


**Supplementary Tables**

**Table S1.** **Features of cellulose biosynthesis involved genes in *Primulina eburnea*.**

| Gene name | Gene ID | Strand | CDS(bp) | Exon | Protein length (aa) | pI | MW (Da) | Predicted location |
| --- | --- | --- | --- | --- | --- | --- | --- | --- |
| PebCESA1 | Peb01198 | - | 3276 | 14 | 1091 | 7.03 | 123464.12 | plas |
| PebCESA2 | Peb07888 | - | 3243 | 14 | 1080 | 5.95 | 122436.57 | plas |
| PebCESA3 | Peb09927 | + | 2541 | 9 | 846 | 8.76 | 96153.85 | plas |
| PebCESA4 | Peb12177 | - | 3279 | 14 | 1092 | 6.48 | 123701.33 | plas |
| PebCESA5 | Peb14384 | - | 3030 | 13 | 1009 | 6.4 | 114215.36 | plas |
| PebCESA6 | Peb14384 | - | 3030 | 13 | 1009 | 6.4 | 114215.36 | plas |
| PebCESA7 | Peb14643 | + | 3327 | 15 | 1108 | 6.74 | 125061.33 | plas |
| PebCESA8 | Peb15047 | + | 3258 | 14 | 1085 | 6.37 | 122331.16 | plas |
| PebCESA9 | Peb15092 | - | 3273 | 14 | 1090 | 6.63 | 123442.4 | plas |
| PebCESA10 | Peb17144 | + | 3132 | 13 | 1043 | 6.31 | 118204 | plas |
| PebCESA11 | Peb17602 | - | 3198 | 15 | 1065 | 7.21 | 119409.96 | plas |
| PebCESA12 | Peb27916 | - | 3273 | 14 | 1090 | 6.63 | 123163.02 | plas |
| PebCESA13 | Peb31490 | + | 2955 | 13 | 984 | 6.42 | 110706.04 | plas |
| PebCSI1 | Peb12918 | + | 4500 | 5 | 1499 | 5.33 | 161146.30 | nucl |
| PebCSI2 | Peb20914 | + | 6294 | 5 | 2097 | 5.15 | 224369.25 | chlo |
| PebCSI3 | Peb27718 | + | 3348 | 5 | 1115 | 5.14 | 122013.24 | cyto |
| PebCSI4 | Peb27892 | + | 2979 | 2 | 992 | 5.93 | 107074.66 | plas |
| PebCC1 | Peb08779 | + | 909 | 3 | 302 | 9.74 | 33383.92 | chlo |
| PebCC2 | Peb13255 | - | 924 | 3 | 307 | 9.70 | 33638.32 | chlo |
| PebCC3 | Peb15480 | + | 1095 | 3 | 364 | 9.86 | 40100.92 | vacu |
| PebCC4 | Peb27085 | + | 957 | 3 | 318 | 9.41 | 35715.69 | chlo |
| PebCC5 | Peb27521 | - | 846 | 3 | 281 | 9.59 | 30841.90 | chlo |
| PebSTL1 | Peb22686 | + | 2154 | 3 | 717 | 5.38 | 81943.90 | cyto |
| PebSTL2 | Peb25320 | + | 2298 | 4 | 765 | 6.61 | 87588.30 | chlo |
| PebCOB1 | Peb07246 | + | 1371 | 6 | 456 | 9.07 | 51368.35 | plas |
| PebCOB2 | Peb13264 | - | 1353 | 6 | 450 | 8.86 | 50857.43 | plas |
| PebCOB3 | Peb17720 | - | 1347 | 6 | 448 | 8.75 | 50570.17 | vacu |
| PebKOR1 | Peb04157 | - | 1851 | 6 | 616 | 8.39 | 68402.17 | chlo |
| PebKOR2 | Peb14536 | - | 1656 | 6 | 551 | 8.96 | 61760.95 | E.R. |
| PebKOR3 | Peb23063 | - | 1851 | 6 | 616 | 7.98 | 68320.02 | E.R. |
| PebKOR4 | Peb20975 | - | 1974 | 6 | 657 | 8.91 | 72867.94 | chlo |
| PebKOB1 | Peb09059 | - | 1260 | 9 | 419 | 6.83 | 48230.95 | mito |
| PebKOB2 | Peb17365 | - | 1476 | 11 | 491 | 6.44 | 55653.4 | mito |
| PebCTL1 | Peb18699 | - | 951 | 3 | 316 | 7.46 | 35148.39 | chlo |
| PebCTL2 | Peb29348 | - | 948 | 3 | 315 | 6.74 | 34944.97 | extr |
| PebCTL3 | Peb29722 | - | 957 | 3 | 318 | 7 | 35480.7 | vacu |

**Table S2. Expression level (FPKM) of cellulose biosynthesis-involved genes.** Expression of cellulose biosynthesis-involved genes in various tissues of *Primulina eburnea* via RNA-seq.

| Gene | Bud | Leaf | Stem | Root |
| --- | --- | --- | --- | --- |
| PebCESA1 | 70.7085 | 14.0096 | 9.7761 | 19.5209 |
| PebCESA2 | 1.1325 | 0.1499 | 0.4259 | 0.1825 |
| PebCESA3 | 9.9464 | 9.8556 | 1.7676 | 14.1190 |
| PebCESA4 | 6.3256 | 3.1111 | 1.5058 | 2.0766 |
| PebCESA5 | 46.3068 | 8.7579 | 4.3398 | 11.0808 |
| PebCESA6 | 1.0410 | 0.3128 | 1.1222 | 1.8636 |
| PebCESA7 | 37.4890 | 13.6098 | 3.9028 | 18.2470 |
| PebCESA8 | 98.1703 | 35.5672 | 8.2911 | 35.2555 |
| PebCESA9 | 14.1356 | 4.6295 | 1.5892 | 8.1268 |
| PebCESA10 | 7.2239 | 4.5693 | 1.1712 | 8.2507 |
| PebCESA11 | 137.5751 | 64.2150 | 10.2534 | 94.0556 |
| PebCESA12 | 15.0519 | 5.3873 | 0.5357 | 5.9174 |
| PebCESA13 | 1.7409 | 0.9769 | 1.0138 | 3.7820 |
| PebCSI1 | 15.2773 | 4.5798 | 3.4328 | 9.5381 |
| PebCSI2 | 20.2295 | 8.1502 | 10.0452 | 13.1756 |
| PebCSI3 | 0.1039 | 0.0342 | 0.0816 | 0.1937 |
| PebCSI4 | 0.0209 | 0.0107 | 0.0000 | 0.0482 |
| PebCC1 | 43.3152 | 15.1253 | 9.9951 | 39.9814 |
| PebCC2 | 0.1113 | 0.0571 | 0.0427 | 0.0212 |
| PebCC3 | 39.0782 | 26.7332 | 32.1196 | 22.8378 |
| PebCC4 | 0.4142 | 0.4483 | 0.3182 | 1.1479 |
| PebCC5 | 32.2481 | 13.1770 | 29.7170 | 27.9381 |
| PebSTL1 | 9.4684 | 6.2405 | 11.7300 | 18.1139 |
| PebSTL2 | 12.3410 | 5.8631 | 9.1234 | 17.8985 |
| PebCOB1 | 6.3765 | 2.6068 | 0.5880 | 0.7319 |
| PebCOB2 | 44.4460 | 12.5720 | 7.0864 | 12.0104 |
| PebCOB3 | 27.5135 | 17.1941 | 6.5500 | 17.9343 |
| PebKOR1 | 50.4624 | 33.7551 | 2.0577 | 36.1478 |
| PebKOR2 | 17.7511 | 8.2051 | 1.1813 | 17.7113 |
| PebKOR3 | 45.0151 | 23.1598 | 9.3903 | 48.0521 |
| PebKOR4 | 2.1099 | 3.2806 | 5.9116 | 0.6466 |
| PebKOB1 | 10.2473 | 7.9018 | 1.1650 | 6.6827 |
| PebKOB2 | 0.0073 | 0.0000 | 0.0000 | 0.0000 |
| PebCTL1 | 69.8425 | 25.0300 | 15.4325 | 16.7540 |
| PebCTL2 | 317.3169 | 115.6323 | 138.5957 | 201.4707 |
| PebCTL3 | 138.3373 | 12.1201 | 84.6723 | 18.1599 |

**Table S3.** **Gene Similarity.** Similarity between gene pairs of each family.

| Gene1 | Gene2 | Similarity | Gene1 | Gene2 | Similarity |
| --- | --- | --- | --- | --- | --- |
| PebCESA1 | PebCESA5 | 96.615 | PebCESA9 | PebCESA8 | 63.799 |
| PebCESA1 | PebCESA12 | 89.122 | PebCESA9 | PebCESA6 | 64.033 |
| PebCESA1 | PebCESA9 | 88.848 | PebCESA9 | PebCESA3 | 71.868 |
| PebCESA1 | PebCESA4 | 88.498 | PebCESA9 | PebCESA13 | 67.171 |
| PebCESA1 | PebCESA2 | 76.325 | PebCESA10 | PebCESA6 | 93.774 |
| PebCESA1 | PebCESA10 | 64.936 | PebCESA10 | PebCESA11 | 68.866 |
| PebCESA1 | PebCESA7 | 64.17 | PebCESA10 | PebCESA7 | 66.851 |
| PebCESA1 | PebCESA11 | 65.985 | PebCESA10 | PebCESA8 | 66.575 |
| PebCESA1 | PebCESA8 | 63.375 | PebCESA10 | PebCESA12 | 65.091 |
| PebCESA1 | PebCESA6 | 63.297 | PebCESA10 | PebCESA5 | 65.273 |
| PebCESA1 | PebCESA3 | 72.695 | PebCESA10 | PebCESA1 | 64.572 |
| PebCESA1 | PebCESA13 | 66.474 | PebCESA10 | PebCESA9 | 65.123 |
| PebCESA2 | PebCESA12 | 76.143 | PebCESA10 | PebCESA4 | 64.633 |
| PebCESA2 | PebCESA1 | 76.325 | PebCESA10 | PebCESA2 | 63.795 |
| PebCESA2 | PebCESA5 | 76.164 | PebCESA10 | PebCESA3 | 73.821 |
| PebCESA2 | PebCESA9 | 76.417 | PebCESA10 | PebCESA13 | 62.705 |
| PebCESA2 | PebCESA4 | 75.859 | PebCESA11 | PebCESA7 | 71.575 |
| PebCESA2 | PebCESA7 | 64.292 | PebCESA11 | PebCESA8 | 71.177 |
| PebCESA2 | PebCESA11 | 64.419 | PebCESA11 | PebCESA10 | 68.779 |
| PebCESA2 | PebCESA8 | 63.067 | PebCESA11 | PebCESA6 | 67.073 |
| PebCESA2 | PebCESA10 | 63.703 | PebCESA11 | PebCESA12 | 65.153 |
| PebCESA2 | PebCESA6 | 61.818 | PebCESA11 | PebCESA5 | 65.677 |
| PebCESA2 | PebCESA13 | 57.91 | PebCESA11 | PebCESA9 | 64.75 |
| PebCESA2 | PebCESA3 | 71.158 | PebCESA11 | PebCESA1 | 65.456 |
| PebCESA3 | PebCESA11 | 73.949 | PebCESA11 | PebCESA4 | 64.74 |
| PebCESA3 | PebCESA10 | 74.205 | PebCESA11 | PebCESA2 | 64.393 |
| PebCESA3 | PebCESA13 | 73.223 | PebCESA11 | PebCESA13 | 63.39 |
| PebCESA3 | PebCESA7 | 72.835 | PebCESA11 | PebCESA3 | 73.38 |
| PebCESA3 | PebCESA5 | 72.931 | PebCESA12 | PebCESA9 | 96.609 |
| PebCESA3 | PebCESA8 | 72.588 | PebCESA12 | PebCESA1 | 89.122 |
| PebCESA3 | PebCESA1 | 72.104 | PebCESA12 | PebCESA5 | 88.848 |
| PebCESA3 | PebCESA12 | 72.104 | PebCESA12 | PebCESA4 | 87.524 |
| PebCESA3 | PebCESA9 | 71.868 | PebCESA12 | PebCESA2 | 76.143 |
| PebCESA3 | PebCESA2 | 71.395 | PebCESA12 | PebCESA7 | 64.531 |
| PebCESA3 | PebCESA4 | 73.086 | PebCESA12 | PebCESA11 | 65.584 |
| PebCESA3 | PebCESA6 | 78.436 | PebCESA12 | PebCESA10 | 65.455 |
| PebCESA3 | PebCESA6 | 80.925 | PebCESA12 | PebCESA8 | 63.978 |
| PebCESA4 | PebCESA1 | 88.498 | PebCESA12 | PebCESA6 | 63.967 |
| PebCESA4 | PebCESA5 | 87.928 | PebCESA12 | PebCESA3 | 72.104 |
| PebCESA4 | PebCESA12 | 87.524 | PebCESA12 | PebCESA13 | 66.821 |
| PebCESA4 | PebCESA9 | 87.429 | PebCESA13 | PebCESA11 | 63.91 |
| PebCESA4 | PebCESA2 | 75.859 | PebCESA13 | PebCESA10 | 62.488 |
| PebCESA4 | PebCESA7 | 65.084 | PebCESA13 | PebCESA3 | 72.857 |
| PebCESA4 | PebCESA10 | 65.491 | PebCESA13 | PebCESA7 | 70.548 |
| PebCESA4 | PebCESA8 | 64.44 | PebCESA13 | PebCESA6 | 61.412 |
| PebCESA4 | PebCESA11 | 65.488 | PebCESA13 | PebCESA8 | 70.023 |
| PebCESA4 | PebCESA6 | 63.654 | PebCESA13 | PebCESA5 | 67.206 |
| PebCESA4 | PebCESA3 | 72.84 | PebCESA13 | PebCESA1 | 66.859 |
| PebCESA4 | PebCESA13 | 66.706 | PebCESA13 | PebCESA9 | 67.053 |
| PebCESA5 | PebCESA1 | 96.615 | PebCESA13 | PebCESA12 | 66.667 |
| PebCESA5 | PebCESA12 | 88.848 | PebCESA13 | PebCESA2 | 65.896 |
| PebCESA5 | PebCESA9 | 88.483 | PebCESA13 | PebCESA4 | 66.786 |
| PebCESA5 | PebCESA4 | 87.928 | PebCSI1 | PebCSI2 | 79.441 |
| PebCESA5 | PebCESA2 | 76.164 | PebCSI1 | PebCSI3 | 47.52 |
| PebCESA5 | PebCESA11 | 66.05 | PebCSI1 | PebCSI4 | 26.171 |
| PebCESA5 | PebCESA7 | 64.376 | PebCSI2 | PebCSI1 | 79.441 |
| PebCESA5 | PebCESA10 | 65.273 | PebCSI2 | PebCSI3 | 47.971 |
| PebCESA5 | PebCESA8 | 63.669 | PebCSI2 | PebCSI4 | 40.682 |
| PebCESA5 | PebCESA6 | 63.273 | PebCSI3 | PebCSI2 | 47.971 |
| PebCESA5 | PebCESA3 | 73.404 | PebCSI3 | PebCSI1 | 47.52 |
| PebCESA5 | PebCESA13 | 66.936 | PebCSI4 | PebCSI2 | 40.682 |
| PebCESA6 | PebCESA10 | 93.774 | PebCSI4 | PebCSI1 | 26.171 |
| PebCESA6 | PebCESA11 | 67.291 | PebCC1 | PebCC5 | 73.285 |
| PebCESA6 | PebCESA7 | 64.695 | PebCC1 | PebCC3 | 43.168 |
| PebCESA6 | PebCESA8 | 64.528 | PebCC1 | PebCC2 | 42.765 |
| PebCESA6 | PebCESA1 | 63.115 | PebCC1 | PebCC4 | 40.064 |
| PebCESA6 | PebCESA12 | 63.785 | PebCC2 | PebCC3 | 58.204 |
| PebCESA6 | PebCESA9 | 63.669 | PebCC2 | PebCC4 | 44.72 |
| PebCESA6 | PebCESA5 | 63.273 | PebCC2 | PebCC1 | 44.051 |
| PebCESA6 | PebCESA2 | 61.056 | PebCC2 | PebCC5 | 39.209 |
| PebCESA6 | PebCESA4 | 64.84 | PebCC3 | PebCC2 | 57.585 |
| PebCESA6 | PebCESA4 | 74.757 | PebCC3 | PebCC4 | 47.256 |
| PebCESA6 | PebCESA13 | 58.649 | PebCC3 | PebCC1 | 43.168 |
| PebCESA6 | PebCESA13 | 77.907 | PebCC3 | PebCC5 | 41.115 |
| PebCESA6 | PebCESA3 | 80.397 | PebCC4 | PebCC3 | 46.951 |
| PebCESA6 | PebCESA3 | 80.925 | PebCC4 | PebCC2 | 44.201 |
| PebCESA7 | PebCESA8 | 97.148 | PebCC4 | PebCC1 | 40.064 |
| PebCESA7 | PebCESA11 | 71.668 | PebCC4 | PebCC5 | 39.86 |
| PebCESA7 | PebCESA10 | 66.851 | PebCC5 | PebCC1 | 73.285 |
| PebCESA7 | PebCESA5 | 63.834 | PebCC5 | PebCC3 | 40.07 |
| PebCESA7 | PebCESA12 | 64.44 | PebCC5 | PebCC4 | 40.569 |
| PebCESA7 | PebCESA1 | 63.628 | PebCC5 | PebCC2 | 39.643 |
| PebCESA7 | PebCESA9 | 64.022 | PebSTL1 | PebSTL1 | 100 |
| PebCESA7 | PebCESA6 | 64.695 | PebSTL1 | PebSTL2 | 92.782 |
| PebCESA7 | PebCESA4 | 64.519 | PebSTL2 | PebSTL2 | 100 |
| PebCESA7 | PebCESA2 | 64.11 | PebSTL2 | PebSTL1 | 92.782 |
| PebCESA7 | PebCESA13 | 70.662 | PebCOB1 | PebCOB3 | 84.116 |
| PebCESA7 | PebCESA3 | 72.01 | PebCOB1 | PebCOB2 | 85.151 |
| PebCESA8 | PebCESA7 | 97.148 | PebCOB2 | PebCOB3 | 95.74 |
| PebCESA8 | PebCESA11 | 71.177 | PebCOB2 | PebCOB1 | 85.613 |
| PebCESA8 | PebCESA10 | 65.58 | PebCOB3 | PebCOB2 | 95.74 |
| PebCESA8 | PebCESA5 | 64.029 | PebCOB3 | PebCOB1 | 84.116 |
| PebCESA8 | PebCESA1 | 63.734 | PebKOR1 | PebKOR4 | 94.667 |
| PebCESA8 | PebCESA12 | 63.262 | PebKOR1 | PebKOR2 | 87.818 |
| PebCESA8 | PebCESA9 | 62.814 | PebKOR1 | PebKOR3 | 60 |
| PebCESA8 | PebCESA6 | 63.645 | PebKOR2 | PebKOR4 | 89.091 |
| PebCESA8 | PebCESA4 | 64.245 | PebKOR2 | PebKOR1 | 87.818 |
| PebCESA8 | PebCESA2 | 62.795 | PebKOR2 | PebKOR3 | 58.929 |
| PebCESA8 | PebCESA13 | 70.136 | PebKOR3 | PebKOR4 | 60 |
| PebCESA8 | PebCESA3 | 71.614 | PebKOR3 | PebKOR1 | 60 |
| PebCESA9 | PebCESA12 | 96.609 | PebKOR3 | PebKOR2 | 58.929 |
| PebCESA9 | PebCESA1 | 88.848 | PebKOR4 | PebKOR1 | 94.667 |
| PebCESA9 | PebCESA5 | 88.483 | PebKOR4 | PebKOR2 | 89.091 |
| PebCESA9 | PebCESA4 | 87.429 | PebKOR4 | PebKOR3 | 60 |
| PebCESA9 | PebCESA2 | 76.417 | PebKOB1 | PebKOB2 | 68.421 |
| PebCESA9 | PebCESA7 | 64.266 | PebKOB2 | PebKOB1 | 59.949 |
| PebCESA9 | PebCESA10 | 65.486 | PebCTL1 | PebCTL2 | 90.823 |
| PebCESA9 | PebCESA11 | 65.213 | PebCTL1 | PebCTL3 | 85.535 |
|  |  |  | PebCTL2 | PebCTL3 | 86.306 |

**Table S4.** The ANOVA (Duncan’s multiple range test) results of the relative expression of 19 core genes in leaves under different nitrogen treatments.

| Treatment | N0 | N1 | N2 | N3 |
| --- | --- | --- | --- | --- |
| *PebCESA1* | 0.1015 ± 0.0054^a^ | 0.0337 ± 0.0026^c^ | 0.0213 ± 0.0023^d^ | 0.0520 ± 0.0001^b^ |
| *PebCESA5* | 0.5497 ± 0.0193^a^ | 0.4254 ± 0.0321^b^ | 0.3331 ± 0.0084^c^ | 0.3429 ± 0.0392^c^ |
| *PebCESA7* | 0.0646 ± 0.0053^a^ | 0.0495 ± 0.0027^c^ | 0.0560 ± 0.0043^bc^ | 0.0573 ± 0.0029^ab^ |
| *PebCESA8* | 0.2343 ± 0.0087^a^ | 0.1433 ± 0.0057^c^ | 0.1379 ± 0.0101^c^ | 0.1963 ± 0.0085^b^ |
| *PebCESA11* | 0.1944 ± 0.0194^b^ | 0.2576 ± 0.0215^a^ | 0.2088 ± 0.0163^b^ | 0.2268 ± 0.0269^ab^ |
| *PebCOB2* | 0.1068 ± 0.0113^a^ | 0.0830 ± 0.0040^b^ | 0.1002 ± 0.0069^a^ | 0.1158 ± 0.0098^a^ |
| *PebCOB3* | 0.0799 ± 0.0019^b^ | 0.1034 ± 0.0055^a^ | 0.0207 ± 0.0017^c^ | 0.0211 ± 0.0026^c^ |
| *PebCTL2* | 0.5830 ± 0.0327^a^ | 0.4297 ± 0.0423^b^ | 0.5100 ± 0.0261^a^ | 0.5519 ± 0.0584^a^ |
| *PebCTL3* | 0.0551 ± 0.0020^c^ | 0.0689 ± 0.0029^b^ | 0.1004 ± 0.0058^a^ | 0.0513 ± 0.0013^c^ |
| *PebKOB1* | 0.0278 ± 0.0011^ab^ | 0.0310 ± 0.0034^a^ | 0.0228 ± 0.0018^c^ | 0.0249 ± 0.0026^bc^ |
| *PebKOR1* | 0.0596 ± 0.0057^a^ | 0.0371 ± 0.0037^b^ | 0.0232 ± 0.0001^c^ | 0.0214 ± 0.0022^c^ |
| *PebKOR2* | 0.1121 ± 0.0123^a^ | 0.0789 ± 0.0065^b^ | 0.0799 ± 0.0128^b^ | 0.1135 ± 0.006^a^ |
| *PebKOR4* | 0.0400 ± 0.0039^a^ | 0.0269 ± 0.0009^b^ | 0.0387 ± 0.0056^a^ | 0.0400 ± 0.0028^a^ |
| *PebCC1* | 0.0626 ± 0.0063^ab^ | 0.0556 ± 0.0027^b^ | 0.0629 ± 0.0011^ab^ | 0.0712 ± 0.0072^a^ |
| *PebCC3* | 0.1242 ± 0.0065^a^ | 0.0601 ± 0.0034^b^ | 0.0527 ± 0.0030^b^ | 0.0510 ± 0.0063^b^ |
| *PebCC5* | 0.0262 ± 0.0027^b^ | 0.0203 ± 0.0018^c^ | 0.0286 ± 0.0014^b^ | 0.0383 ± 0.0045^a^ |
| *PebCSI1* | 0.0186 ± 0.0013^a^ | 0.0127 ± 0.0109^a^ | 0.0235 ± 0.0020^a^ | 0.0238 ± 0.0007^a^ |
| *PebCSI2* | 0.0457 ± 0.0042^a^ | 0.0235 ± 0.0036^b^ | 0.0135 ± 0.0048^c^ | 0.0254 ± 0.0010^b^ |
| *PebSTL2* | 0.0050 ± 0.0002^a^ | 0.0027 ± 0.0001^b^ | 0.0017 ± 0.0002^c^ | 0.0013 ± 0.0001^d^ |

**Table S5**. List of plant genome sequences used in the comparative genomic analysis.

| Species | Genome annotation version | Link |
| --- | --- | --- |
| *Arabidopsis thaliana* | TAIR10 | ftp://ftp.arabidopsis.org/home/tair/Sequences/blast_datasets/TAIR10_blastsets/ |
| *Brassica rapa* | FPsc v1.3 | https://phytozome-next.jgi.doe.gov/info/BrapaFPsc_v1_3 |
| *Cucumis sativus* | v1.0 | ftp://cucurbitgenomics.org/pub/cucurbit/genome/cucumber/Chinese_long/ |
| *Daucus carota* | v2.0 | https://phytozome-next.jgi.doe.gov/info/Dcarota_v2_0 |
| *Medicago truncatula* | MedtrA17_4.0 | https://plants.ensembl.org/Medicago_truncatula/Info/true |
| *Spinacia oleracea* | Monoe-Viroflay | http://spinachbase.org/ftp/genome/Monoe-Viroflay/ |
| Species | Reference |  |
| *Arabidopsis thaliana* | Arabidopsis Genome Initiative. 2000. Analysis of the genome sequence of the flowering plant *Arabidopsis thaliana*. *Nature* 408: 796-815. | |
| *Brassica rapa* | The Brassica rapa Genome Sequencing Project Consortium. 2011. The genome of the mesopolyploid crop species *Brassica rapa*. Nature Genetics. 43: 1035–1039. | |
| *Cucumis sativus* | Li, Q., et al. 2019. A chromosome-scale genome assembly of cucumber (*Cucumis sativus* L.). GigaScience. 8: giz072. | |
| *Daucus carota* | Iorizzo, M., et al. 2016. A high-quality carrot genome assembly provides new insights into carotenoid accumulation and asterid genome evolution. Nature Genetics. 48: 657–666. | |
| *Medicago truncatula* | Pecrix, Y., et al. 2018. Whole-genome landscape of Medicago truncatula symbiotic genes. 4: 1017–1025. | |
| *Spinacia oleracea* | Cai, X., et al. 2021. Genomic analyses provide insights into spinach domestication and the genetic basis of agronomic traits. 12: 7246. | |

**Table S6.** **Information on primers used for qRT-PCR.**

| Gene name | Product Length | Sense Primer | Tm | Anti-sense Primer | Tm |
| --- | --- | --- | --- | --- | --- |
| PebCESA1 | 110 | TGGCGATTCTTGGGTTGTTCTTCC | 60.1 | CCACGACACAGCGAACCATATCTC | 60.1 |
| PebCESA5 | 106 | ATCCAATGAAAGAGCCGCCACTT | 59.3 | GCACCATCGTCCGAGACATAGC | 59.5 |
| PebCESA7 | 152 | ACCGGACTCCTACAATCGTC | 57.8 | GAGGTTCCCGACATGACCCT | 60.8 |
| PebCESA8 | 177 | CCTAACAGAAGAAGACCTGCA | 56.3 | TCGTATCCTTCCACGCCATC | 60.6 |
| PebCESA11 | 152 | AGATGCGGTGGAGCGGTGTT | 60.2 | CTTGGACGTGACAGTGAAGTTGGT | 59.7 |
| PebCSI1 | 227 | GCGAGAAGGTAGTGCGGCAAAT | 59.9 | CGGCAAGGCAACGGCAAGAT | 60 |
| PebCSI2 | 102 | AGAGTTCGGACCAGACGGACAA | 59.3 | GCTTGCTGACCTGTGAGAGTGT | 58.8 |
| PebCC1 | 100 | CGGTGGCTGGCTTCCTTCTTCT | 60.8 | ACGGACAGACTCTTGACGGCAA | 60.2 |
| PebCC3 | 129 | CTGCGAGTAAGCCTCAGAAGCC | 59.4 | AAGCAACCGTGGAGTTCAAGGA | 58.4 |
| PebCC5 | 195 | CTGTTCGGAGCTTGTCGGTCAA | 59.2 | GCTGTCCTGTGGCAACAACAAC | 59.1 |
| PebSTL2 | 219 | ACCAGCAGCCTCGTCTGATGT | 59.6 | TCCGTTCCACAGGTCCATAGCA | 59.4 |
| PebCOB2 | 149 | ACACCACCGCCGTCTCTTCT | 59.2 | GCCATCGTTGCCGTGTTGTAATC | 59.5 |
| PebCOB3 | 143 | CCTGATGGCTATGTTGCGGTTGTA | 59.7 | CCTTGCTCAGTGGCTTGACCTC | 59.5 |
| PebKOR1 | 161 | TTGTGATGCAGGTCGGAGAAGGT | 60.3 | AGAAGCCAAAGCAGCAGCCATTT | 60.3 |
| PebKOR2 | 180 | TGTCATAGTTGCTCCGACCTTGCT | 60.6 | ATAGAATGTTGCCGCCTCGTTACC | 59.8 |
| PebKOR3 | 157 | AGATTCCGCCACCGACGATGA | 59.8 | GCTCACGATAACGCATCCGAGAT | 59.4 |
| PebKOB1 | 156 | TGCTGGCATGGACTGGATACTTCA | 60.4 | TCATCTCGTTCGACACTGCTCTCA | 60.2 |
| PebCTL2 | 128 | ATCTTGGTTGCGTTGATGGTGGTT | 60.0 | TGCTCCGAGAAGCCCTCACATT | 60 |
| PebCTL3 | 199 | AGTTGGTGGGAAGAGGATGTGTGA | 60.0 | CGGCTGATAAACGGCGGAAGAC | 60.3 |
