# Supplementary material for: Genome-wide analysis of the cellulose toolbox of Primulina eburnea, a calcium-rich vegetable
Source: BMC Plant Biol. 2023 May 16;23:259. doi: 10.1186/s12870-023-04266-z (PMC10186795; doi:10.1186/s12870-023-04266-z)
Supplement: Supplementary file 3 — Supplementary Material 3 [file 12870_2023_4266_MOESM3_ESM.pdf]

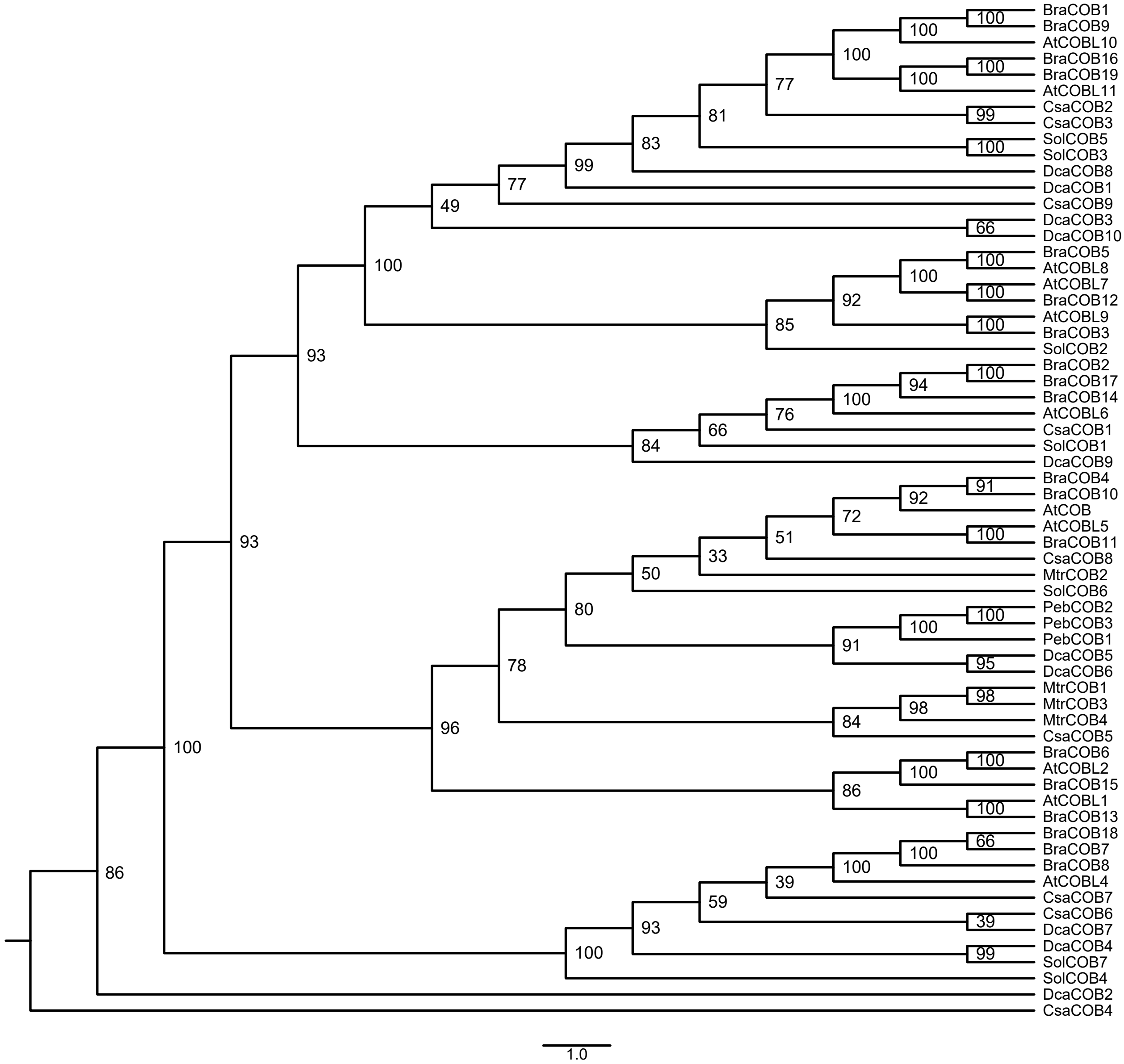
BraCOB1
BraCOB9
AtCOBL10
BraCOB16
BraCOB19
AtCOBL11
CsaCOB2
CsaCOB3
SolCOB5
SolCOB3
DcaCOB8
DcaCOB1
CsaCOB9
DcaCOB3
DcaCOB10
BraCOB5
AtCOBL8
AtCOBL7
BraCOB12
AtCOBL9
BraCOB3
SolCOB2
BraCOB2
BraCOB17
BraCOB14
AtCOBL6
CsaCOB1
SolCOB1
DcaCOB9
BraCOB4
BraCOB10
AtCOB
AtCOBL5
BraCOB11
CsaCOB8
MtrCOB2
SolCOB6
PebCOB2
PebCOB3
PebCOB1
DcaCOB5
DcaCOB6
MtrCOB1
MtrCOB3
MtrCOB4
CsaCOB5
BraCOB6
AtCOBL2
BraCOB15
AtCOBL1
BraCOB13
BraCOB18
BraCOB7
BraCOB8
AtCOBL4
CsaCOB7
CsaCOB6
DcaCOB7
DcaCOB4
SolCOB7
SolCOB4
DcaCOB2
CsaCOB4
1.0
